# Comparative safety and effectiveness of cholinesterase inhibitors and memantine for Alzheimer’s disease: a network meta-analysis of 41 randomized controlled trials

**DOI:** 10.1186/s13195-018-0457-9

**Published:** 2018-12-27

**Authors:** Kai-Xin Dou, Meng-Shan Tan, Chen-Chen Tan, Xi-Peng Cao, Xiao-He Hou, Qi-Hao Guo, Lan Tan, Vincent Mok, Jin-Tai Yu

**Affiliations:** 1 Department of Neurology, Qingdao Municipal Hospital, Qingdao University, No. 5 Donghai Middle Road, Qingdao, China; 2 Clinical Research Center, Qingdao Municipal Hospital, Qingdao University, Qingdao, China; 3 Department of Neurology & Institute of Neurology, Huashan Hospital, Fudan University, WHO Collaborating Center for Research and Training in Neurosciences, Shanghai, China; 4 Division of Neurology, Department of Medicine and Therapeutics, Faculty of Medicine, The Chinese University of Hong Kong, Shatin, New Territories Hong Kong, China; 5 Therese Pei Fong Chow Research Center for Prevention of Dementia, The Chinese University of Hong Kong, Shatin, New Territories Hong Kong, China; 6 Gerald Choa Neuroscience Centre, Lui Che Woo Institute of Innovative Medicine, The Chinese University of Hong Kong, Shatin, New Territories Hong Kong, China; 7 Department of Neurology, Huashan Hospital, Fudan University, No. 12 Wulumuqi Road, Shanghai, China

**Keywords:** Alzheimer’s disease, Cholinesterase inhibitors, Memantine, Network meta-analysis

## Abstract

**Background:**

Cholinesterase inhibitors and memantine have been approved for management of Alzheimer’s disease (AD), but there has been no consensus about the choice of various types and doses of drugs at different stages. Hence, we compared and ranked the efficacy and tolerability of these available drugs.

**Methods:**

We searched PubMed, the Cochrane Central Register of Controlled Trials, and Embase for randomized controlled trials (RCTs) published from database inception to July 21, 2017. The primary outcomes were the mean overall changes in cognitive function and responders who had any adverse events. We conducted a random-effects network meta-analysis.

**Results:**

Forty-one RCTs were included in this study. Compared with placebo, galantamine 32 mg daily (standardized mean difference – 0.51, 95% credible interval – 0.67 to − 0.35), galantamine 24 mg daily (− 0.50, − 0.61 to − 0.40), and donepezil 10 mg daily (− 0.40, − 0.51 to − 0.29) were probably the most effective agents on cognition for mild to moderate AD, and memantine 20 mg combined with donepezil 10 mg (0.76, 0.39 to 1.11) was recommended for moderate to severe patients. Memantine showed the best profile of acceptability. Rivastigmine transdermal 15-cm^2^ patch was the best optional treatment both in function and global changes. None of the medicines was likely to improve neuropsychiatric symptoms through this analysis.

**Conclusions:**

Pharmacological interventions have beneficial effects on cognition, function, and global changes, but not on neuropsychiatric symptoms, through current network meta-analysis. The choice of drugs may mainly depend on the disease severity and clinical symptoms.

**Electronic supplementary material:**

The online version of this article (10.1186/s13195-018-0457-9) contains supplementary material, which is available to authorized users.

## Background

Alzheimer’s disease (AD), a progressive neurodegenerative disorder, is the most common form of dementia affecting 46.8 million people with an enormous public health impact [1, 2]. Primary manifestations of AD include progressive deterioration of cognition, impairment in functional ability, and alterations of neuropsychiatric symptoms. Currently, there are no therapeutic interventions that can delay the disease progression, but available medications have provided symptomatic benefits [3]. Two main classes of drugs are recommended by the US Food and Drug Administration (FDA) for pharmacological management of AD: cholinesterase inhibitors (ChEIs) donepezil, galantamine, and rivastigmine, which are licensed for mild to moderate AD [4, 5]; and glutamate antagonist memantine for moderate to severe stage [6]. Donepezil is the only ChEI to be indicated for use all across the full spectrum of AD [7]. Owing to fewer treatment options for more serious patients, donepezil 23 mg was approved for moderate to severe AD in recent years [8, 9] and the combination therapy of donepezil plus memantine is proposed for treating patients in this stage [10].

Previous meta-analyses focused only on comparing the efficacy and tolerability of drugs with placebo, which gave the clinical application directions [6, 11, 12]. Those findings showed modest benefits for improving the symptoms related to cognition, function, behavior, and clinical global changes [13, 14]. But considering the lack of direct comparative evidence among available drugs, the results of those studies were inconclusive on how to choose the optimal therapeutic regimens to achieve the maximum efficiency [15]. The network meta-analysis combines evidence from a network of all included trials to rank all available treatments in terms of efficacy and tolerability, providing estimates for interventions even if they have not been directly compared [16]. Here, we therefore conducted a network meta-analysis to comprehensively compare and rank different types and dosages of cognitive enhancers at different clinical stages for guiding treatment decisions.

## Methods

### Search strategy and selection criteria

In this network meta-analysis, potentially eligible randomized controlled trials (RCTs) were searched through PubMed, Embase, and the Cochrane Central Register of Controlled Trials, which were published between database inception and July 21, 2017. Additional trials were retrieved from the cited references of relevant published meta-analyses and systematic reviews. Included studies were completed RCTs with English language publication that met the following criteria: only double-blind RCTs with follow-up of 12–104 weeks; the trials compared four primary FDA-approved treatments (donepezil, galantamine, rivastigmine, or memantine) alone or in combination (only including the donepezil–memantine combination approved by the FDA) with placebo or other treatments; drug dosages were specific and within the therapeutic range; eligible participants had a clinical diagnosis based on the *Diagnostic and Statistical Manual of Mental Disorders* (DSM) for dementia of the Alzheimer’s type or the National Institute of Neurological and Communicative Disorders and Stroke and the Alzheimer's Disease and Related Disorders Association (NINCDS-ADRDA) for probable AD [17]; criteria for disease severity classification were reasonable; and at least one of the five outcomes of cognition, function, behavior, global assessment, or adverse events was covered. We excluded quasi-randomized trials, trials with too short-term or too long-term follow-up (< 12 weeks or > 104 weeks), trials that included patients with mixed dementia or neuropsychiatric symptoms, and trials that recruited fewer than 10 participants per group.

### Data extraction and quality assessment

Basic study characteristics—for instance, sample size, age, gender, race, drug dosage, disease severity, diagnostic criteria, trial duration, cognitive scores of baseline level, efficacy outcomes of the change from baseline, and individuals who experienced all-cause adverse events—were extracted from each trial. For all of the studies included, we analyzed the intention-to-treat (ITT) population results if the trials adopted the ITT approach [18]. Three investigators (K-XD, M-ST, and C-CT) independently abstracted information from original articles with the standardized data extraction table. If no consensus was reached, further discussion would be carried out with other members of the team or authors. We appraised the risk of bias using the Cochrane Risk of Bias Tool [19].

### Outcomes

Measurement scales that were used in the trials were different from each other. We paid close attention to cognitive function (the mean overall changes from baseline to endpoint) and tolerability (the responders who had any adverse events during the treatment period) for the primary outcomes. Efficacy in cognition was mainly evaluated by the Alzheimer’s Disease Assessment Scale—cognition subscale (ADAS-cog), the Severe Impairment Battery (SIB), and the Mini-Mental State Examination (MMSE). Secondary outcomes included daily functions assessed by the Alzheimer’s Disease Cooperative Study—Activities of Daily Living (ADCS-ADL) and the Bristol Activities of Daily Living Scale (BADLS), neuropsychiatric symptoms assessed by the Neuropsychiatric Inventory (NPI), and the global assessment of changes assessed by the Clinician’s Interview Based Impression of Change Plus Caregiver Input (CIBIC plus) and the Clinical Global Impression of Change (CGIC). Based on all included RCTs in this network meta-analysis, we summarized that most of the patients with mild to moderate AD have MMSE scores of 10–26 and those with moderate-to-severe AD have scores of 0–15.

### Statistical analysis

At first, we conducted a pairwise meta-analysis using the random-effects model. We chose the standardized mean difference (SMD) with 95% confidence interval (CI) as the effect sizes for continuous results, while dichotomous results were calculated using pooled odds ratios (ORs). We quantitatively investigated the statistical heterogeneity in each direct comparison using the *I*^2^ statistic and *P* value. Publication bias was examined with the Egger’s regression test. The pairwise meta-analysis was conducted in STATA (version 14.1) software.

Secondly, we performed a random-effects model Bayesian network meta-analysis using WinBUGS software (version 1.4.3; MRC Biostatistics Unit, Cambridge, UK) and drew relevant diagrams with STATA [20]. We summarized the results of the network meta-analysis by choosing the SMD or OR with corresponding credible intervals (CrIs) as effect sizes. We adopted noninformative priors and changed the precision of the prior distribution in sensitivity analyses. Details about the WinBUGS codes are presented in Additional file 1: Supplementary 1. The global heterogeneity of network meta-analyses was assessed by the *I*^*2*^ statistic with the gemtc R package (version 3.2.2). Inconsistency was statistically examined by calculating the significant discrepancies between direct and indirect evidence in each closed loop with the loop-specific method and the node splitting method [21–23]. The intervention hierarchy was estimated and expressed by rankograms, the surface under the cumulative ranking curve (SUCRA), and mean ranks [24]. The comparison-adjusted funnel plot could be drawn to detect publication bias in the network meta-analysis [24].

Study characteristics, the main source of heterogeneity, might affect the accuracy of the results. Correspondingly, we conducted the subgroup network meta-analyses for secondary outcomes to adjust for the difference in severity of disease. Additionally, sensitivity network meta-analyses were conducted to examine the robustness of primary outcomes by omitting short-term or longer follow-up studies (only including studies with 20–30 weeks of follow-up).

## Results

### Literature search findings

**Fig. 1:**
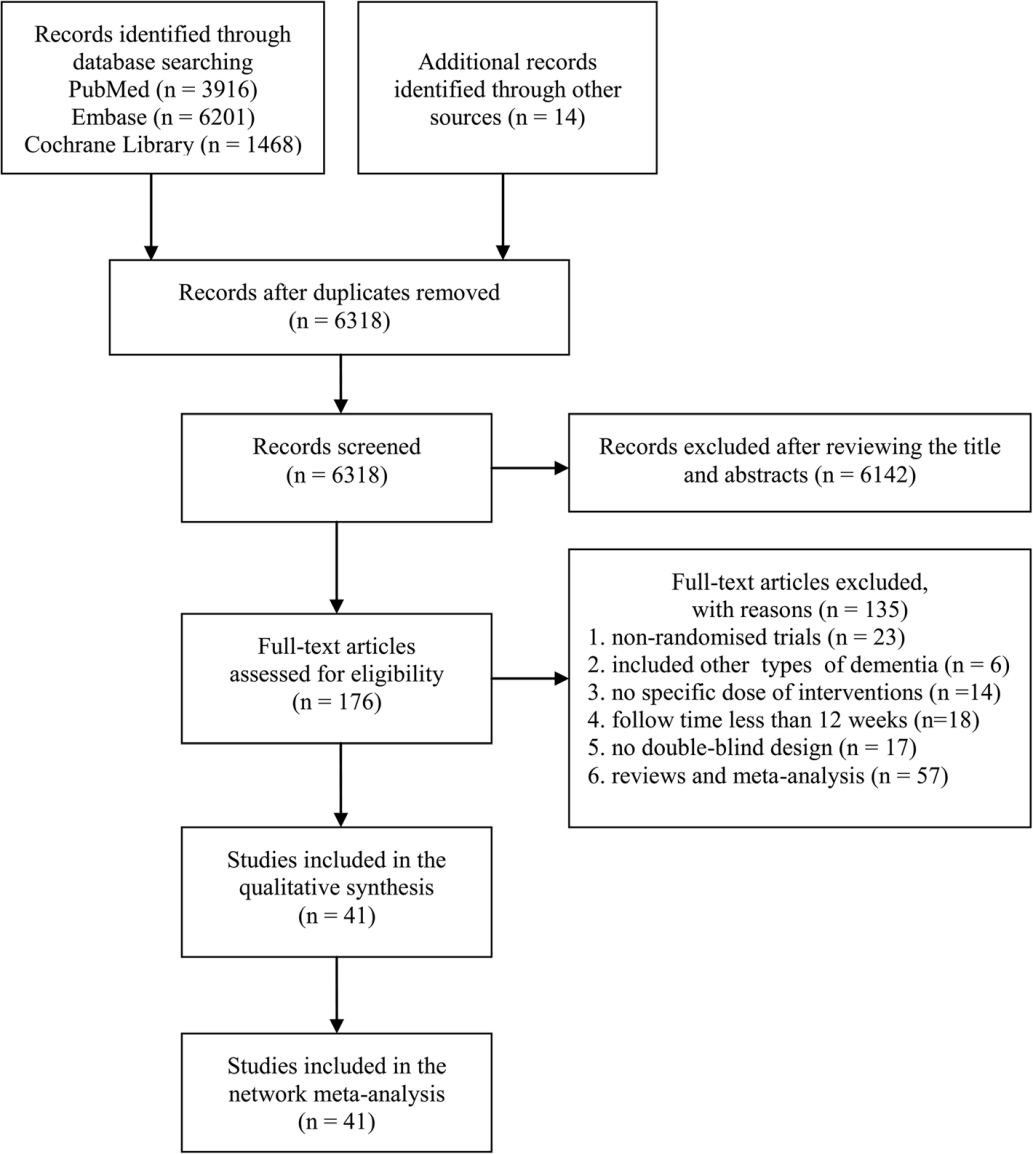
Flow diagram of study selection

Through electronic searches, we identified 11,599 citations and assessed 176 full-text articles for potentially eligibility. We excluded 135 studies and the remaining 41 studies were included for the network meta-analysis (Fig. 1). Overall, we used eligible double-blind RCTs published from 1998 to 2016, which enrolled 18,898 individuals. The detailed lists of the included studies and clinical characteristics are presented in Additional file 1: Supplementary 2 and 3. The studies were mainly conducted in European and American countries (80.4%) and about 64.7% of participants were women. The mean period was 28.7 (between 12 and 104) weeks and the average number of individuals was 207 (range 11–1024) in each group. Only five trials had a follow-up time shorter than 20 weeks. As for the risk of bias, most RCTs (73.2%) had a low risk of bias for random sequence generation and incomplete outcome data (87.8%). However, 60.1% of studies were rated as unclear risk of bias for blinding of participants and personnel, and 82.9% for selective outcome reporting (Additional file 1: Supplementary 4).

### Pairwise meta-analysis

Detailed results of pairwise meta-analyses are presented in Additional file 1: Supplementary 5. For assessment of cognition, all drugs except for rivastigmine 5-cm^2^ patch were significantly more efficacious than placebo for mild to moderate AD; the combination therapy of memantine 20 mg with donepezil 10 mg, donepezil 10 mg daily alone, and memantine 20 mg daily alone were superior to placebo for moderate to severe AD. In terms of tolerability, donepezil 10 mg daily, galantamine (24 and 32 mg), and rivastigmine (12 mg oral and 5-cm^2^ patch) were less well tolerated than placebo for mild to moderate AD; donepezil 10 mg daily was less well tolerated than placebo for moderate to severe AD. In terms of activities of daily living, the combination therapy of memantine 20 mg with donepezil 10 mg, donepezil 10 mg daily alone, galantamine 24 mg daily alone, and rivastigmine 10-cm^2^ patch alone were more efficacious than placebo. For assessment of global changes, donepezil (5 and 10 mg), galantamine 24 mg, and rivastigmine 12 mg were superior to placebo. Only galantamine 24 mg significantly improved in neuropsychiatric symptoms compared with placebo. Overall, the heterogeneity of direct comparisons was moderate (*I*^2^ < 50% for most comparisons, Additional file 1: Supplementary 5). However, we found *I*^2^ > 70% for direct comparisons of donepezil 10 mg vs donepezil 23 mg (*I*^2^ = 72.3%) and galantamine 24 mg vs galantamine 32 mg (*I*^2^ = 71.1%).

### Network meta-analysis—primary outcomes

#### Cognitive functions for mild to moderate AD

**Fig. 2:**
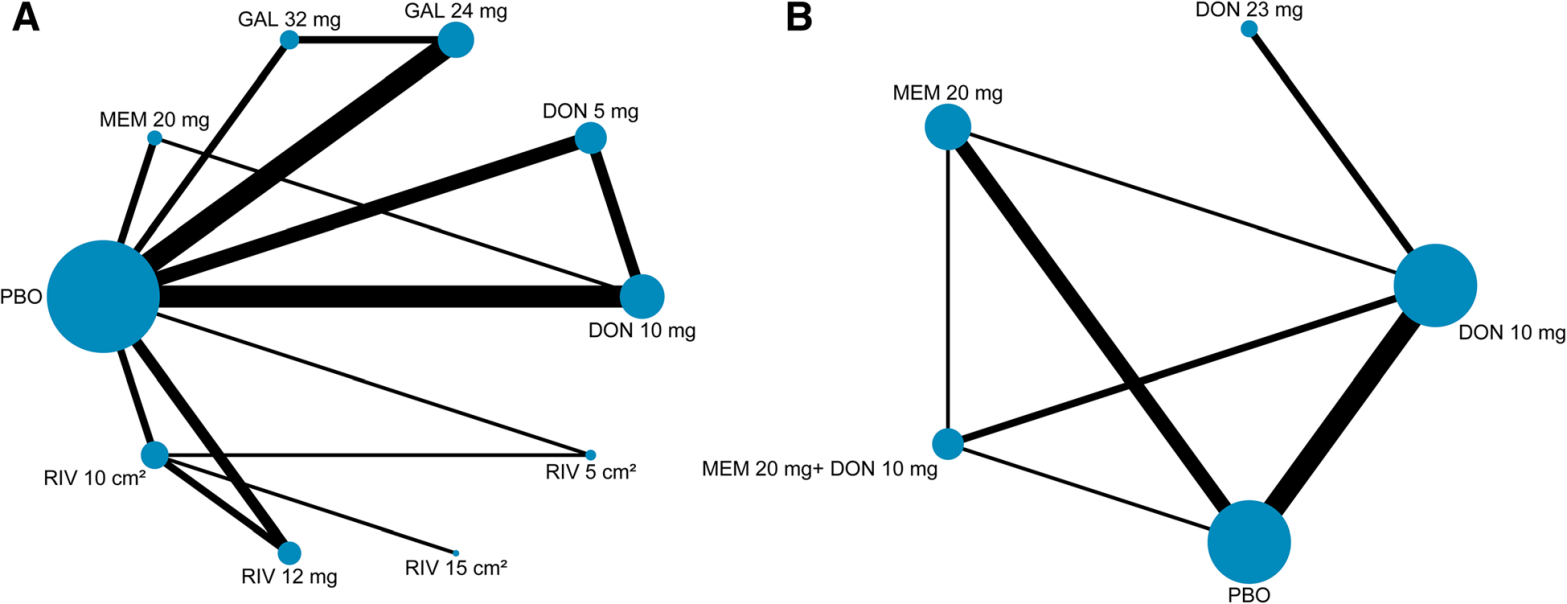
Network of eligible comparisons of cognition for mild to moderate AD (**a**) and moderate to severe AD (**b**). Width of lines proportional to number of trials comparing every pair of treatments, and size of every node proportional to number of randomly assigned participants (sample size). DON donepezil, GAL galantamine, MEM memantine, PBO placebo, RIV rivastigmine

**Fig. 3:**
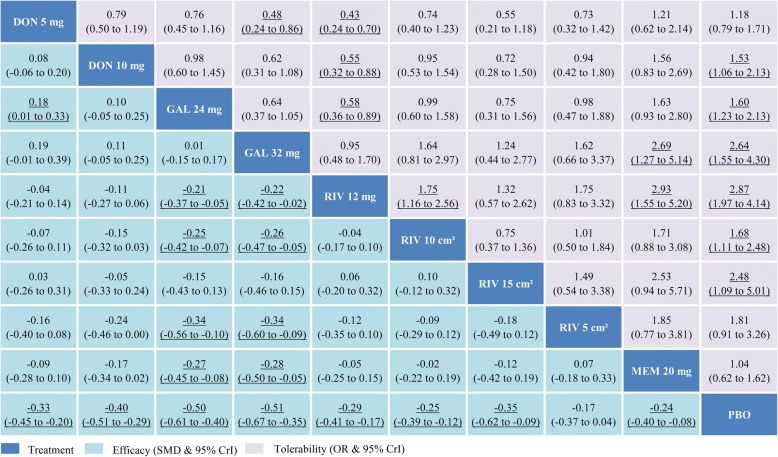
Comparative efficacy and tolerability for mild to moderate AD in the network meta-analysis: comparisons should be read from left to right. Efficacy and tolerability estimate located at intersection of column-defining treatment and row-defining treatment. For efficacy (mean changes of cognition), SMD < 0 favors column-defining treatment. For tolerability (all-cause adverse events), OR < 1 favors row-defining treatment. Significant results bold and underlined. CrI credible interval, DON donepezil, GAL galantamine, MEM memantine, OR odds ratio, PBO placebo, RIV rivastigmine, SMD standardized mean difference

**Fig. 4:**
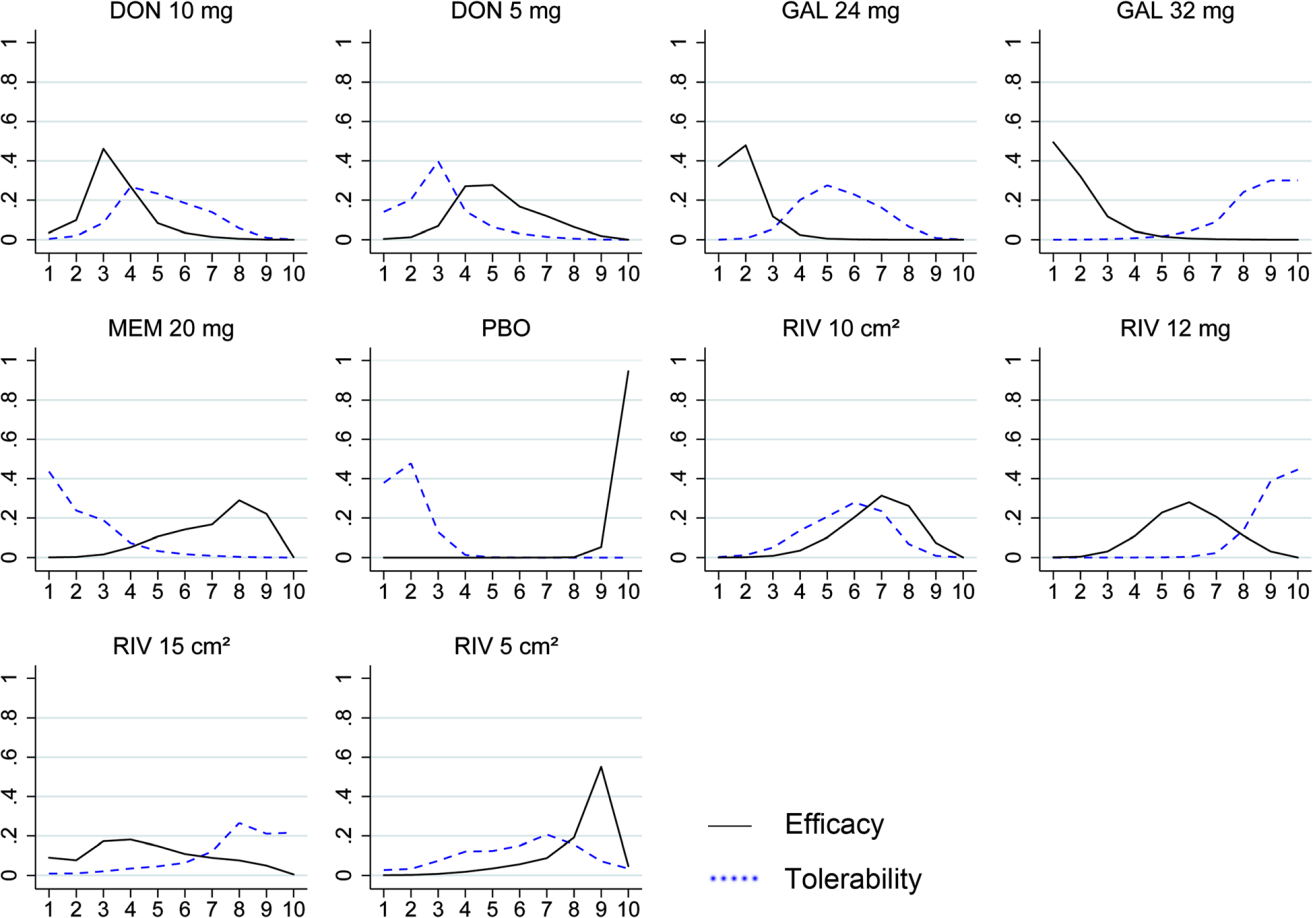
Rank for efficacy on cognition (solid line) and tolerability (dotted line) for mild to moderate AD. Ranking (*x* axis) indicates probability to be best treatment, second best, third best, and so on. DON donepezil, GAL galantamine, MEM memantine, PBO placebo, RIV rivastigmine

In our network meta-analysis, 22 trials assessed mean changes of cognition based on the ADAS-cog scale for mild to moderate AD. The network plot of comparisons among available drugs in cognition for mild to moderate patients is shown in Fig. 2a. All interventions, except for rivastigmine 15-cm^2^ patch, had at least one placebo-controlled comparison and all drugs were directly compared with at least one other treatment. The results for the primary outcomes among the mild to moderate patients are displayed in a league table format (Fig. 3). All drugs except for rivastigmine 5-cm^2^ patch were significantly more efficacious than placebo, with SMDs ranging between − 0.51 (95% CrI − 0.67 to − 0.35) for galantamine 32 mg daily and − 0.24 (− 0.40 to − 0.08) for memantine 20 mg daily. Both galantamine 24 mg and 32 mg were superior to four other therapeutic regimens (rivastigmine 12 mg, 5-cm^2^ patch, and 10-cm^2^ patch and memantine 20 mg); otherwise, galantamine 24 mg daily was superior to donepezil 5 mg (SMD − 0.18, 95% CrI − 0.33 to − 0.01). Figure 4 shows the probabilities of 10 therapy regimens ranked in order. Galantamine 32 mg daily had the greatest probability to be the best choice to enhance cognitive performance according to the SUCRA (91.1%). The therapies of galantamine 24 mg (90.9%), donepezil 10 mg (72.8%), and rivastigmine 15-cm^2^ patch (59.8%) followed close behind in second, third, and fourth.

#### Cognitive functions for moderate to severe AD

For assessment of cognitive function for moderate to severe patients, the SIB scale and the MMSE were used in 12 studies to assess five treatment regimens (Fig. 2b). The combination therapy of memantine 20 mg daily plus donepezil 10 mg daily (SMD 0.76, 95% CrI 0.39 to 1.11), donepezil 23 mg daily alone (0.60, 0.21 to 0.99), donepezil 10 mg daily alone (0.53, 0.33 to 0.75), and memantine 20 mg daily alone (0.34, 0.08 to 0.63) showed statistical significance compared with placebo, but no significant differences were observed between either of the two active drugs (Additional file 1: Supplementary 6). The combination therapy had the greatest probability to be the best treatment according to the SUCRA (91.5%), followed by donepezil 23 mg daily with the greatest probability (69.6%) in second position, and then donepezil 10 mg daily (57.3%) and memantine 20 mg daily (31.3%), see Additional file 1: Supplementary 8.

#### Tolerability for mild to moderate AD

In terms of tolerability, 21 RCTs with 10 interventions provided data on participants experiencing any adverse events in mild to moderate stage. The network plot was presented in the Additional file 1: Supplementary 7. The network meta-analysis revealed that no active drugs were more tolerable than placebo and only memantine 20 mg daily, donepezil 5 mg daily, and rivastigmine 5-cm^2^ patch did not present a significantly higher risk of adverse events than placebo (Fig. 3). Rivastigmine 12 mg daily had a significantly higher risk of adverse events than placebo (OR 2.87, 95% CrI 1.97 to 4.14) and five other therapeutic regimens (donepezil 5 mg, donepzeil 10 mg, galantamine 24 mg, rivastigmine 10-cm^2^ patch, and memantine 20 mg). Probably, the best acceptable regimen was memantine 20 mg daily with a SUCRA of 87.3%, followed by donepezil 5 mg (78.1%), donepezil 10 mg (53.7%), and then galantamine 24 mg (50.4%). Rivastigmine 12 mg daily with a SUCRA of 8.4% and galantamine 32 mg (15.2%) had the highest probability of being ranked in the last two positions (Fig. 4).

#### Tolerability for moderate to severe AD

For assessment of tolerability for moderate to severe patients, only 10 studies with four treatments were evaluated. All drugs except for memantine 20 mg daily had a significantly higher risk of potentially adverse reactions than placebo (ORs ranging from 1.56 to 2.63). Donepezil 23 mg was less well tolerated than donepezil 10 mg (OR 1.69, 95% CrI 1.20 to 2.35) and memantine 20 mg daily (2.56, 1.35 to 4.50). Memantine 20 mg showed the highest probability (SUCRA 83.5%) of receiving the highest treatment acceptability rating, followed by donepezil 10 mg (50.3%) and memantine 20 mg in combination with donepezil 10 mg (21.9%), while donepezil 23 mg was ranked in last position (4.5%).

### Network meta-analysis—secondary outcomes

**Fig. 5:**
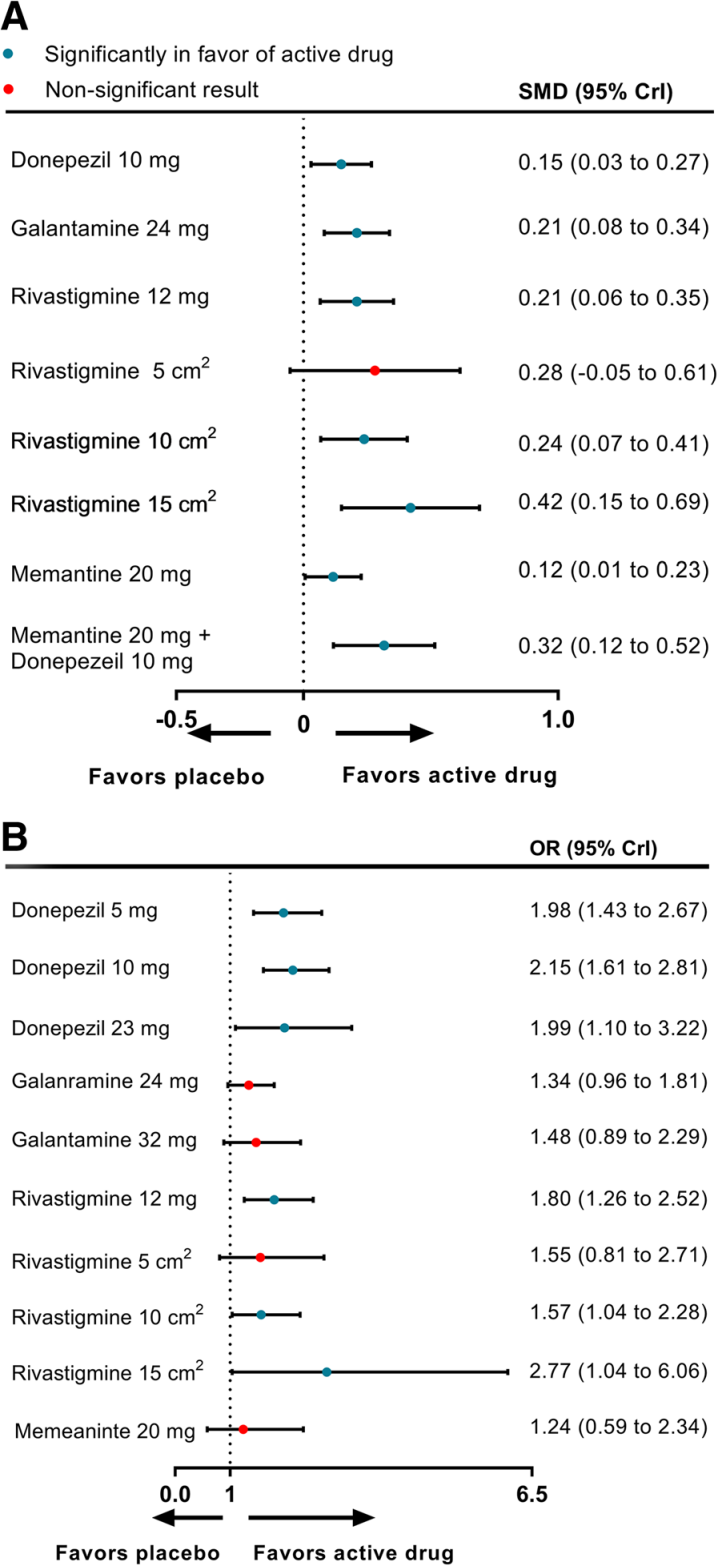
Forest plots of network meta-analysis of efficacy on function (**a**) and global changes (**b**) compared with placebo. SMD standardized mean difference, OR odds ratio, CrI credible interval

In terms of secondary outcomes, 20 studies with nine treatments reported data on the activities of daily living according to the ADL and BADLS scale. All drugs except for rivastigmine 5-cm^2^ patch were significantly more efficacious than placebo, with SMDs ranging between 0.12 (95% CrI 0.01 to 0.23) for memantine 20 mg daily and 0.42 (0.15 to 0.69) for rivastigmine 15-cm^2^ patch (Fig. 5a). Rivastigmine 15-cm^2^ patch was significantly superior to memantine 20 mg daily (SMD 0.31, 95% CrI 0.01 to 0.59). Rivastigmine 15-cm^2^ patch was likely to be the best treatment to improve activities of daily living (SUCRA 93.2%), followed by memantine 20 mg in combination with donepezil 10 mg (76.4%). Sixteen studies measured the mean changes on the NPI scale for the efficacy of neuropsychiatric symptoms, which evaluated five different therapy regimens and placebo. The network meta-analysis indicated that all interventions did not have significant improvements in neuropsychiatric symptoms compared with placebo. The assessment of clinical global changes with 22 eligible studies and 11 treatments on the CIBIC+ scale and the CGIC scale indicated that donepezil 5 mg, 10 mg, and 23 mg were significantly more efficacious than placebo (ORs ranging between 1.98 and 2.15); rivastigmine 12 mg, 10-cm^2^ patch, and 15-cm^2^ patch were superior to placebo (ORs ranging between 1.57 and 2.77). The results are shown in Fig. 5b. Rivastigmine 15-cm^2^ patch received the highest treatment acceptability rating according to the SUCRA (83.6%), followed by donepezil 10 mg (81.7%) and donepezil 5 mg (71.3%).

### Subgroup analyses and sensitivity analyses

We estimated the effects on different severities of disease for secondary outcomes in subgroup analyses (Additional file 1: Supplementary 11). For assessment of activities of daily living, rivastigmine 15-cm^2^ patch (SMD 0.41, 95% CrI 0.02 to 0.81) and donepezil 10 mg (0.25, 0.04 to 0.46) were better than placebo on function for mild to moderate AD; the combination therapy of memantine 20 mg with donepezil 10 mg and donepezil 10 mg daily alone showed the superiority in activities of daily living in moderate to severe AD. The results on global changes were similar to those of the main analyses and no statistical difference was observed between active drugs and placebo on neuropsychiatric symptoms. The results of sensitivity analyses did not affect the main results by omitting short-term or longer follow-up trials (Additional file 1: Supplementary 12).

### Consistency analyses and heterogeneity analyses

There was statistical incoherence between the comparison of donepezil 5 mg daily with placebo and that of donepezil 5 mg with donepezil 10 mg for mild to moderate AD by the node-splitting method using direct and indirect evidence (Additional file 1: Supplementary 9). In heterogeneity analyses, we found that the global *I*^2^ value was higher than 75% in cognition among the population with moderate to severe AD (80.12%, Additional file 1: Supplementary 13). The comparison-adjusted funnel plots of the network meta-analysis showed a relatively symmetrical distribution and suggested that publication bias did not exist (Additional file 1: Supplementary 10).

## Discussion

This network meta-analysis comprehensively compares and ranks efficacy and tolerability among current available cognitive enhancers approved by the FDA for AD. The cholinesterase inhibitors result in better outcomes on cognition than memantine for mild to moderate patients, among which galantamine and donepezil are probably the interventions most strongly associated with cognitive improvements. For moderate to severe AD, the combination therapy of donepezil 10 mg with memantine 20 mg is the most effective regimen, followed by donepezil 23 mg alone. However, none of the cognitive enhancers was likely to improve behavior. The higher-dose rivastigmine transdermal patch (15 cm^2^) was probably the best option considering the benefits of both function and clinical global impression. Memantine shows the best profile of acceptability, while rivastigmine oral form is associated with a high incidence of adverse events. Both clinical efficacy and adverse events related to cognitive enhancers are shown to be dose dependent. Our findings may help physicians choose targeted pharmaceuticals for patients with different stages and clinical symptoms.

In this network meta-analysis, we extend the previous meta-analysis by including a large number of studies with a broader dosage range and enrolling more patients in all stages of disease. Two dosages of galantamine 24 mg and 32 mg daily were involved for mild to moderate AD. The corresponding SMD (efficacy) of 0.01 and OR (tolerability) of 0.64 indicated no additional risk–benefit profile from higher doses. Hence, this evidence suggested that galantamine 24 mg daily probably had a favorable balance between benefits and tolerability. For patients with moderate to severe AD, it was observed that the combination therapy of donepezil 10 mg with memantine 20 mg offered the highest level of cognitive benefits, and the results generally supported previous studies and current guidelines [10, 25–27]. Moreover, we evaluated the effects and safety of donepezil 23 mg approved recently. This treatment showed beneficial effects on cognition, but had the lowest safety profile. The Asia-Pacific Expert Panel for donepezil 23 mg recommended that we should perform a stepwise escalation of donepezil to 23 mg daily and monitor the incidence of adverse events in a timely manner [28].

All pharmacological interventions are well tolerated for most patients. Compared with placebo, more side effects occurred with ChEIs and combined treatment, but not with memantine. Although ChEIs share a similar mode of action, they differ in pharmacologic characteristics and routes of administration, which can determine the pharmacological and toxicity profile [29]. Rivastigmine capsules 12 mg daily were strongly associated with high incidence of all-cause adverse events among ChEIs. The transdermal patch formulation approved for use across all stages of AD has been shown to have a better tolerability in comparison to the oral form. A higher dose of the rivastigmine transdermal patch (15 cm^2^) is also approved for the treatment of moderate to severe AD [30]. Better outcomes were observed as improvements in daily activities and global clinical impression in our analysis. The transdermal system delivery is innovative because it reaches the point of steady-state plasma concentration in a shorter time and alleviates the adverse reactions of the gastrointestinal tract better than the oral form based on the principle of pharmacokinetics [31]. Overall, the transdermal patch may provide a new approach to AD therapy.

Neuropsychiatric symptoms are common features throughout all stages of AD, which lead to a heavy burden for patients and caregivers [32]. Previous meta-analyses have already concluded that the ChEIs and memantine have benefits in the treatment with neuropsychiatric symptoms, and various drugs show mixed results [12, 33]. The results of our pairwise meta-analyses showed that only galantamine’s beneficial effects were verified compared with placebo, but no significant difference for cognitive enhancers was found through the network meta-analysis. Furthermore, there was no statistical difference between five active treatments and placebo in our subgroup meta-analysis. Fewer patients with neuropsychiatric symptoms based on our inclusion criteria may provide a reasonable explanation for the discrepant result. Many systematic reviews and meta-analyses recommended that antipsychotics, ginkgo biloba, and nonpharmacological treatment provide evidence of effects for patients with behavioral symptoms [12, 34–36]. Developing new drugs targeting neuropsychiatric symptoms and further research to strengthen evidence of therapeutic benefits are needed.

This network meta-analysis has several limitations. We found that the main inconsistency in our network analysis was in the loop of donepezil 5 mg–placebo–donepezil 10 mg, and we considered that this inconsistency was related to the difference in durations of the included trials. We conducted sensitivity network meta-analyses restricting the study period to 20–30 weeks, which did not result in any further inconsistency. The global heterogeneity of network meta-analyses was high in cognition among the population with moderate to severe AD, probably because different measurements (MMSE and SIB scale) were included in this network. In the pairwise meta-analysis, the heterogeneity was high for donepezil 10 mg vs placebo, and we found that two trials had a relatively shorter follow-up (12 weeks), which may have led to increased heterogeneity. The research implications of these results were limited by study characteristics (such as trial durations, simple sizes, and inclusion or exclusion criteria) and methodological models, and they also suffered from the bias due to quite fewer studies in a pair of comparisons or selective reporting, which might result in potential confounding factors to be cautious of. Although we used multiple databases to search published articles as much as we can and sent emails to authors for additional detailed information, we are still unable to exclude some possibilities that several unpublished studies are unavailable or that included eligible trials might overvalue the research outcomes. Furthermore, this network meta-analysis offers a clearly hierarchic relationship, but intervention rankings have a certain degree of imprecision, because most interventions have overlapping 95% CrIs [37].

In this network meta-analysis, we did not involve a cost-effectiveness analysis. Medication cost accounts for a large proportion of the total dementia healthcare cost. It is indicated by most economic evaluations that pharmacological treatments for AD are reasonable in terms of clinical effects and costs. The probabilistic sensitivity analyses suggested that donepezil and memantine were cost-effective with slightly greater quality-adjusted life years (QALYs) [38, 39]. Treatment with donepezil plus memantine was cost-effective within the willingness-to-pay threshold in moderate-to-severe AD [40, 41]. Nevertheless, the results generally are associated with a degree of uncertainty, which is related to country-specific data. In England, the use of prescription drugs doubled after introducing the national dementia strategies [42]. The proportion of people who receive the concomitant use of AChEI and memantine has increased in Europe [43]. Indeed, drug costs are very high in some countries (China, Indonesia, South Africa) because some drugs remain on patent and these countries are reluctant to use generic medicines.

## Conclusion

Our analysis provides some evidence that galantamine and donepezil may show the highest level of efficacy in cognition for mild to moderate AD, and the combination therapy (memantine 20 mg with donepezil 10 mg) and donepezil 23 mg daily are recommended for moderate to severe patients. The higher-dose rivastigmine transdermal patch (15 cm^2^) is the best option considering the benefits of both function and clinical global impression. None of the medicines is likely to improve behavior through current network meta-analysis. Memantine shows the best profile of acceptability, while rivastigmine oral form is associated with a high incidence of adverse events. Although the clinical effects are uncertain in the multifactor environment, these findings are helpful for guiding treatment decisions. Hopefully, further research should try to differentiate more clearly the effects of monotherapy versus combined therapies.

## Additional file

Additional file 1: **Supplementary 1.** Network meta-analysis model. **Supplementary 2.** References for included trials. **Supplementary 3.** Summary of included trials and patient characteristics. **Supplementary 4.** Risk of bias assessment. **Supplementary 5.** Results from pairwise meta-analysis for each outcome. **Supplementary 6.** Results from network meta-analysis for each outcome. **Supplementary 7.** Network plot for each outcome. **Supplementary 8.** Treatment ranking and rankograms for each outcome. **Supplementary 9.** Assessment of inconsistency results for each outcome. **Supplementary 10.** Comparison-adjusted funnel plot for each outcome from the network meta-analysis. **Supplementary 11.** Subgroup network meta-analysis for secondary outcomes. **Supplementary 12.** Sensitivity network meta-analysis for primary outcomes. **Supplementary 13.** Assessment of heterogeneity results for each outcome. (PDF 2427 kb)

## Abbreviations

AD: Alzheimer’s disease
ADAS-cog: Alzheimer’s Disease Assessment Scale—cognition subscale
ADCS-ADL: Alzheimer’s Disease Cooperative Study—Activities of Daily Living
BADLS: Bristol Activities of Daily Living Scale
CGIC: Clinical Global Impression of Change
ChEI: Cholinesterase inhibitor
CI: Confidence interval
CIBIC plus: Clinician’s Interview Based Impression of Change Plus Caregiver Input
CrI: Credible interval
DSM: Diagnostic and Statistical Manual of Mental Disorders
FDA: Food and Drug Administration
ITT: Intention to treat
MMSE: Mini-Mental State Examination
NINCDS-ADRDA: National Institute of Neurological and Communicative Disorders and Stroke and the Alzheimer's Disease and Relate Disorders Association
NPI: Neuropsychiatric Inventory
OR: Odds ratio
RCT: Randomized controlled trial
SIB: Severe Impairment Battery
SMD: Standardized mean difference
SUCRA: Surface under the cumulative ranking curve
