# Supplementary material for: Comparative safety and effectiveness of cholinesterase inhibitors and memantine for Alzheimer’s disease: a network meta-analysis of 41 randomized controlled trials
Source: Alzheimers Res Ther. 2018 Dec 27;10:126. doi: 10.1186/s13195-018-0457-9 (PMC6309083; doi:10.1186/s13195-018-0457-9)
Supplement: Supplementary file 1 — Supplementary 1. Network meta-analysis model. Supplementary 2. References for included trials. Supplementary 3. Summary of included trials and patient characteristics. Supplementary 4. Risk of bias assessment. Supplementary 5. Results from pairwise meta-analysis for each outcome. Supplementary 6. Results from network meta-analysis for each outcome. Supplementary 7. Network plot for each outcome. Supplementary 8. Treatment ranking and rankograms for each outcome. Supplementary 9. Assessment of inconsistency results for each outcome. Supplementary 10. Comparison-adjusted funnel plot for each outcome from the network meta-analysis. Supplementary 11. Subgroup network meta-analysis for secondary outcomes. Supplementary 12. Sensitivity network meta-analysis for primary outcomes. Supplementary 13. Assessment of heterogeneity results for each outcome. (PDF 2427 kb) [file 13195_2018_457_MOESM1_ESM.pdf]

Contents of Supplementary

Supplementary 1	Network meta-analysis model	Page 1
Supplementary 2	References for included trials	Page 5
Supplementary 3	Summary of included trials and patients characteristics	Page 9
Supplementary 4	Risk of bias assessment	Page 14
Supplementary 5	Results from pair-wise meta-analysis for each outcome	Page 17
Supplementary 6	Results from network meta-analysis for each outcome	Page 24
Supplementary 7	Network plot for each outcome	Page 29
Supplementary 8	Treatment ranking and rankograms for each outcome	Page 32
Supplementary 9	Assessment of inconsistency results for each outcome	Page 38
Supplementary 10	Comparison-adjusted funnel plot for each outcome from the network meta-analysis	Page 47
Supplementary 11	Subgroup network meta-analysis for secondary outcomes	Page 54
Supplementary 12	Sensitivity network meta-analysis for primary outcomes	Page 58
Supplementary 13	Assessment of heterogeneity results for each outcome	Page 61

Supplementary 1:
Network meta-analysis model

1. Random Effects Model for Continuous Data in WinBUGS

```
model {
        for(i in 1:ns) {
                w[i,1]<- 0
              delta[i,t[i,1]]<- 0

                ss[i]<- sum(n[i,1:na[i]])
                nom[i]<- sum(nom1[i,1:na[i]])
                pooled.sd[i]<- sqrt(nom[i]/(ss[i]-na[i]))
                J[i]<- 1-(3/((4*(ss[i]-na[i]))-1))

#Normal Likelihood#

                for (k in 1:na[i]) {
                        y[i,k] ~ dnorm(phi[i,t[i,k]],prec[i,k])

                        se[i,k]<- sd[i,k]/sqrt(n[i,k])
                        var[i,k]<- se[i,k]*se[i,k]
                        prec[i,k]<- 1/var[i,k]
                        nom1[i,k]<- (n[i,k]-1)*sd[i,k]*sd[i,k]
                }

#Parameterization of the model#

                phi[i,t[i,1]]<- u[i]*(pooled.sd[i]/J[i])

                for (k in 2:na[i]) {
                        phi[i,t[i,k]]<- (u[i]+delta[i,t[i,k]])*(pooled.sd[i]/J[i])
                        delta[i,t[i,k]] ~ dnorm(md[i,t[i,k]],taud[i,t[i,k]])
                        md[i,t[i,k]]<- d[t[i,k]] - d[t[i,1]]    + sw[i,k]
                        taud[i,t[i,k]]<- tau *2*(k-1)/k
                        w[i,k]<- (delta[i,t[i,k]] - d[t[i,k]] + d[t[i,1]])
                        sw[i,k]<- sum(w[i,1:k-1])/(k-1)
                }
        }

#Priors#

        SD ~ dnorm(0,1)I(0,1)
        tau<- 1/pow(SD,2)

        for(k in 1:(ref-1)) {
```

```
		d[k] ~ dnorm(0,.0001)
	}
	for(k in (ref+1):nt) {
		d[k] ~ dnorm(0,.0001)
	}
	for(i in 1:ns) {
	    u[i] ~ dnorm(0,.0001)
	}

#Estimated & Predicted Standardized Mean Differences#

	d[ref]<- 0

	for (c in 1:(ref-1)) {
		SMD.ref[c]<- d[c] - d[ref]
		predSMD.ref[c] ~ dnorm( SMD.ref[c],tau)
	}
	for (c in (ref+1):nt) {
		SMD.ref[c]<- d[c] - d[ref]
		predSMD.ref[c] ~ dnorm( SMD.ref[c],tau)
	}
	for (c in 1:(nt-1)) {
		for (k in (c+1):nt) {
			SMD[c,k]<- d[k] - d[c]
			predSMD[c,k] ~ dnorm(SMD[c,k],tau)
		}
	}

#Ranking of treatments#

	for(k in 1:nt) {
		order[k]<-nt+1-rank(d[],k)
# this is when the outcome is positive - omit    'nt+1-' when the outcome is negative
		most.effective[k]<-equals(order[k],1)

		for(j in 1:nt) {
			effectiveness[k,j]<- equals(order[k],j)
		}
	}
	for(k in 1:nt) {
		for(j in 1:nt) {
			cumeffectiveness[k,j]<- sum(effectiveness[k,1:j])
		}
	}
```

```
#SUCRAS#

        for(k in 1:nt) {
                SUCRA[k]<- sum(cumeffectiveness[k,1:(nt-1)]) /(nt-1)
        }

#Fit of the Model#

        for(i in 1:ns) {
                for(k in 1:na[i]) {
                        Darm[i,k]<-(y[i,k]-phi[i,t[i,k]])*(y[i,k]-phi[i,t[i,k]])/var[i,k]
                }
                D[i]<- sum(Darm[i,1:na[i]])
        }
        D.bar<- sum(D[])
}‘
```

2. Random Effects Model for Dichotomous Data in WinBUGS

```
# Binomial likelihood, logit link
# Random effects model for multi-arm trials
model{                                                  # *** PROGRAM STARTS
for(i in 1:ns){                                 # LOOP THROUGH STUDIES
        w[i,1] <- 0       # adjustment for multi-arm trials is zero for control arm
        delta[i,1] <- 0                       # treatment effect is zero for control arm
        mu[i] ~ dnorm(0,.0001)                      # vague priors for all trial baselines
        for (k in 1:na[i]) {                       # LOOP THROUGH ARMS
                r[i,k] ~ dbin(p[i,k],n[i,k]) # binomial likelihood
                logit(p[i,k]) <- mu[i] + delta[i,k]    # model for linear predictor
                rhat[i,k] <- p[i,k] * n[i,k] # expected value of the numerators
#Deviance contribution
                dev[i,k] <- 2 * (r[i,k] * (log(r[i,k])-log(rhat[i,k]))
                        +    (n[i,k]-r[i,k]) * (log(n[i,k]-r[i,k]) - log(n[i,k]-rhat[i,k])))                }
#    summed residual deviance contribution for this trial
        resdev[i] <- sum(dev[i,1:na[i]])
        for (k in 2:na[i]) {                       # LOOP THROUGH ARMS
# trial-specific LOR distributions
                delta[i,k] ~ dnorm(md[i,k],taud[i,k])
# mean of LOR distributions (with multi-arm trial correction)
                md[i,k] <-    d[t[i,k]] - d[t[i,1]] + sw[i,k]
# precision of LOR distributions (with multi-arm trial correction)
                taud[i,k] <- tau *2*(k-1)/k
# adjustment for multi-arm RCTs
                w[i,k] <- (delta[i,k] - d[t[i,k]] + d[t[i,1]])
# cumulative adjustment for multi-arm trials
```

```
            sw[i,k] <- sum(w[i,1:k-1])/(k-1)
        }
    }
totresdev <- sum(resdev[])                    # Total Residual Deviance
d[1]<-0             # treatment effect is zero for reference treatment
# vague priors for treatment effects
for (k in 2:nt){    d[k] ~ dnorm(0,.0001) }
sd ~ dunif(0,5)          # vague prior for between-trial SD
tau <- pow(sd,-2)      # between-trial precision = (1/between-trial variance)
for (c in 1:(nt-1))
                  {    for (k in (c+1):nt)
                              {    lor[c,k] <- d[k] - d[c]
                                   log(or[c,k]) <- lor[c,k]
                              }
                   }
for(k in 1:nt) {
        order[k]<- nt+1-rank(d[],k)
# this is when the outcome is positive - omit    'nt+1-' when the outcome is negative
        most.effective[k]<-equals(order[k],1)

        for(j in 1:nt) {
            effectiveness[k,j]<- equals(order[k],j)
        }
    }
    for(k in 1:nt) {
        for(j in 1:nt) {
            cumeffectiveness[k,j]<- sum(effectiveness[k,1:j])
        }
    }

for(k in 1:nt) {
        cum[k]<- sum(effectiveness[k,1:4]) /4
    }

#SUCRAS#
    for(k in 1:nt) {
        SUCRA[k]<- sum(cumeffectiveness[k,1:(nt-1)]) /(nt-1)
    }
}                                                # *** PROGRAM ENDS
```

Supplementary 2:
References for included trials

[1] Rogers SL, Doody RS, Mohs RC, Friedhoff LT. Donepezil improves cognition and global function in Alzheimer disease: a 15-week, double-blind, placebo-controlled study. Donepezil Study Group. Archives of internal medicine 1998; 158(9): 1021-31.

[2] Rogers SL, Farlow MR, Doody RS, Mohs R, Friedhoff LT. A 24-week, double-blind, placebo-controlled trial of donepezil in patients with Alzheimer's disease. Donepezil Study Group. Neurology 1998; 50(1): 136-45.

[3] Burns A, Rossor M, Hecker J, Gauthier S, Petit H, Moller HJ, et al. The effects of donepezil in Alzheimer's disease - results from a multinational trial. Dementia and geriatric cognitive disorders 1999; 10(3): 237-44.

[4] Homma A, Takeda M, Imai Y, Udaka F, Hasegawa K, Kameyama M, et al. Clinical efficacy and safety of donepezil on cognitive and global function in patients with Alzheimer's disease. A 24-week, multicenter, double-blind, placebo-controlled study in Japan. E2020 Study Group. Dementia and geriatric cognitive disorders 2000; 11(6): 299-313.

[5] Homma A, Imai Y, Tago H, Asada T, Shigeta M, Iwamoto T, et al. Donepezil treatment of patients with severe Alzheimer's disease in a Japanese population: results from a 24-week, double-blind, placebo-controlled, randomized trial. Dementia and geriatric cognitive disorders 2008; 25(5): 399-407.

[6] Feldman H, Gauthier S, Hecker J, Vellas B, Subbiah P, Whalen E. A 24-week, randomized, double-blind study of donepezil in moderate to severe Alzheimer's disease. Neurology 2001; 57(4): 613-20.

[7] Johannsen P, Salmon E, Hampel H, Xu Y, Richardson S, Qvitzau S, et al. Assessing therapeutic efficacy in a progressive disease: a study of donepezil in Alzheimer's disease. CNS drugs 2006; 20(4): 311-25.

[8] Seltzer B, Zolnouni P, Nunez M, Goldman R, Kumar D, Ieni J, et al. Efficacy of donepezil in early-stage Alzheimer disease: a randomized placebo-controlled trial. Archives of neurology 2004; 61(12): 1852-6.

[9] Winblad B, Kilander L, Eriksson S, Minthon L, Batsman S, Wetterholm AL, et al. Donepezil in patients with severe Alzheimer's disease: double-blind, parallel-group, placebo-controlled study. Lancet (London, England) 2006; 367(9516): 1057-65.

[10] Black SE, Doody R, Li H, McRae T, Jambor KM, Xu Y, et al. Donepezil preserves cognition and global function in patients with severe Alzheimer disease. Neurology 2007; 69(5): 459-69.

[11] Maher-Edwards G, Dixon R, Hunter J, Gold M, Hopton G, Jacobs G, et al. SB-742457 and donepezil in Alzheimer disease: a randomized, placebo-controlled study. International journal of geriatric psychiatry 2011; 26(5): 536-44.

[12] Farlow MR, Salloway S, Tariot PN, Yardley J, Moline ML, Wang Q, et al. Effectiveness and tolerability of high-dose (23 mg/d) versus standard-dose (10 mg/d) donepezil in moderate to severe Alzheimer's disease: A 24-week, randomized, double-blind study. Clinical therapeutics 2010; 32(7): 1234-51.

[13] Homma A, Atarashi H, Kubota N, Nakai K, Takase T. Efficacy and Safety of Sustained Release Donepezil High Dose versus Immediate Release Donepezil Standard Dose in Japanese Patients with Severe Alzheimer's Disease: A Randomized, Double-Blind Trial.

Journal of Alzheimer's disease : JAD 2016; 52(1): 345-57.

[14] Howard RJ, Juszczak E, Ballard CG, Bentham P, Brown RG, Bullock R, et al. Donepezil for the treatment of agitation in Alzheimer's disease. The New England journal of medicine 2007; 357(14): 1382-92.

[15] Tariot PN, Solomon PR, Morris JC, Kershaw P, Lilienfeld S, Ding C. A 5-month, randomized, placebo-controlled trial of galantamine in AD. The Galantamine USA-10 Study Group. Neurology 2000; 54(12): 2269-76.

[16] Brodaty H, Corey-Bloom J, Potocnik FC, Truyen L, Gold M, Damaraju CR. Galantamine prolonged-release formulation in the treatment of mild to moderate Alzheimer's disease. Dementia and geriatric cognitive disorders 2005; 20(2-3): 120-32.

[17] Burns A, Bernabei R, Bullock R, Cruz Jentoft AJ, Frolich L, Hock C, et al. Safety and efficacy of galantamine (Reminyl) in severe Alzheimer's disease (the SERAD study): a randomised, placebo-controlled, double-blind trial. The Lancet Neurology 2009; 8(1): 39-47.

[18] Raskind MA, Peskind ER, Wessel T, Yuan W. Galantamine in AD: A 6-month randomized, placebo-controlled trial with a 6-month extension. The Galantamine USA-1 Study Group. Neurology 2000; 54(12): 2261-8.

[19] Wilcock GK, Lilienfeld S, Gaens E. Efficacy and safety of galantamine in patients with mild to moderate Alzheimer's disease: multicentre randomised controlled trial. Galantamine International-1 Study Group. BMJ (Clinical research ed) 2000; 321(7274): 1445-9.

[20] Hager K, Baseman AS, Nye JS, Brashear HR, Han J, Sano M, et al. Effects of galantamine in a 2-year, randomized, placebo-controlled study in Alzheimer's disease. Neuropsychiatric disease and treatment 2014; 10: 391-401.

[21] Rockwood K, Fay S, Song X, MacKnight C, Gorman M. Attainment of treatment goals by people with Alzheimer's disease receiving galantamine: a randomized controlled trial. CMAJ : Canadian Medical Association journal = journal de l'Association medicale canadienne 2006; 174(8): 1099-105.

[22] Wilkinson D, Murray J. Galantamine: a randomized, double-blind, dose comparison in patients with Alzheimer's disease. International journal of geriatric psychiatry 2001; 16(9): 852-7.

[23] Rosler M, Anand R, Cicin-Sain A, Gauthier S, Agid Y, Dal-Bianco P, et al. Efficacy and safety of rivastigmine in patients with Alzheimer's disease: international randomised controlled trial. BMJ (Clinical research ed) 1999; 318(7184): 633-8.

[24] Feldman HH, Lane R. Rivastigmine: a placebo controlled trial of twice daily and three times daily regimens in patients with Alzheimer's disease. Journal of neurology, neurosurgery, and psychiatry 2007; 78(10): 1056-63.

[25] Winblad B, Cummings J, Andreasen N, Grossberg G, Onofrj M, Sadowsky C, et al. A six-month double-blind, randomized, placebo-controlled study of a transdermal patch in Alzheimer's disease--rivastigmine patch versus capsule. International journal of geriatric psychiatry 2007; 22(5): 456-67.

[26] Farlow MR, Grossberg GT, Sadowsky CH, Meng X, Somogyi M. A 24-week, randomized, controlled trial of rivastigmine patch 13.3 mg/24 h versus 4.6 mg/24 h in severe Alzheimer's dementia. CNS neuroscience & therapeutics 2013; 19(10): 745-52.

[27] Cummings J, Froelich L, Black SE, Bakchine S, Bellelli G, Molinuevo JL, et al. Randomized, double-blind, parallel-group, 48-week study for efficacy and safety of a higher-dose

rivastigmine patch (15 vs. 10 cm(2)) in Alzheimer's disease. Dementia and geriatric cognitive disorders 2012; 33(5): 341-53.

[28] Nakamura Y, Imai Y, Shigeta M, Graf A, Shirahase T, Kim H, et al. A 24-week, randomized, double-blind, placebo-controlled study to evaluate the efficacy, safety and tolerability of the rivastigmine patch in Japanese patients with Alzheimer's disease. Dementia and geriatric cognitive disorders extra 2011; 1(1): 163-79.

[29] Zhang ZX, Hong Z, Wang YP, He L, Wang N, Zhao ZX, et al. Rivastigmine Patch in Chinese Patients with Probable Alzheimer's disease: A 24-week, Randomized, Double-Blind Parallel-Group Study Comparing Rivastigmine Patch (9.5 mg/24 h) with Capsule (6 mg Twice Daily). CNS neuroscience & therapeutics 2016; 22(6): 488-96.

[30] Karaman Y, Erdogan F, Koseoglu E, Turan T, Ersoy AO. A 12-month study of the efficacy of rivastigmine in patients with advanced moderate Alzheimer's disease. Dementia and geriatric cognitive disorders 2005; 19(1): 51-6.

[31] Reisberg B, Doody R, Stoffler A, Schmitt F, Ferris S, Mobius HJ. Memantine in moderate-to-severe Alzheimer's disease. The New England journal of medicine 2003; 348(14): 1333-41.

[32] Peskind ER, Potkin SG, Pomara N, Ott BR, Graham SM, Olin JT, et al. Memantine treatment in mild to moderate Alzheimer disease: a 24-week randomized, controlled trial. The American journal of geriatric psychiatry : official journal of the American Association for Geriatric Psychiatry 2006; 14(8): 704-15.

[33] van Dyck CH, Tariot PN, Meyers B, Malca Resnick E. A 24-week randomized, controlled trial of memantine in patients with moderate-to-severe Alzheimer disease. Alzheimer disease and associated disorders 2007; 21(2): 136-43.

[34] Bakchine S, Loft H. Memantine treatment in patients with mild to moderate Alzheimer's disease: results of a randomised, double-blind, placebo-controlled 6-month study. Journal of Alzheimer's disease : JAD 2008; 13(1): 97-107.

[35] Herrmann N, Gauthier S, Boneva N, Lemming OM. A randomized, double-blind, placebo-controlled trial of memantine in a behaviorally enriched sample of patients with moderate-to-severe Alzheimer's disease. International psychogeriatrics 2013; 25(6): 919-27.

[36] Wang T, Huang Q, Reiman EM, Chen K, Li X, Li G, et al. Effects of memantine on clinical ratings, fluorodeoxyglucose positron emission tomography measurements, and cerebrospinal fluid assays in patients with moderate to severe Alzheimer dementia: a 24-week, randomized, clinical trial. Journal of clinical psychopharmacology 2013; 33(5): 636-42.

[37] Howard R, McShane R, Lindesay J, Ritchie C, Baldwin A, Barber R, et al. Donepezil and memantine for moderate-to-severe Alzheimer's disease. The New England journal of medicine 2012; 366(10): 893-903.

[38] Tariot PN, Farlow MR, Grossberg GT, Graham SM, McDonald S, Gergel I. Memantine treatment in patients with moderate to severe Alzheimer disease already receiving donepezil: a randomized controlled trial. Jama 2004; 291(3): 317-24.

[39] Shao ZQ. Comparison of the efficacy of four cholinesterase inhibitors in combination with memantine for the treatment of Alzheimer's disease. International journal of clinical and experimental medicine 2015; 8(2): 2944-8.

[40] Zhang N, Wei C, Du H, Shi FD, Cheng Y. The Effect of Memantine on Cognitive Function and Behavioral and Psychological Symptoms in Mild-to-Moderate Alzheimer's Disease

Patients. Dementia and geriatric cognitive disorders 2015; 40(1-2): 85-93.
[41] Bullock R, Touchon J, Bergman H, Gambina G, He Y, Rapatz G, et al. Rivastigmine and donepezil treatment in moderate to moderately-severe Alzheimer's disease over a 2-year period. Current medical research and opinion 2005; 21(8): 1317-27.

Supplementary 3:
Summary of included trials and patients characteristics

Study	Criteria	Patients (n)	Duration (weeks)	Age (SD)	Gender (%female)	Race (%white)	Disease severity	Baseline MMSE (SD)
**Donepezil**								
Rogers et al	DSM-III-R	Donepezil 5 mg (157)	12	73·8 (8·4)	69	95	Mild to moderately severe	19·4 (4·9)
1998a	NINCDS-ADRDA	Donepezil 10 mg (158)		73·4 (8·2)	61	96		19·4 (5·0)
		Placebo (153)		74·0 (8·0)	61	96		19·8 (4·3)
Rogers et al	DSM-III-R	Donepezil 5 mg (154)	24	72·9 (7·5)	63	95	Mild to moderate	19·0 (5·0)
1998b	NINCDS-ADRDA	Donepezil 10 mg (157)		74·6 (7·5)	62	96		18·9 (5·0)
		Placebo (162)		72·6 (7·6)	61	94		19·2 (5·1)
Burns et al	DSM-III-R	Donepezil 5 mg (271)	24	72·0 (8·2)	61	100	Mild to moderately severe	20 (4·9)
1999	NINCDS-ADRDA	Donepezil 10 mg (273)		72·0 (8·3)	57	99		20 (3·3)
		Placebo (274)		71·0 (8·3)	55	99		20 (5·0)
Homma et al	DSM-IV	Donepezil 5 mg (116)	24	70·1 (7·6)	68	Japanese	Mild to moderately severe	17·8 (3·9)
2000		Placebo (112)		69·4 (8·8)	66			16·6 (3·9)
Homma et al	DSM-IV	Donepezil 5 mg (96)	24	78·0 (8·9)	79·2	Japanese	Severe	7·9 (3·3)
2008		Donepezil 10 mg (92)		76·9 (7·9)	79·3			7·4 (3·4)
		Placebo (102)		79·7 (7·5)	82·4			8·0 (3·3)
Feldman et al	NINCDS-ADRDA	Donepezil 10 mg (144)	24	73·3	61·1	…	Moderate to severe	11·7 (4·2)
2001		Placebo (146)		74	61	…		12·0 (4·1)
Johannsen et al	DSM-IV	Donepezil 10 mg (99)	12	74·1 (7·6)	59·6	…	Mild to moderate	18·8 (4·8)
2006	NINCDS-ADRDA	Placebo (103)		71·4 (9·3)	63·1	…		18·5 (4·8)
Seltzer et al	DSM-IV	Donepezil 10 mg (96)	24	73·3 (9·6)	50	…	Mild to moderate	24·1 (1·7)
2004	NINCDS-ADRDA	Placebo (57)		75·1 (8·8)	60	…		24·3 (1·3)

Winblad et al 2006	DSM-IV	Donepezil 10 mg (128)	24	84·5 (6·0)	79	100	Severe	6·0 (3·0)
	NINCDS-ADRDA	Placebo (120)		85·3 (5·9)	74	99		6·2 (3·0)
Black et al 2007	DSM-IV	Donepezil 10 mg (176)	24	78·0 (8·0)	72·7	76·1	Severe	7·5 (3·3)
	NINCDS-ADRDA	Placebo (167)		78·0 (8·2)	67·7	76		7·4 (3·6)
Maher-Edwards et al ; 2011	DSM-IV	Donepezil 10 mg (67)	24	71·1 (8·4)	63	…	Mild to moderate	19·2 (3·2)
	NINCDS-ADRDA	Placebo (61)		71·6 (6·7)	70	…		18·3 (3·4)
Farlow et al 2010	DSM-IV	Donepezil 23 mg (963)	24	73·9 (8·5)	63	73·5	Moderate to severe	13·1 (5·0)
	NINCDS-ADRDA	Donepezil 10 mg (471)		73·8 (8·6)	62·4	73·5		13·1 (4·7)
Homma et al 2016	MMSE	Donepezil 10 mg (166)	24	76·2 (8·8)	69·6	Japanese	Severe	8·9 (3·1)
		Donepezil 23 mg (185）		75·7 (8·8)	69·3			8·5 (3·2)
Howard et al 2007	NINCDS-ADRDA	Donepezil 10 mg (128)	12	84·9 (7·3)	82	98·4	Severe	8·1 (5·9)
		Placebo (131)		84·4 (8·2)	87	96·2		8·2 (6·8)
**Galantamine**								
Tariot et al 2000	NINCDS-ADRDA	Galantamine 24 mg (273)	21	77·7 (6·6)	67	91	Mild to moderate	17·7 (3·3)
		Placebo(286)		77·1 (8·5)	62·2	93		17·7 (3·4)
Brodaty et al 2005	NINCDS-ADRDA	Galantamine 24 mg (327)	26	76·5 (7·8)	64	90	Mild to moderate	17·8 (4·1)
		Placebo (324)		76·3 (8·0)	64	90		18·1 (4·1)
Burns et al 2009	DSM-IV	Galantamine 24 mg (207)	26	83·7 (5·7)	81	…	Severe	8·8 (2·4)
	NINCDS-ADRDA	Placebo (200)		83·5 (5·8)	81	…		9·1 (2·4)
Raskind et al 2000	NINCDS-ADRDA	Galantamine 24 mg (212)	24	75·9 (7·3)	65·6	92	Mild to moderate	19·5 (4·4)
		Galantamine 32 mg (211)		75·0 (8·7)	58·8	90		19·1 (4·4)
		Placebo (213)		75·3 (8·8)	61·5	92		19·2 (4·4)
Wilcock et al 2000	NINCDS-ADRDA	Galantamine 24 mg (220)	24	71·9 (8·3)	63·2	…	Mild to moderate	19·5 (3·4)
		Galantamine 32 mg (218)		72·1 (8·6)	63·3	…		19·0 (3·8)
		Placebo (215)		72·7 (7·6)	61·4	…		19·3 (3·5)

Hager et al	NINCDS-ADRDA	Galantamine 24 mg (1024)	104	73·0 (8·9)	65·5	100	Mild to moderately severe	19·0 (4·2)
2014		Placebo (1021)		73·0 (8·7)	64·1	99·7		19·0 (4·0)
Rockwood et al	NINCDS-ADRDA	Galantamine 24 mg (64)	16	77·0 (8·0)	64	…	Mild to moderate	20·8 (3·3)
2006		Placebo (66)		78·0 (8·0)	62	…		19·9 (4·2)
Wilkinson et al	DSM-III-R	Galantamine 24 mg (56)	12	72·9 (8·2)	59	…	Mild to moderate	18·2 (3·0)
2001	NINCDS-ADRDA	Placebo (87)		74·2 (8·4)	59	…		18·7 (2·8)
**Rivastigmine**								
Rösler et al	DSM-IV	Rivastigmine 12 mg (243)	26	72	59	97	Mild to moderately severe	19·9
1999	NINCDS-ADRDA	Placebo (239)						
Feldman et al	DSM-IV	Rivastigmine 12 mg (227)	26	71·4 (7·9)	60	…	Mild to moderate	18·3 (4·5)
2007a	NINCDS-ADRDA	Placebo (222)		71·7 (8·7)	60	…		18·7 (4·6)
Winblad et al	DSM-IV	Patch 10 cm^2(293)	24	73·6 (7·9)	68	75·6	Mild to moderate	16·6 (3·1)
2007	NINCDS-ADRDA	Rivastigmine 12 mg (297)		72·8 (8·2)	65·6	74·5		16·4 (3·1)
		Placebo (302)		73·9 (7·3)	66·6	75·2		16·4 (3·0)
Farlow et al	NINCDS-ADRDA	Patch 15 cm^2(356)	24	77·6 (8·7)	63·8	86	Severe	8·8 (2·9)
2013		Patch 5 cm^2(360)		76·5 (9·4)	65	88·6		8·8 (3·0)
Cummings et al	DSM-IV	Patch 10 cm^2(287)	48	75·9 (6·8)	63·4	98·3	Mild to moderate	14·2 (4·6)
2012	NINCDS-ADRDA	Patch 15 cm^2(280)		75·6 (7·4)	66·1	95		14·1 (4·8)
Nakamura et al	DSM-IV	Patch 5 cm^2(282)	24	74·3 (7·5)	68·8	…	Mild to moderate	16·8 (2·9)
2011	NINCDS-ADRDA	Patch 10 cm^2(287)		75·1 (6·9)	67·9	…		16·5 (3·1)
		Placebo (286)		74·5 (7·4)	68·2	…		16·6 (2·9)
Zhang et al	NINCDS-ADRDA	Patch 10 cm^2(248)	24	70·4 (8·0)	56·5	Chinese	Mild to moderate	16·0 (3·5)
2016		Rivastigmine 12 mg (253)		69·8 (8·2)	54·9			16·6(3·1)
Karaman et al	DSM-IV	Rivastigmine 12 mg (24)	52	74·1 (4·3)	54·2	…	Advanced moderate	11·4 (1·0)
2005	NINCDS-ADRDA	Placebo (20)		73·4 (4·0)	55	…		13·2 (0·9)

**Memantine**								
Reisberg et al	DSM-IV	Memantine 20 mg (126)	28	75·5 (8·2)	72·2	87·6	Moderate to severe	7·8 (3·8)
2003	NINCDS-ADRDA	Placebo (126)		75·8 (7·3)	65·5	89·3		8·1 (3·6)
Peskind et al	NINCDS–ADRDA	Memantine 20 mg (201)	24	78 (7·3)	60·2	92	Mild to moderate	17·4 (3·7)
2006		Placebo (202)		77 (8·2)	57·4	90·6		17·2 (3·4)
van Dyck et al	NINCDS–ADRDA	Memantine 20 mg (178)	24	78·1 (8·2)	72·5	79·8	Moderate to severe	10·0 (2·8)
2007		Placebo (172)		78·3 (7·6)	70·3	82		10·3 (3·1)
Bakchine and	DSM-IV	Memantine 20 mg (318)	24	74·0 (7·4)	65	100	Mild to moderate	18·6 (3·3)
Loft et al,2008	NINCDS-ADRDA	Placebo (152)		73·3 (6·9)	60	100		18·9 (3·2)
Herrmann et al	NINCDS–ADRDA	Memantine 20 mg (182)	24	74·7 (7·9)	57·7	…	Moderate to severe	11·9 (3·1)
2013		Placebo (187)		75·1 (6·9)	58·8	…		11·8 (2·9)
Wang et al	DSM-IV	Memantine 20 mg (11)	24	65·7 (12·5)	63·6	…	Moderate to severe	14·1 (4·6)
2013	NINCDS-ADRDA	Placebo (11)		64·7 (11·5)	63·6	…		10·1 (6·1)
**Donepezil+Memantine**								
Howard et al	NINCDS-ADRDA	Donepezil 10 mg +	52	77·5 (9·0)	67	92	Moderate to severe	9·1 (2·6)
2012		Memantine 20 mg (73)						
		Donepezil 10 mg (73)		77·2 (7·5)	70	95		9·0 (2·8)
		Memantine 20 mg (76)		76·2 (8·9)	61	96		9·2 (2·5)
		Placebo (73)		77·7 (8·0)	64	97		9·1 (2·4)
Tariot et al	NINCDS-ADRDA	Donepezil 10 mg (201)	24	75·5 (8·7)	67	92·5	Moderate to severe	10·2 (3·0)
2004		Donepezil 10 mg +		75·5 (8·5)	63	90·1		9·9 (3·1)
		Memantine 20 mg (202)						
Shao et al	DSM-IV	Memantine 20 mg (22)	24	73·0 (7·1)	50	…	Mild to moderate	15·3 (1·6)
2015		Donepezil 10 mg +		73·4 (6·0)	54·5			15·1 (1·8)
		Memantine 20 mg (22)						

**Donepezil vs memantine**								
Zhang et al	NINCDS-ADRDA	Memantine 20 mg (80)	24	69·8 (8·0)	61·3	…	Mild to moderate	15·9 (4·4)
2015		Donepezil 10 mg (87)		70·1 (8·0)	59·8	…		15·5 (4·2)
**Donepezil vs rivastigmine**								
Bullock et al	DSM-IV	Rivastigmine 12 mg (495)	104	75·9 (6·6)	68·9	98·6	Moderate to moderately	15·1 (3·0)
2005	NINCDS-ADRDA	Donepezil 10 mg (499)		75·8 (6·8)	68·5	99	severe	15·1 (2·9)

Full references for all trials are given in Appendix 2. DSM-IV = Diagnostic and Statistical Manual of Mental Disorders, Fourth Edition; DSM-ⅢI-R = Diagnostic and Statistical Manual of Mental Disorders third edition, revision; NINCDS-ADRDA= The National Institute of Neurological and Communicative Disorders Association and Stroke-AD and Related Disorders Association; MMSE=Mini-mental State Examination; …=Not available.

Supplementary 4:
Risk of bias assessment

Risk of bias summary: review authors' judgements about each risk of bias item for each included study.

	Random sequence generation (selection bias)	Allocation concealment (selection bias)	Blinding of participants and personnel (performance bias)	Blinding of outcome assessment (detection bias)	Incomplete outcome data (attrition bias)	Selective reporting (reporting bias)	Other bias
Bakchine and Loft 2008	−	−	?	?	+	?	?
Black 2007	+	?	+	+	+	?	?
Brodaty 2005	+	?	+	+	+	?	?
Bullock 2005	+	+	+	+	+	?	−
Burns 1999	?	?	?	?	+	+	?
Burns 2009	+	+	+	+	+	?	+
Cummings 2012	+	+	+	+	+	?	?
Farlow 2010	+	−	?	+	+	?	?
Farlow 2013	?	−	?	?	−	?	?
Feldman 2001	+	+	+	+	+	?	?
Feldman 2007	?	+	+	+	+	?	−
Hager 2014	+	?	+	+	+	?	?
Herrmann 2013	+	+	+	+	+	+	?
Homma 2000	?	?	?	?	+	?	?
Homma 2008	+	+	?	?	+	?	?
Homma 2016	+	?	?	?	−	?	?
Howard 2007	+	+	+	+	+	?	+
Howard 2012	+	+	?	+	+	+	?
Johannsen 2006	+	+	?	?	+	?	?

	Random sequence generation (selection bias)	Allocation concealment (selection bias)	Blinding of participants and personnel (performance bias)	Blinding of outcome assessment (detection bias)	Incomplete outcome data (attrition bias)	Selective reporting (reporting bias)	Other bias
Karaman 2005	−	+	?	?	−	?	?
Maher-Edwards 2011	+	?	?	−	+	?	?
Nakamura 2011	+	+	+	?	+	+	?
Peskind 2006	+	+	+	+	+	?	?
Raskind 2000	+	+	?	?	+	?	?
Reisberg 2003	+	?	?	?	+	?	?
Rockwood 2006	+	?	+	+	+	?	?
Rogers 1998a	+	?	?	+	+	?	?
Rogers 1998b	+	?	?	+	+	?	?
Rösler 1999	+	+	+	+	+	?	?
Seltzer 2004	−	−	?	?	+	?	?
Shao 2015	?	?	?	−	?	?	−
Tariot 2000	+	+	?	?	+	?	−
Tariot 2004	+	+	?	?	+	?	?
van Dyck 2007	?	+	?	?	+	?	−
Wang 2013	?	?	?	+	−	?	−
Wilcock 2000	+	+	?	?	+	−	?
Wilkinson 2001	+	+	?	?	+	−	?
Winblad 2006	+	+	+	+	+	?	?
Winblad 2007	+	?	?	?	+	−	?
Zhang 2015	?	−	?	−	+	?	?
Zhang 2016	+	+	+	?	+	?	?

Risk of bias graph: review authors' judgements about each risk of bias item presented as percentages across all included studies.

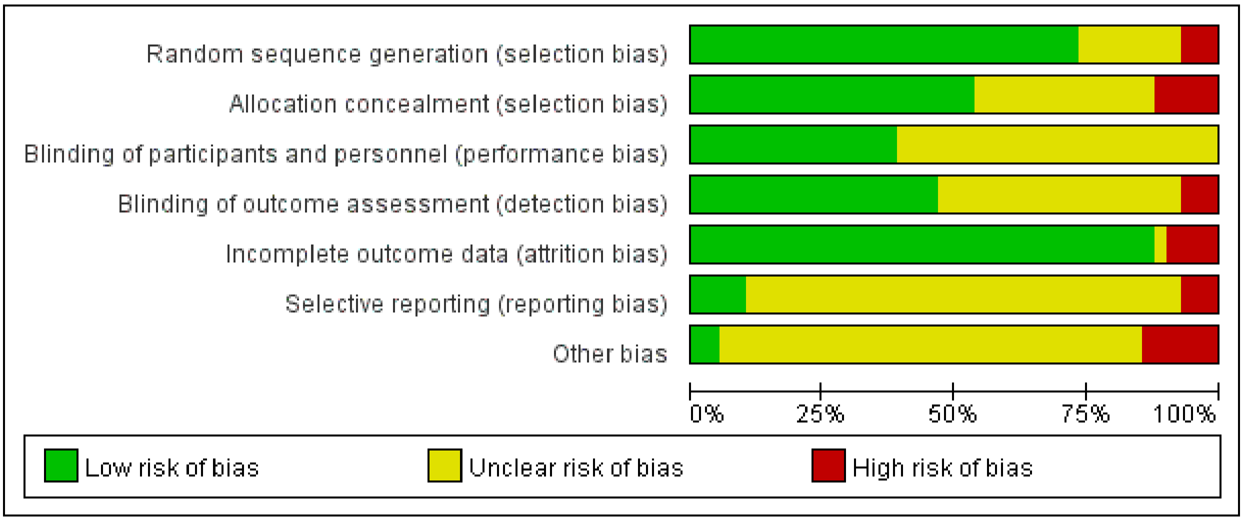

Supplementary 5:
Results from pair-wise meta-analysis for each outcome

Primary outcomes

Pair-wise meta-analysis for mild to moderate AD

Direct comparisons	Mean overall change of cognition SMD (95% CI)	All-cause adverse events OR (95% CI)
Donepezil 5 mg vs		
Donepezil 10 mg	0.165 (0.043 to 0.288)	0.593 (0.425 to 0.829)
Placebo	-0.380 (-0.500 to -0.259)	1.269 (0.851 to 1.893)
Donepezil 10 mg vs		
Placebo	-0.394(-0.560 to -0.227)	1.431 (1.052 to 1.947)
Galantamine 24 mg vs		
Galantanime 32 mg	-0.016 (-0.152 to 0.120)	0.705 (0.454 to 1.094)
Placebo	-0.511 (-0.596 to -0.426)	1.586 (1.277 to 2.050)
Galantamine 32 mg vs		
Placebo	-0.527 (-0.665 to -0.389)	2.773 (1.861 to 4.132)
Rivastigmine 12 mg vs		
Rivastigmine patch 10 cm ^{2}	-0.016 (-0.142 to 0.110)	1.602 (1.258 to 2.040)
Placebo	-0.296 (-0.409 to -0.182)	2.873 (1.839 to 4.488)
Rivastigmine patch 10 cm ^{2}vs		
Placebo	-0.240 (-0.360 to -0.119)	1.442(0.953 to 2.183)
Rivastigmine patch 15 cm ^{2}	0.098 (-0.072 to 0.268)	0.715(0.495 to 1.033)
Rivastigmine patch 5 cm ^{2}	-0.080 (-0.250 to 0.090)	1.021(0.633 to 1.646)
Rivastigmine patch 5 cm ^{2}vs		
Placebo	-0.160 (-0.330 to 0.011)	1.796(1.159 to 2.783)
Memantine 20 mg vs		
Donepezil 10 mg	-0.037 (-0.341 to 0.268)	NA
Plaecbo	-0.188 (-0.331 to -0.045)	1.018(0.761 to 1.361)

Pair-wise meta-analysis for moderate to severe AD

Direct comparisons	Mean overall change of cognition SMD (95% CI)	All-cause adverse events OR (95% CI)
Donepezil 10 mg vs		
Placebo	0.548 (0.374 to 0.721)	1.527 (1.139 to 2.047)
Donepezil 23 mg	-0.082 (-0.228 to 0.063)	0.608 (0.493 to 0.749)
Memantine 20 mg + Donepezil 10 mg	-0.231(-0.544 to 0.082)	0.721(0.458 to 1.136)
Memantine 20 mg	0.219 (-0.165 to 0.603)	NA
Memantine 20 mg vs		
Placebo	0.358 (0.049 to 0.668)	1.052(0.779 to 1.421)
Memantine 20mg + Donepezil 10mg	-0.265 (-0.643 to 0.113)	NA
Memantine 20mg + Donepezil 10mg vs		
Placebo	0.722 (0,341 to 1.103)	NA

Secondary outcomes

Pair-wise meta-analysis on function

Direct comparisons	Mean overall change of function SMD (95% CI)
Donepezil 10 mg vs	
Placebo	0.210 (0.074 to 0.345)
Memantine 20 mg	0.046 (-0.192 to 0.285)
Memantine 20 mg +Donepezil 10 mg	-0.169(-0.343 to 0.006)
Rivastigmine 12 mg	-0.107 (-0.236 to 0.022)
Memantine 20 mg vs	
Placebo	0.110 (-0.012 to 0.232)
Memantine 20 mg +Donepezil 10 mg	-0.107 (-0.462 to 0.248）
Memantine 20 mg +Donepezil 10 mg vs	
Placebo	0.510 (0.135 to 0.885)
Galantamine 24 mg vs	
Placebo	0.212 (0.105 to 0.320)
Rivastigmine 12 mg vs	
Placebo	0.076 (-0.301 to 0.453)
Rivastigmine patch 10 cm ^{2}	-0.031 (-0.157 to 0.094)

Rivastigmine patch 10 cm ²vs	
Placebo	0.238(0.066 to 0.409)
Rivastigmine patch 15 cm ²	-0.183 (-0.353 to -0.014)
Rivastigmine patch 15 cm ²vs	
Rivastigmine patch 5 cm ²	0.138 (-0.016 to -0.292)

Pair-wise meta-analysis on behavior

Direct comparisons	Mean overall change of behavior SMD (95% CI)
Donepezil 10 mg vs	
Placebo	-0.159 (-0.349 to 0.032)
Memantine 20 mg	0.107 (-0.110 to 0.325)
Rivastigmine 12 mg	0.031(-0.096 to 0.158)
Memantine 20 mg vs	-0.041(-0.179 to 0.096)
Placebo	
Galantamine 24 mg vs	
Placebo	-0.154 (-0.277 to -0.030)
Rivastigmine 12 mg vs	
Placebo	-0.039 (-0.209 to 0.131)
Rivastigmine 12 mg vs	
Rivastigmine patch 10 cm ²	-0.032 (-0.158 to 0.093）

Pair-wise meta-analysis on global changes

Direct comparisons	Response rate of global changes OR (95% CI)
Donepezil 5 mg vs	
Placebo	2.120 (1.432 to 3.138)
Donepezil 10 mg	0.783 (0.608 to 1.009)
Donepezil 10 mg vs	
Placebo	1.997 (1.623 to 2.457)
Donepezil 23 mg	1.166(0.639 to 2.129)
Galantamine 24 mg vs	
Placebo	1.236 (1.011 to 1.739)
Galantamine 32 mg	0.925 (0.469 to 1.825)

Galantamine 32 mg vs	
Placebo	1.428 (0.981 to 2.079)
Rivastigmine 12 mg vs	
Placebo	1.832 (1.399 to 2.399)
Rivastigmine patch 10 cm^2	1.070 (0.765 to 1.496)
Rivastigmine patch 10 cm^2 vs	
Rivastigmine patch 5 cm^2	1.040 (0.690 to 1.568)
Placebo	1.304 (0.980 to 1.735)
Rivastigmine patch 5 cm^2 vs	
Rivastigmine patch 15 cm^2	0.592 (0.399 to 0.879)
Placebo	1.482 (0.952 to 2.308)
Memanine 20 mg vs	
Placebo	1.157(0.719 to 1.862)
Memantine 20 mg + Donepezil 10 mg vs	
Placebo	1.343(0.750 to 2.407)

Heterogeneity test, I^2 and heterogeneity estimate

Efficacy on cognition for mild to moderate AD

Direct comparisons	NO. of studies	P-value	I^2	τ^2
Donepezil 5 mg vs Donepezil 10 mg	3	0.328	10.3%	0.0012
Donepezil 5 mg vs Placebo	4	0.289	20.2%	0.0031
Donepezil 10 mg vs Placebo	6	0.026	60.8%	0.0253
Galantamine 24 mg vs Galantamine 32 mg	2	0.318	0.0%	0.0000
Galantamine 24 mg vs Placebo	6	0.615	0.0%	0.0000
Galantamine 32 mg vs Placebo	2	0.894	0.0%	0.0000
Rivastigmine 12 mg vs Placebo	3	0.287	19.8%	0.0020
Rivastigmine 10 cm^2 vs Placebo	2	0.971	0.0%	0.0000
Memantine 20 mg vs Placebo	2	0.442	0.0%	0.0000
Rivastigmine 12 mg vs Rivastigmine patch 10 cm^2	2	0.799	0.0%	0.0000

All-cause adverse events for mild to moderate AD

Direct comparisons	NO. of studies	P-value	I^2	τ^2
Donepezil 5 mg vs Donepezil 10 mg	2	0.836	0.0%	0.0000
Donepezil 5 mg vs Placebo	3	0.109	54.8%	0.0683
Donepezil 10 mg vs Placebo	5	0.225	29.5%	0.0362
Galantamine 24 mg vs Galantamine 32 mg	2	0.313	1.8%	0.0020
Galantamine 24 mg vs Placebo	7	0.018	60.8%	0.0637
Galantamine 32 mg vs Placebo	2	0.484	0.0%	0.0000

Rivastigmine 12 mg vs Placebo	3	0.063	63.9%	0.0988
Rivastigmine 10 cm^2 vs Placebo	2	0.124	57.7%	0.0524
Memantine 20 mg vs Placebo	2	0.372	0.0%	0.0000
Rivastigmine 12 mg vs Rivastigmine patch 10 cm^2	2	0.651	0.0%	0.0000

Efficacy on cognition for moderate to severe AD

Direct comparisons	NO. of studies	P-value	I^2	τ^2
Donepezil 10 mg vs Donepezil 23 mg	2	0.2	39.1%	0.0049
Donepezil 10 mg vs Placebo	6	0.064	52%	0.0233
Donepezil 10 mg vs Memantine 20 mg + Donepezil 10 mg	2	0.126	57.3%	0.039
Memantine 20 mg vs Placebo	4	0.021	69.3%	0.0617

All-cause adverse events for moderate to severe AD

Direct comparisons	NO. of studies	P-value	I^2	τ^2
Donepezil 10 mg vs Donepezil 23 mg	2	0.612	0.0%	0.0000
Donepezil 10 mg vs Placebo	4	0.837	0.0%	0.0000
Donepezil 10 mg vs Memantine 20 mg + Donepezil 10 mg	2	0.684	0.0%	0.0000
Memantine 20 mg vs Placebo	3	0.714	0.0%	0.0000

Efficacy on function

Direct comparisons	NO. of studies	P-value	I^2	τ^2
Donepezil 10 mg vs Placebo	4	0.447	0.0%	0.0000
Donepezil 10 mg vs Memantine 20 mg	2	0.451	0.0%	0.0000
Donepezil 10 mg vs Memantine20 mg + Donepezil10 mg	2	0.548	0.0%	0.0000
Memantine 20 mg vs Placebo	5	0.253	25.2%	0.0049
Memantine 20 mg vs Memantine 20 mg + Donepezil10 mg	2	0.279	14.8%	0.0112
Galantamine 24 mg vs Placebo	3	0.461	0.0%	0.0000
Rivastigmine 12 mg vs Placebo	2	0.179	44.6%	0.0426
Rivastigmine 12 mg vs Rivastigmine patch 10 cm^2	2	0.849	0.0%	0.0000

Efficacy on behavior

Direct comparisons	NO. of studies	P-value	I^2	τ^2
Donepezil 10 mg vs Placebo	5	0.023	64.7%	0.0304
Memantine 20 mg vs Placebo	6	0.159	37.1%	0.0090
Galantamine 24 mg vs Placebo	2	0.839	0.0%	0.0000
Rivastigmine 12 mg vs Rivastigmine patch 10 cm^2	2	0.159	49.6%	0.0140

Efficacy on global changes

Direct comparisons	NO. of studies	P-value	I^2	τ^2
Donepezil 5 mg vs Placebo	5	0.037	60.7%	0.1208
Donepezil 5 mg vs Donepezil 10 mg	4	0.350	8.7%	0.0059
Donepezil 10 mg vs Placebo	7	0.450	0.0%	0.0000
Donepezil 10 mg vs Donepezil 23 mg	2	0.058	72.3%	0.1409
Galantamine 24 mg vs Placebo	5	0.176	36.8%	0.0346
Galantamine 24 mg vs Galantamine 32 mg	2	0.063	71.1%	0.1712
Galantamine 32 mg vs Placebo	2	0.381	0.0%	0.0000
Rivastigmine 12 mg vs Placebo	3	0.273	23.0%	0.0132
Rivastigmine patch 10 cm^2 vs Placebo	2	0.328	0.0%	0.0000
Rivastigmine 12 mg vs Rivastigmine patch 10 cm^2	2	0.203	38.4%	0.0224

Publication bias assessed by Egger's test

Efficacy on cognition for mild to moderate AD

Std_Eff	Coef.	Std. Err.	t	Pt	[95% Conf. Interval]	
slope	0.1787227	0.166841	1.07	0.292	-0.1603391	0.5177845
bias	0.9610271	1.637752	0.59	0.561	-2.367286	4.28934

All-cause adverse events for mild to moderate AD

Std_Eff	Coef.	Std. Err.	t	Pt	[95% Conf. Interval]	
slope	-0.0971094	0.2432059	-0.40	0.692	-0.593131	0.3989122
bias	1.568515	1.133214	1.38	0.176	-0.7426903	3.87972

Efficacy on cognition for moderate to severe AD

Std_Eff	Coef.	Std. Err.	t	Pt	[95% Conf. Interval]	
slope	0.0447009	0.1340018	0.33	0.743	-.2409172	0.330319
bias	2.066903	1.058127	1.95	0.070	-0.1884426	4.322248

All-cause adverse events for moderate to severe AD

Std_Eff	Coef.	Std. Err.	t	Pt	[95% Conf. Interval]	
slope	1.693871	0.3442733	4.92	0.001	0.8999752	2.487766
bias	-1.643118	1.516087	-1.08	0.310	-5.13922	1.852985

Efficacy on function

Std_Eff	Coef.	Std. Err.	t	Pt	[95% Conf. Interval]	
slope	0.0535554	0.0987051	0.54	0.593	-0.1523397	0.2594506
bias	0.2191142	1.019427	0.21	0.832	-1.907374	2.345602

Efficacy on behavior

Std_Eff	Coef.	Std. Err.	t	Pt	[95% Conf. Interval]	
slope	0.1495432	0.1390142	1.08	0.298	-0.1451537	0.4442401
bias	-2.131178	1.369578	-1.56	0.139	-5.034553	0.7721966

Efficacy on global changes

Std_Eff	Coef.	Std. Err.	t	Pt	[95% Conf. Interval]	
slope	0.0959715	0.3798246	0.25	0.802	-0.6743485	0.8662916
bias	-0.0390849	1.613679	-0.02	0.981	-3.311779	3.233609

Supplementary 6:
Results from network meta-analysis for each outcome

**DON 10 mg**	0.61 (0.43 to 0.83)	1.52 (0.90 to 2.42)	0.75 (0.40 to 1.28)	1.56 (1.09 to 2.17)
-0.06 (-0.39 to 0.27)	**DON 23 mg**	2.56 (1.35 to 4.50)	1.27 (0.62 to 2.34)	2.63 (1.59~4.14)
0.19 (-0.14 to 0.50)	0.25 (-0.22 to 0.70)	**MEM 20 mg**	0.53 (0.23 to 1.04)	1.06 (0.72 to 1.49)
-0.22 (-0.55 to 0.13)	-0.16 (-0.63 to 0.32)	-0.41 (-0.81 to 0.01)	**MEM 20 mg + DON 10 mg**	2.27 (1.10 to 4.19)
0.53 (0.33 to 0.75)	0.60 (0.21 to 0.99)	0.34 (0.08 to 0.63)	0.76 (0.39 to 1.11)	**PBO**

Treatment; Efficacy (SMD & 95% CrI); Tolerability (OR & 95 % CrI)

Comparative efficacy and tolerability for moderate to severe AD in the network meta-analysis.

Comparisons should be read from left to right. The efficacy and tolerability estimate is located at the intersection of the column-defining treatment and the row-defining treatment. For efficacy, an SMD above 0 favors the column-defining treatment. For tolerability, an OR below 1 favors the row-defining treatment. Significant results are in bold and underlined. PBO = placebo; DON = donepezil; GAL = galantamine; RIV = rivastigmine; MEM = memantine; SMD = standardized mean difference; OR = odds ratios; CrI = credible intervals.

Efficacy on function

NO.	Interventions
1	Placebo
2	Donepezil 10 mg
3	Galantamine 24 mg
4	Rivastigmine 12 mg
5	Rivastigmine patch 10cm ²
6	Rivastigmine patch 15cm ²
7	Rivastigmine patch 5cm ²
8	Memantine 20 mg
9	Memantine 20 mg +Donepezil 10 mg

node	SMD	sd	MC error	2.50%	median	97.50%	start	sample
SMD[1,2]	0.1485	0.05998	7.21E-04	0.03024	0.1488	0.2673	50001	300000
SMD[1,3]	0.2105	0.06478	5.76E-04	0.08127	0.21	0.3379	50001	300000
SMD[1,4]	0.2121	0.07268	7.72E-04	0.06431	0.214	0.3526	50001	300000
SMD[1,5]	0.2388	0.08596	9.13E-04	0.06725	0.24	0.4077	50001	300000
SMD[1,6]	0.4234	0.1374	0.001698	0.1479	0.4249	0.6895	50001	300000
SMD[1,7]	0.2836	0.1691	0.001974	-0.05428	0.2853	0.6136	50001	300000
SMD[1,8]	0.1151	0.05607	6.12E-04	0.006824	0.1138	0.228	50001	300000
SMD[1,9]	0.3158	0.1016	0.001373	0.1185	0.3146	0.5161	50001	300000
SMD[2,3]	0.06206	0.08823	9.08E-04	-0.1136	0.06264	0.2329	50001	300000
SMD[2,4]	0.06366	0.07201	7.12E-04	-0.08651	0.0662	0.1979	50001	300000
SMD[2,5]	0.09033	0.09222	9.40E-04	-0.09567	0.09159	0.2702	50001	300000
SMD[2,6]	0.2749	0.1419	0.00171	-0.01044	0.277	0.5468	50001	300000
SMD[2,7]	0.1352	0.1729	0.001971	-0.2112	0.1373	0.4687	50001	300000
SMD[2,8]	-0.03341	0.07277	8.78E-04	-0.1758	-0.03375	0.1096	50001	300000
SMD[2,9]	0.1673	0.09305	0.001216	-0.01567	0.1668	0.3507	50001	300000
SMD[3,4]	0.001597	0.09706	9.48E-04	-0.1925	0.002986	0.1889	50001	300000
SMD[3,5]	0.02827	0.1072	0.001072	-0.184	0.02916	0.2399	50001	300000
SMD[3,6]	0.2129	0.1516	0.001809	-0.08953	0.2151	0.5086	50001	300000
SMD[3,7]	0.07312	0.1809	0.002089	-0.286	0.075	0.4288	50001	300000
SMD[3,8]	-0.09547	0.08589	8.28E-04	-0.2606	-0.09699	0.07861	50001	300000
SMD[3,9]	0.1053	0.1205	0.001472	-0.1286	0.1052	0.3456	50001	300000
SMD[4,5]	0.02667	0.07351	7.00E-04	-0.1157	0.02564	0.1734	50001	300000
SMD[4,6]	0.2113	0.1296	0.001471	-0.04172	0.2105	0.4689	50001	300000
SMD[4,7]	0.07152	0.1629	0.001772	-0.2497	0.07164	0.3947	50001	300000
SMD[4,8]	-0.09707	0.08821	9.63E-04	-0.2632	-0.09863	0.0838	50001	300000
SMD[4,9]	0.1037	0.1124	0.001353	-0.1139	0.1033	0.3276	50001	300000
SMD[5,6]	0.1846	0.1068	0.001154	-0.02657	0.1857	0.3941	50001	300000
SMD[5,7]	0.04485	0.1459	0.001525	-0.2447	0.04581	0.3336	50001	300000
SMD[5,8]	-0.1237	0.1006	0.001083	-0.3168	-0.1262	0.08063	50001	300000
SMD[5,9]	0.077	0.1254	0.001505	-0.1633	0.07616	0.3231	50001	300000

SMD[6,7]	-0.1397	0.09953	8.16E-04	-0.3344	-0.1402	0.05604	50001	300000
SMD[6,8]	-0.3083	0.1471	0.001815	-0.5896	-0.3105	-0.01227	50001	300000
SMD[6,9]	-0.1076	0.1657	0.002076	-0.4322	-0.1108	0.2208	50001	300000
SMD[7,8]	-0.1686	0.177	0.002065	-0.5121	-0.1713	0.1862	50001	300000
SMD[7,9]	0.03215	0.192	0.002265	-0.3415	0.0301	0.4168	50001	300000
SMD[8,9]	0.2007	0.1059	0.00146	-0.00772	0.2022	0.4067	50001	300000

Efficacy on behavior

NO.	Interventions
1	Placebo
2	Donepezil 10 mg
3	Galantamine 24 mg
4	Rivastigmine 12 mg
5	Rivastigmine patch 10cm ²
6	Memantine 20 mg

node	mean	sd	MC error	2.50%	median	97.50%	start	sample
SMD[1,2]	-0.09703	0.07445	3.12E-04	-0.2462	-0.09676	0.05082	50001	300000
SMD[1,3]	-0.1536	0.117	4.01E-04	-0.3884	-0.1534	0.08201	50001	300000
SMD[1,4]	-0.08403	0.1156	3.68E-04	-0.3158	-0.08455	0.1516	50001	300000
SMD[1,5]	-0.03919	0.1363	4.76E-04	-0.3122	-0.03988	0.2374	50001	300000
SMD[1,6]	-0.07981	0.07558	3.55E-04	-0.2354	-0.07817	0.06634	50001	300000
SMD[2,3]	-0.05653	0.1385	5.15E-04	-0.3337	-0.05722	0.2228	50001	300000
SMD[2,4]	0.013	0.1155	3.51E-04	-0.2187	0.01168	0.25	50001	300000
SMD[2,5]	0.05784	0.1419	4.99E-04	-0.2263	0.05733	0.3472	50001	300000
SMD[2,6]	0.01723	0.09894	4.55E-04	-0.1842	0.01891	0.21	50001	300000
SMD[3,4]	0.06953	0.164	5.48E-04	-0.2609	0.06945	0.4013	50001	300000
SMD[3,5]	0.1144	0.1793	6.30E-04	-0.2463	0.1142	0.4766	50001	300000
SMD[3,6]	0.07376	0.1392	5.30E-04	-0.2108	0.07559	0.347	50001	300000
SMD[4,5]	0.04484	0.1131	3.88E-04	-0.1836	0.04513	0.2726	50001	300000
SMD[4,6]	0.004227	0.136	5.20E-04	-0.2767	0.006938	0.2703	50001	300000
SMD[5,6]	-0.04062	0.1545	5.87E-04	-0.358	-0.03745	0.2632	50001	300000

Efficacy on global changes

NO.	Interventions
1	Placebo
2	Donepezil 5 mg
3	Donepezil 10 mg
4	Galantamine 24 mg
5	Galantamine 32 mg
6	Rivastigmine 12 mg
7	Rivastigmine patch 10cm ^{2}
8	Rivastigmine patch 5cm ^{2}
9	Rivastigmine patch 15cm ^{2}
10	Memantine 20 mg
11	Donepezil 23 mg

node	mean	sd	MC error	2.50%	median	97.50%	start	sample
or[1,2]	1.975	0.3152	0.001571	1.428	1.95	2.671	50001	300000
or[1,3]	2.146	0.3052	0.001577	1.608	2.124	2.806	50001	300000
or[1,4]	1.34	0.2142	0.001047	0.9606	1.325	1.807	50001	300000
or[1,5]	1.477	0.3569	0.001787	0.8897	1.44	2.285	50001	300000
or[1,6]	1.803	0.319	0.001142	1.258	1.774	2.517	50001	300000
or[1,7]	1.568	0.3145	0.001232	1.041	1.537	2.279	50001	300000
or[1,8]	1.551	0.4901	0.002567	0.8098	1.481	2.712	50001	300000
or[1,9]	2.765	1.344	0.006555	1.04	2.503	6.061	50001	300000
or[1,10]	1.24	0.4574	0.002016	0.586	1.164	2.337	50001	300000
or[1,11]	1.991	0.545	0.002799	1.097	1.935	3.22	50001	300000
or[2,3]	1.103	0.1805	8.69E-04	0.7874	1.09	1.498	50001	300000
or[2,4]	0.6956	0.1579	7.66E-04	0.4321	0.6798	1.052	50001	300000
or[2,5]	0.7667	0.2236	0.00108	0.4141	0.7393	1.282	50001	300000
or[2,6]	0.9355	0.2245	9.54E-04	0.571	0.9099	1.451	50001	300000
or[2,7]	0.8137	0.2101	8.98E-04	0.4768	0.7883	1.3	50001	300000
or[2,8]	0.8051	0.2888	0.001442	0.3831	0.7599	1.497	50001	300000
or[2,9]	1.435	0.746	0.003515	0.5036	1.283	3.264	50001	300000
or[2,10]	0.6431	0.2614	0.001177	0.2819	0.5968	1.277	50001	300000
or[2,11]	1.024	0.2931	0.001407	0.5486	0.9931	1.69	50001	300000
or[3,4]	0.6371	0.1373	6.72E-04	0.4063	0.6236	0.9454	50001	300000
or[3,5]	0.7022	0.1984	9.10E-04	0.3877	0.6777	1.16	50001	300000
or[3,6]	0.8569	0.1965	8.30E-04	0.537	0.8349	1.305	50001	300000
or[3,7]	0.7454	0.1849	7.93E-04	0.4483	0.7235	1.172	50001	300000
or[3,8]	0.7375	0.2584	0.001341	0.3583	0.6972	1.355	50001	300000
or[3,9]	1.314	0.6752	0.003253	0.4679	1.179	2.977	50001	300000
or[3,10]	0.5894	0.2366	0.001101	0.262	0.5483	1.16	50001	300000
or[3,11]	0.9277	0.2147	8.97E-04	0.557	0.9122	1.399	50001	300000
or[4,5]	1.116	0.2678	0.001271	0.6793	1.086	1.725	50001	300000

or[4,6]	1.38	0.336	0.001429	0.845	1.338	2.158	50001	300000
or[4,7]	1.2	0.3148	0.001353	0.708	1.16	1.932	50001	300000
or[4,8]	1.188	0.4308	0.002174	0.5691	1.116	2.231	50001	300000
or[4,9]	2.117	1.112	0.005399	0.7484	1.885	4.862	50001	300000
or[4,10]	0.9493	0.3916	0.001779	0.4145	0.8785	1.9	50001	300000
or[4,11]	1.523	0.4893	0.002394	0.7658	1.458	2.663	50001	300000
or[5,6]	1.292	0.3991	0.001824	0.6935	1.231	2.241	50001	300000
or[5,7]	1.124	0.3645	0.001679	0.5851	1.068	1.991	50001	300000
or[5,8]	1.112	0.4604	0.002269	0.4841	1.027	2.235	50001	300000
or[5,9]	1.982	1.129	0.005265	0.6474	1.737	4.777	50001	300000
or[5,10]	0.8888	0.4072	0.00189	0.355	0.8106	1.89	50001	300000
or[5,11]	1.425	0.531	0.002394	0.6475	1.344	2.697	50001	300000
or[6,7]	0.8826	0.1736	6.58E-04	0.5878	0.8665	1.271	50001	300000
or[6,8]	0.8796	0.2987	0.001455	0.4364	0.8343	1.593	50001	300000
or[6,9]	1.568	0.7929	0.0037	0.5668	1.408	3.518	50001	300000
or[6,10]	0.7088	0.2953	0.001258	0.3043	0.6564	1.423	50001	300000
or[6,11]	1.138	0.3751	0.001724	0.5565	1.09	2.012	50001	300000
or[7,8]	1.007	0.3134	0.001493	0.5301	0.9634	1.748	50001	300000
or[7,9]	1.795	0.8664	0.003924	0.6747	1.627	3.928	50001	300000
or[7,10]	0.8218	0.353	0.001509	0.3439	0.7579	1.679	50001	300000
or[7,11]	1.32	0.4532	0.002086	0.6261	1.258	2.38	50001	300000
or[8,9]	1.781	0.6019	0.002152	0.893	1.69	3.22	50001	300000
or[8,10]	0.877	0.4451	0.002068	0.3163	0.7847	1.98	50001	300000
or[8,11]	1.408	0.6075	0.003089	0.5634	1.307	2.852	50001	300000
or[9,10]	0.5469	0.3583	0.001632	0.1519	0.4639	1.423	50001	300000
or[9,11]	0.8774	0.5066	0.002381	0.2675	0.772	2.108	50001	300000
or[10,11]	1.816	0.853	0.004579	0.6688	1.659	3.876	50001	300000

Supplementary 7:
Network plot for each outcome

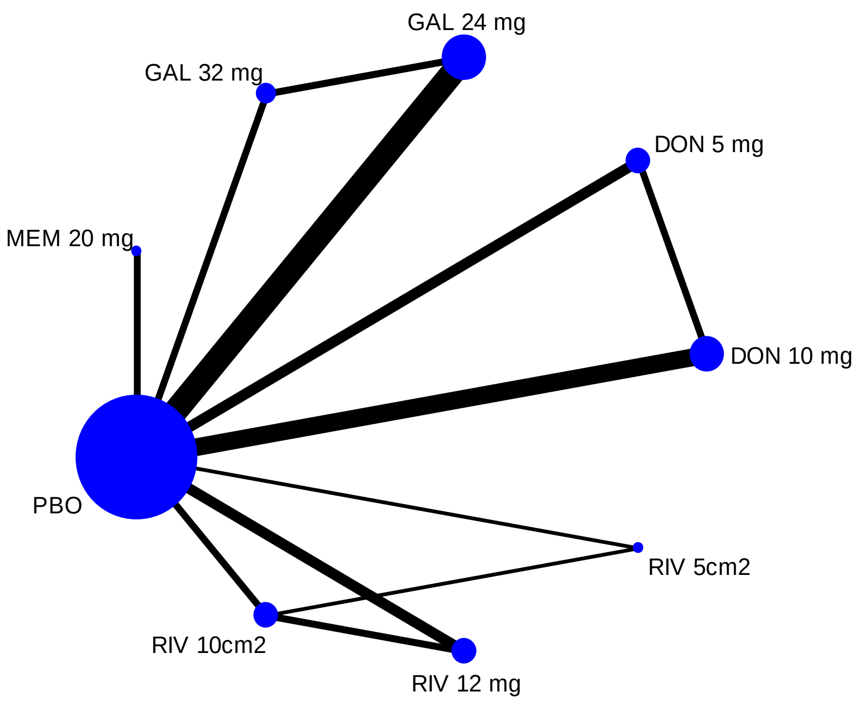

Network plot of tolerability for mild to moderate AD

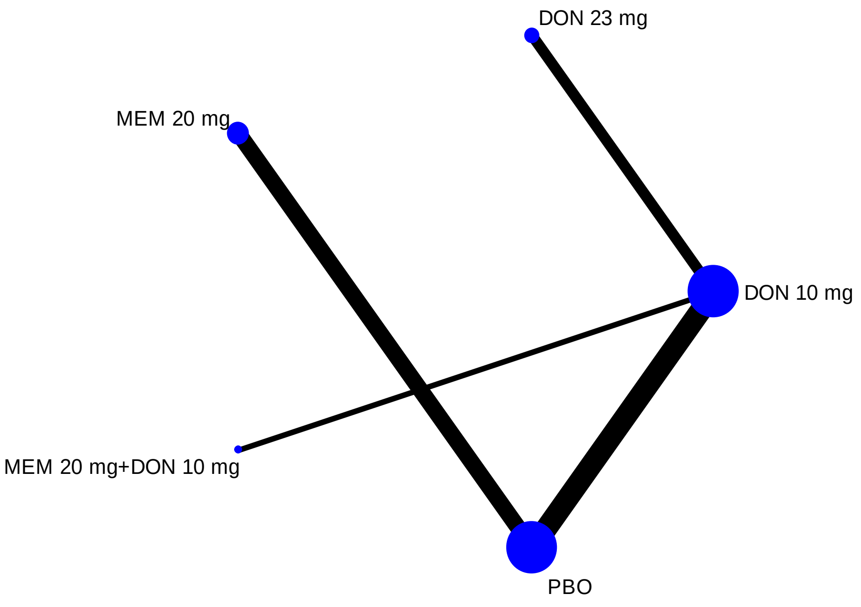

Network plot of tolerability for moderate to severe AD

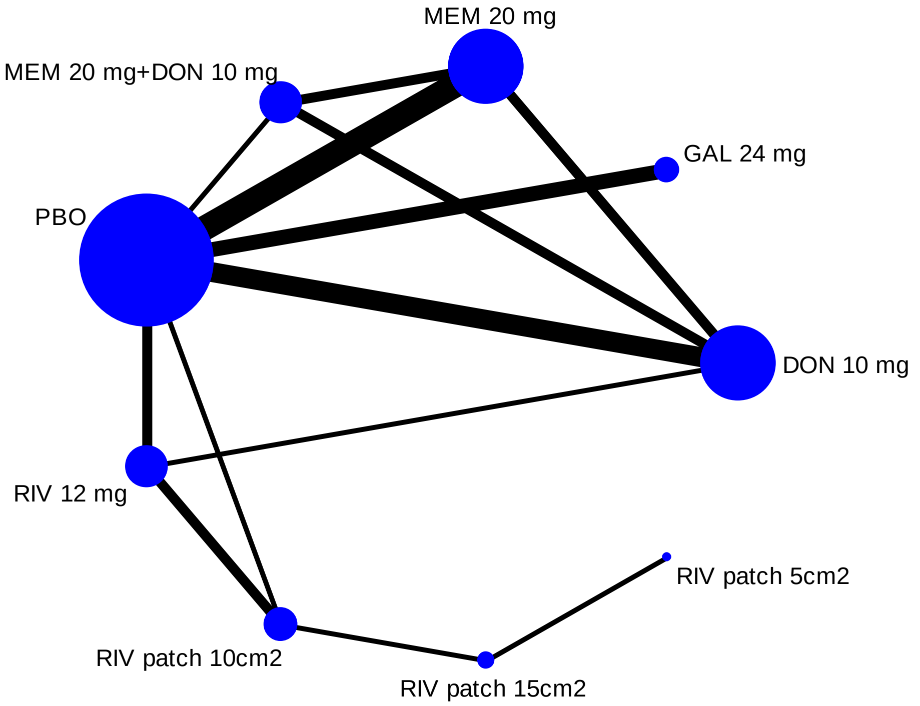

Network plot of efficacy on function

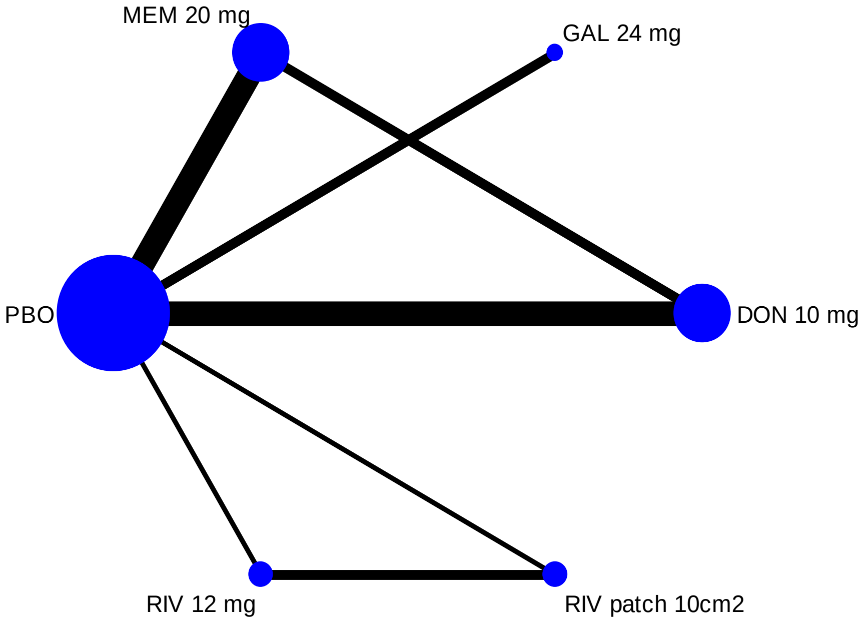

Network plot of efficacy on behavior

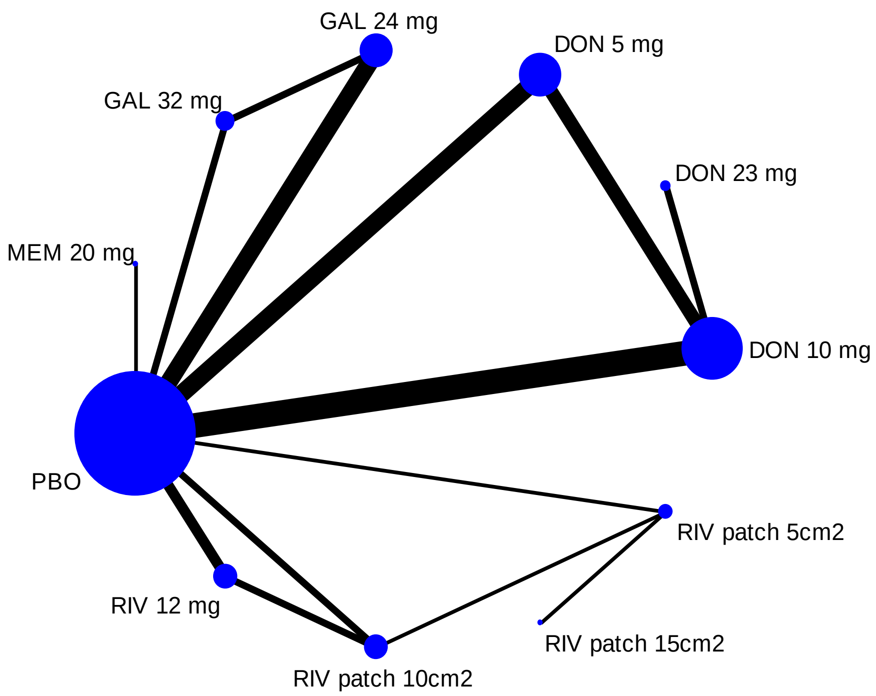

Network plot of efficacy on global changes

Supplementary 8:
Treatment ranking and rankograms for each outcome

Efficacy on cognition for mild to moderate AD

Treatment ranking

Treatment	Abbreviation	SUCRA(%)	PrBest	MeanRank
Placebo	PBO	0.6	0.0	9.9
Donepezil 5 mg	DON 5 mg	53.2	0.4	5.2
Donepezil 10 mg	DON 10 mg	72.8	3.5	3.5
Galantamine 24 mg	GAL 24 mg	90.9	37.3	1.8
Galantamine 32 mg	GAL 32 mg	91.1	49.5	1.8
Rivastigmine 12 mg	RIV 12 mg	44.9	0.1	6.0
Rivastigmine patch 10 cm^2	RIV 10 cm^2	35.0	0.0	6.9
Rivastigmine patch 15 cm^2	RIV 15 cm^2	59.8	9.0	4.6
Rivastigmine patch 5 cm^2	RIV 5 cm^2	20.0	0.1	8.2
Memantine 20 mg	MEM 20 mg	31.7	0.1	7.1

Adverse events for mild to moderate AD

Treatment ranking

Treatment	Abbreviation	SUCRA(%)	PrBest	MeanRank
Placebo	PBO	91.3	37.9	1.8
Donepezil 5 mg	DON 5 mg	78.1	14.0	3.0
Donepezil 10 mg	DON 10 mg	53.7	0.4	5.2
Galantamine 24 mg	GAL 24 mg	50.4	0.0	5.5
Galantamine 32 mg	GAL 32 mg	15.2	0.0	8.6
Rivastigmine 12 mg	RIV 12 mg	8.4	0.0	9.2
Rivastigmine patch 10 cm^2	RIV 10 cm^2	47.8	0.3	5.7
Rivastigmine patch 15 cm^2	RIV 15 cm^2	23.5	0.9	7.9
Rivastigmine patch 5 cm^2	RIV 5cm^2	44.3	2.8	6.0
Memantine 20 mg	MEM 20 mg	87.3	43.6	2.1

Efficacy on cognition for moderate to severe AD

Treatment ranking

Treatment	Abbreviation	SUCRA (%)	PrBest	MeanRank
Placebo	PBO	0.3	0.0	5.0
Donepezil 10 mg	DON 10 mg	57.3	2.9	2.7
Donepezil 23 mg	DON 23 mg	69.6	20.7	2.2
Memantine 20 mg	MEM 20 mg	31.3	1.2	3.7
Memantine 20 mg + Donepezil 10 mg	MEM 20 mg + DON10 mg	91.5	75.2	1.3

Rankograms

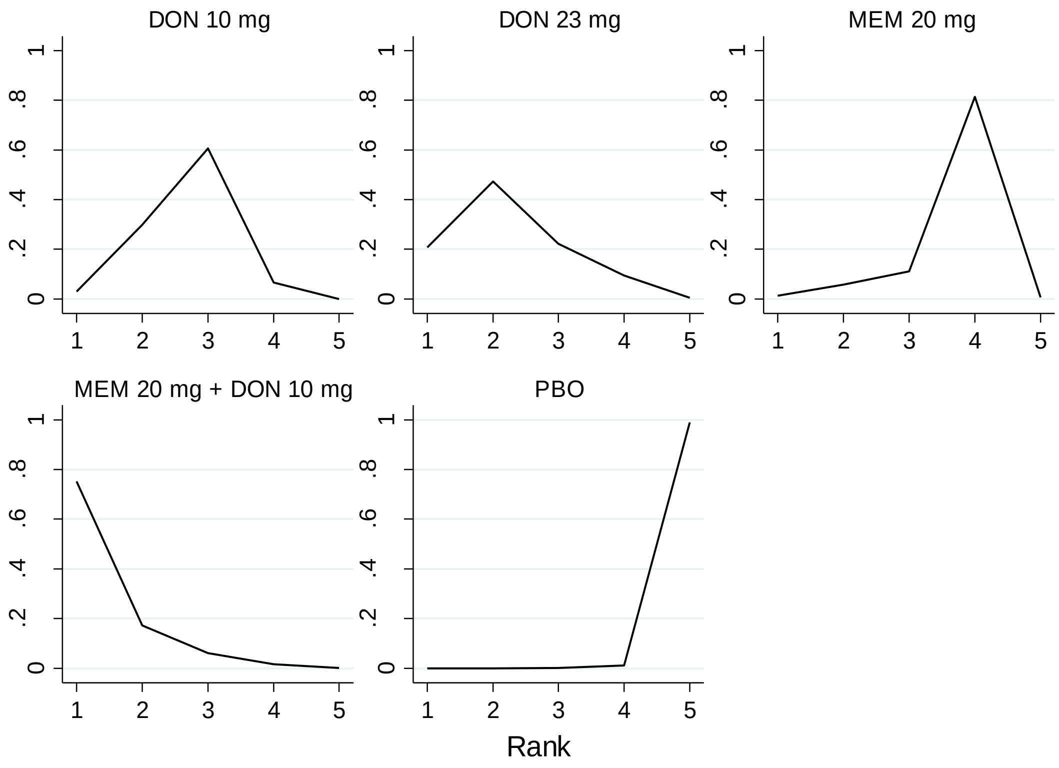

Adverse events for moderate to severe AD

Treatment ranking

Treatment	Abbreviation	SUCRA (%)	PrBest	MeanRank
Placebo	PBO	89.8	59.5	1.4
Donepezil 10 mg	DON 10 mg	50.3	0.3	3.0
Donepezil 23 mg	DON 23 mg	4.5	0.0	4.8
Memantine 20 mg	MEM 20 mg	83.5	40.0	1.7
Memantine 20 mg + Donepezil 10 mg	MEM 20 mg + DON 10 mg	21.9	0.1	4.1

Rankograms

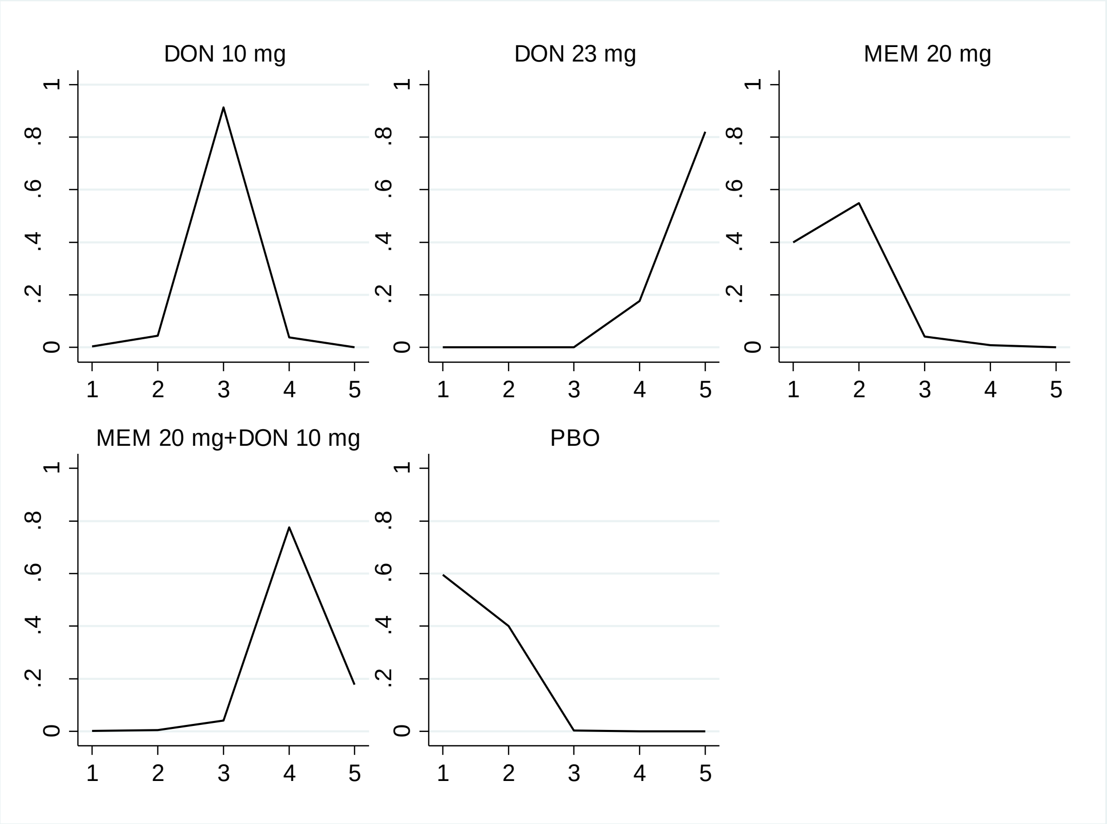

Efficacy on function

Treatment ranking

Treatment	Abbreviation	SUCRA (%)	PrBest	MeanRank
Placebo	PBO	1.1	0.0	8.9
Donepezil 10 mg	DON 10 mg	31.2	0.1	6.5
Galantamine 24 mg	GAL 24 mg	51.3	2.3	4.9
Rivastigmine 12 mg	RIV 12 mg	51.4	0.7	4.9
Rivastigmine patch 10 cm ²	RIV 10 cm ²	58.4	0.7	4.3
Rivastigmine patch 15 cm ²	RIV 15 cm ²	93.2	67.9	1.5
Rivastigmine patch 5 cm ²	RIV 5 cm ²	63.5	5.8	3.9
Memantine 20 mg	MEM 20 mg	23.3	0.1	7.1
Memantine 2 0 mg + Donepezil 10 mg	MEM 20 mg + DON 10 mg	76.4	22.3	2.9

Rankograms

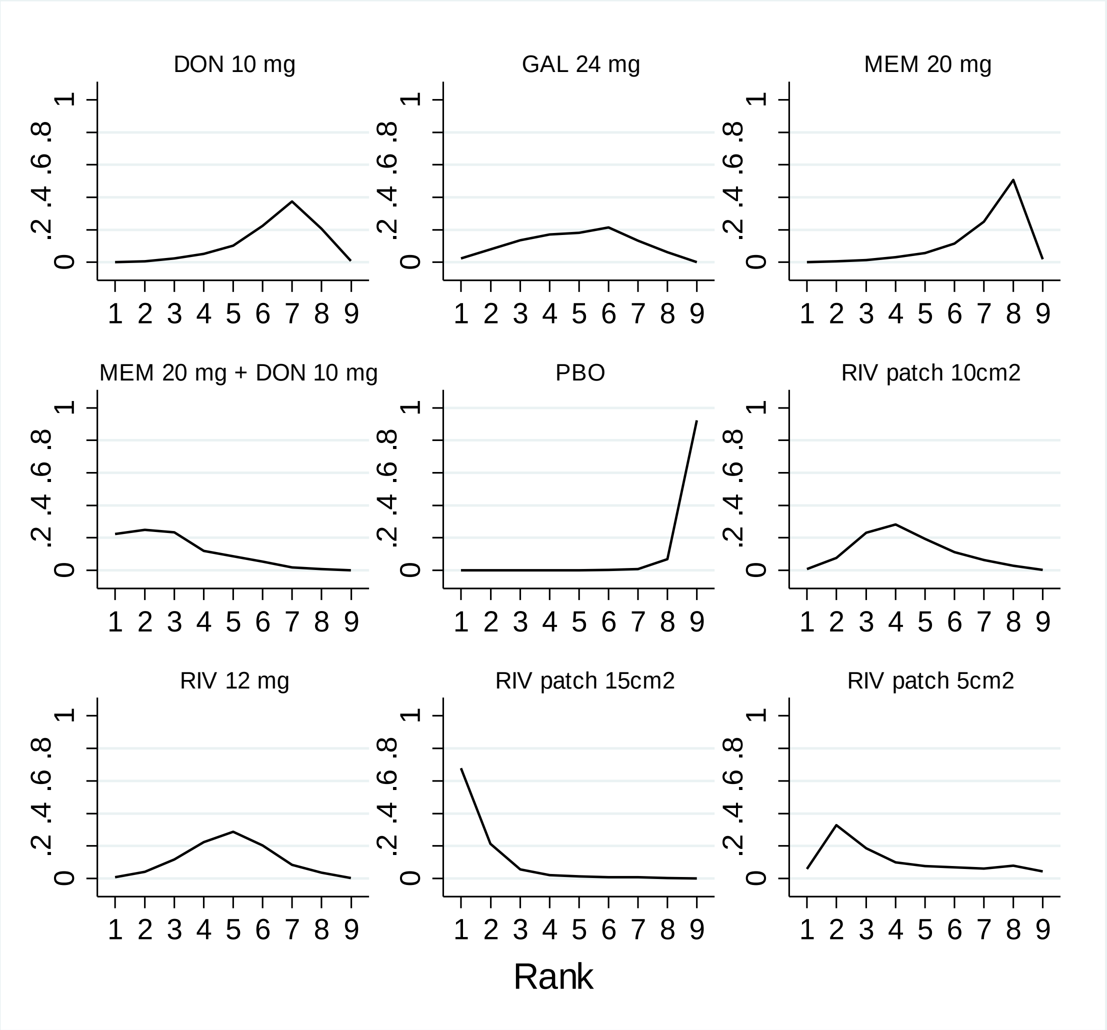

Efficacy on behavior

Treatment ranking

Treatment	Abbreviation	SUCRA (%)	PrBest	MeanRank
Placebo	PBO	22.8	0.1	4.9
Donepezil 10 mg	DON 10 mg	65.6	19.6	2.7
Galantamine 24 mg	GAL 24 mg	72.1	41.9	2.4
Rivastigmine 12 mg	RIV 12 mg	42.1	13	3.9
Rivastigmine patch 10 cm ²	RIV 10 cm ²	33.1	7.7	4.3
Memantine 20 mg	MEM 20 mg	64.3	17.8	2.8

Rankograms

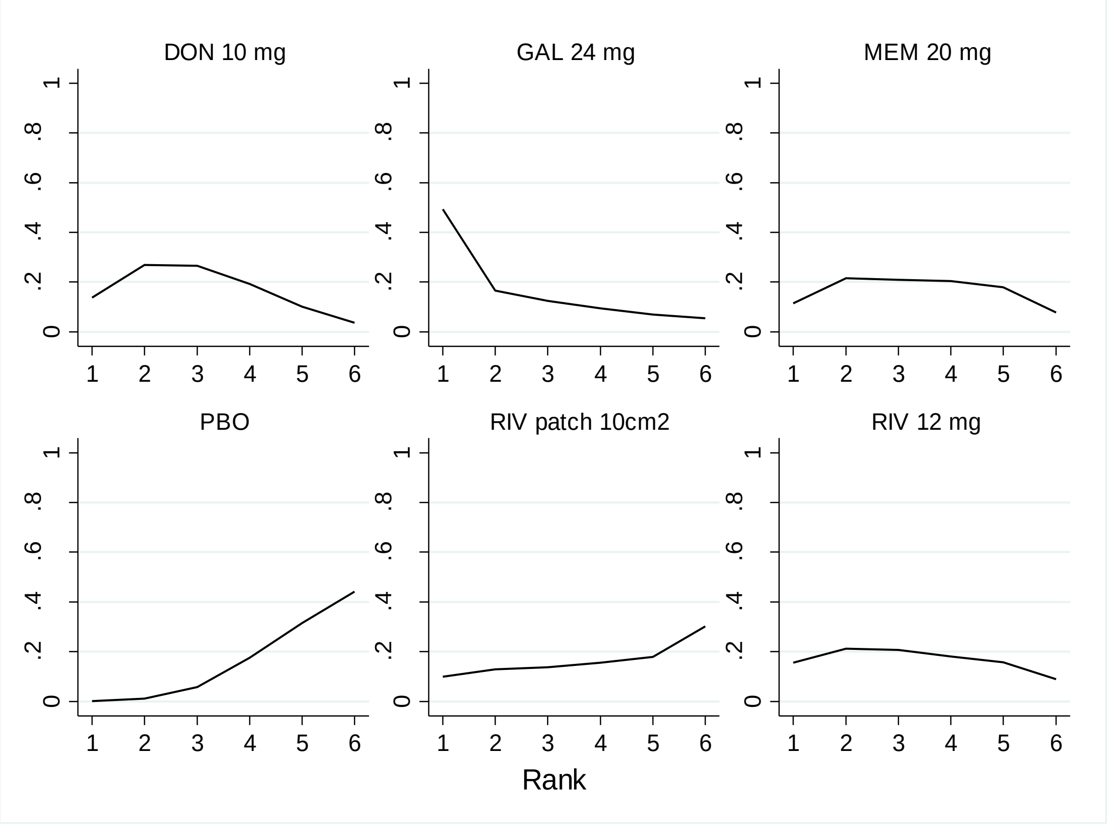

Efficacy on global changes

Treatment ranking

Treatment	Abbreviation	SUCRA (%)	PrBest	MeanRank
Placebo	PBO	5.7	0.0	10.4
Donepezil 5 mg	DON 5 mg	71.3	7.2	3.9
Donepezil 10 mg	DON 10 mg	81.7	14.8	2.8
Galantamine 24 mg	GAL 24 mg	29.4	0.1	8.1
Galantamine 32 mg	GAL 32 mg	39.2	1.5	7.1
Rivastigmine 12 mg	RIV 12 mg	61.8	3.8	4.8
Rivastigmine patch 10 cm ²	RIV 10 cm ²	44.5	0.7	6.6
Rivastigmine patch 15 cm ²	RIV 15 cm ²	83.6	56.3	2.6
Rivastigmine patch 5 cm ²	RIV 5 cm ²	41.4	0.4	6.9
Memantine 20 mg	MEM 20 mg	23.5	1.6	8.6
Donepezil 23 mg	DON 23 mg	67.9	13.6	4.2

Rankograms

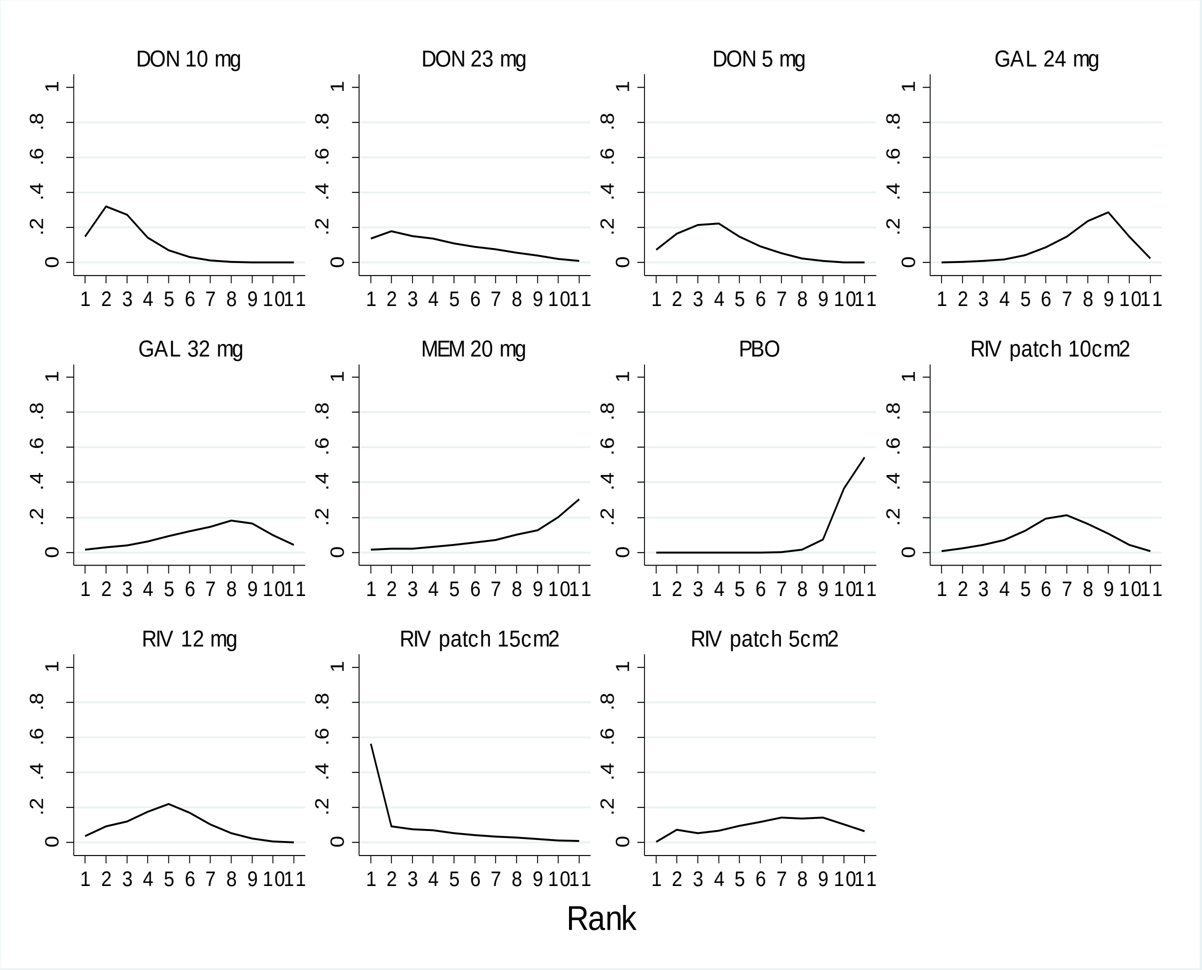

Supplementary 9:
Assessment of the inconsistency results for each outcome

Efficacy on cognition for mild to moderate AD

Loop	IF	Z_value	P_value	95% CI	τ^2
DON 10 mg- DON 5 mg- PBO	0.338	2.300	0.021	(0.05, 0.63)	0.006
DON 10 mg- MEM 20 mg- PBO	0.248	0.939	0.348	(0.00, 0.77)	0.018
GAL 24 mg- GAL 32 mg- PBO	0.239	1.143	0.253	(0.00, 0.65)	0.024
PBO- RIV 10cm ^{2}- RIV 12 mg	0.067	0.612	0.541	(0.00, 0.28)	0.000
PBO- RIV 10cm ^{2}- RIV 5cm ^{2}	0.002	0.016	0.988	(0.00, 0.30)	0.000

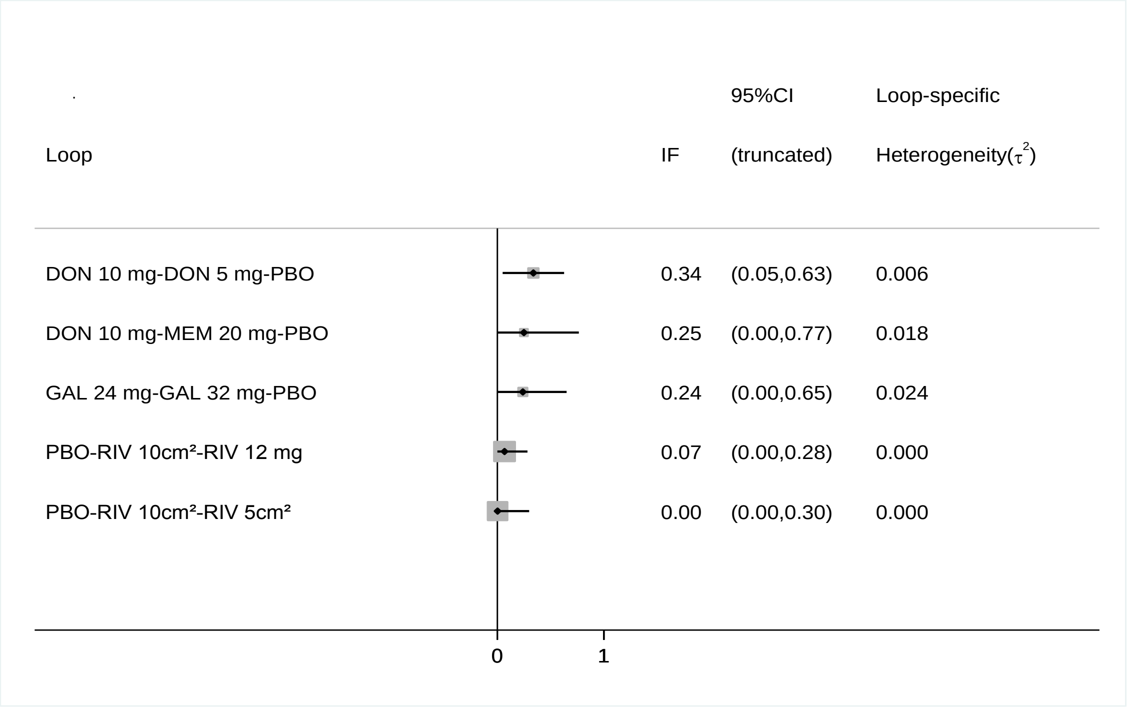

Efficacy on cognition for moderate to severe AD

Loop	IF	Z_value	P_value	95% CI	τ^2
MEM20mg - MEM20mg+DON10mg-PBO	0.209	0.732	0.464	(0.00, 0.77)	0.000
DON10mg - MEM20mg+DON10mg-PBO	0.157	0.406	0.685	(0.00, 0.91)	0.030
DON10mg - MEM20mg-PBO	0.021	0.065	0.949	(0.00, 0.67)	0.041
DON10mg - MEM20mg- MEM20mg+DON10mg	…	…	…	…	0.000

Note: Loop DON 10 mg-MEM 20 mg-MEM 20 mg+DON 10 mg is formed only by multi-arm trial(s) - Consistent by definition

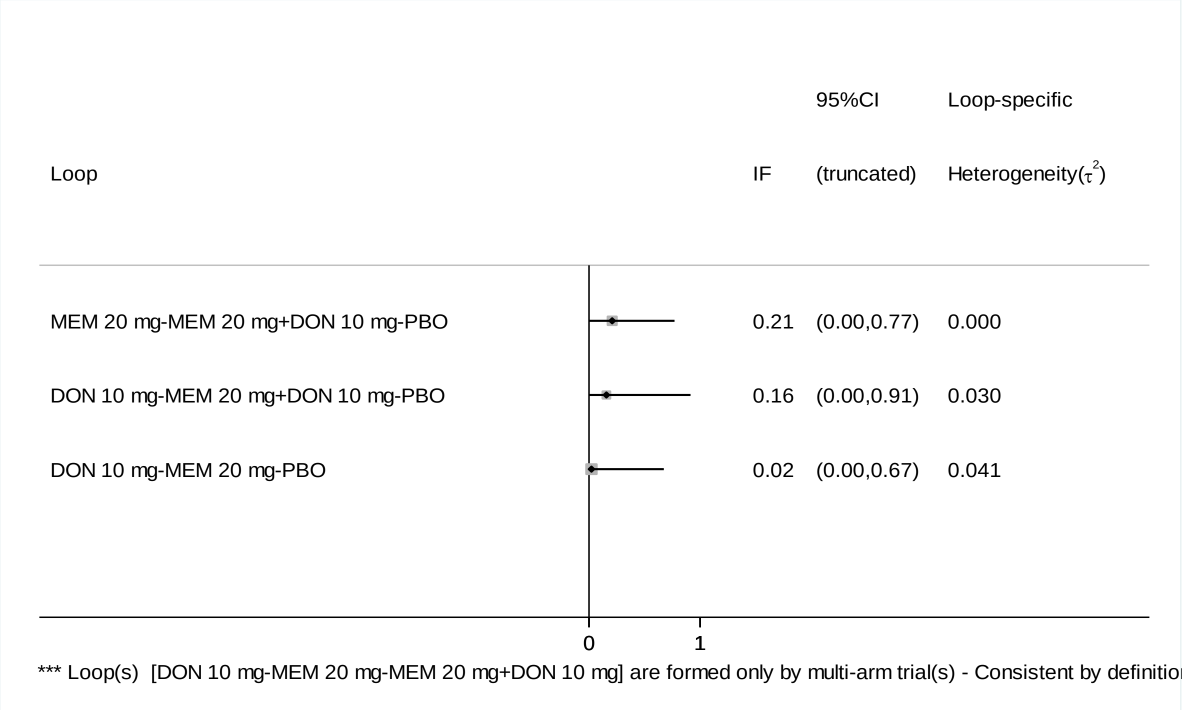

All-cause adverse events for mild to moderate AD

Loop	IF	Z_value	P_value	95% CI	τ^2
DON10mg- DON5mg- PBO	0.643	1.914	0.056	(0.00,1.30)	0.021
PBO- RIV10cm^2 RIV12mg	0.500	1.880	0.060	(0.00,1.02)	0.000
PBO- RIV10cm^2 RIV5cm^2	0.426	1.154	0.249	(0.00,1.15)	0.000
GAL24mg- GAL32mg- PBO	0.349	0.905	0.366	(0.00,1.11)	0.025

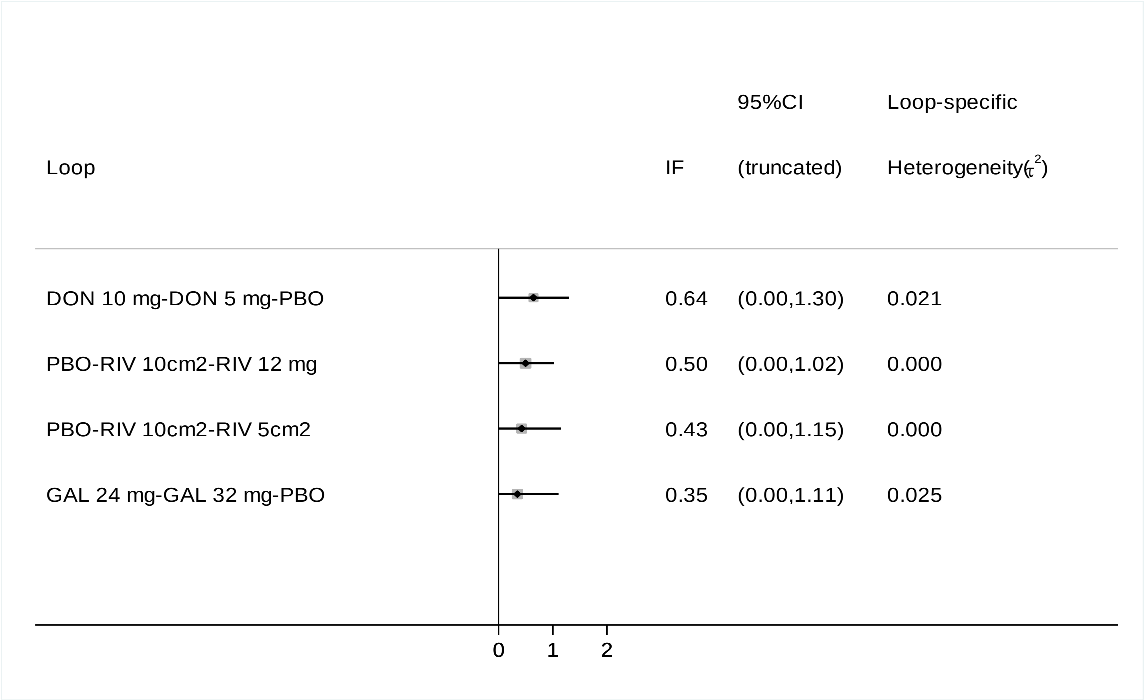

Efficacy on function

Loop	IF	Z_value	P_value	95% CI	τ^2
MEM20mg-MEM20mg+DON10mg-PBO	0.304	1.072	0.284	(0.00, 0.86)	0.006
DON10mg-PBO-RIV12mg	0.170	1.134	0.257	(0.00, 0.46)	0.003
DON10mg-MEM20mg+DON10mg-PBO	0.166	0.740	0.459	(0.00, 0.60)	0.000
DON10mg-MEM20mg-MEM20mg+DON10mg	0.124	0.548	0.584	(0.00, 0.57)	0.000
DON10mg-MEM20mg-PBO	0.075	0.479	0.632	(0.00, 0.38)	0.001
PBO-RIV10cm ^{2}RIV12mg	0.060	0.392	0.695	(0.00, 0.36)	0.000

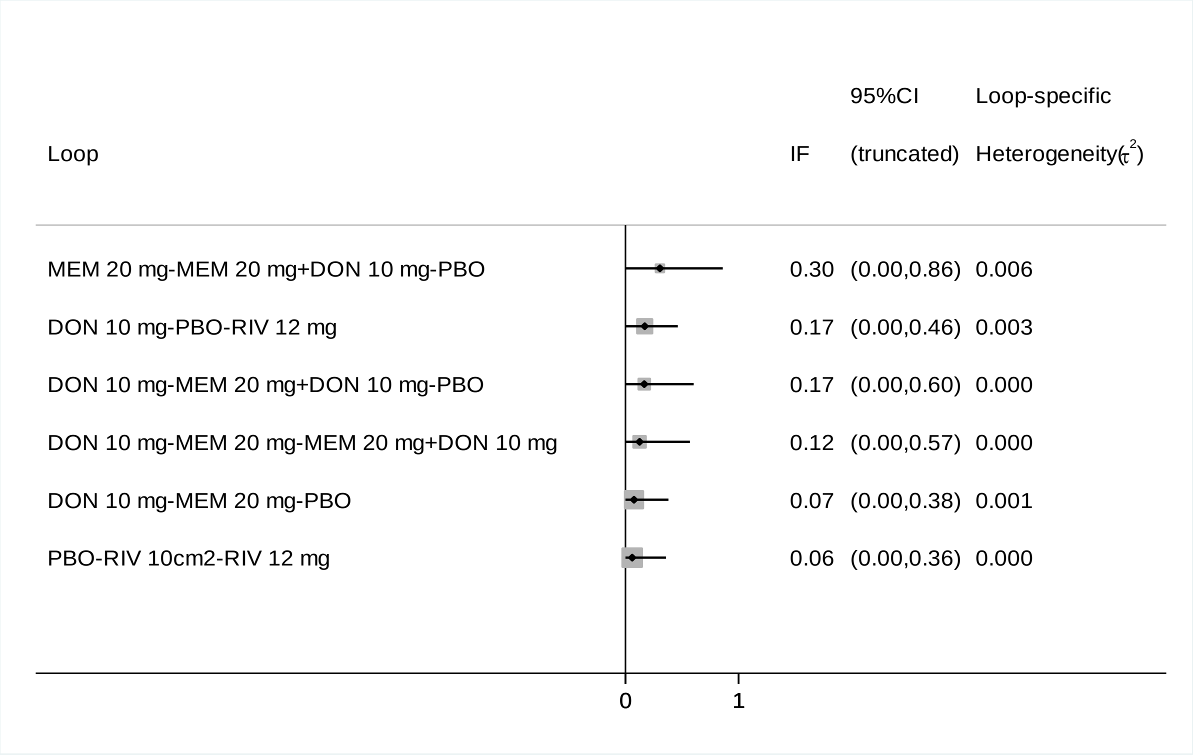

Efficacy on behavior

Loop	IF	Z_value	P_value	95% CI	τ^2
DON10mg-MEM20mg-PBO	0.385	1.597	0.110	(0.00, 0.86)	0.020
DON10mg-PBO- RIV12mg	0.151	0.527	0.598	(0.00, 0.71)	0.030
PBO-RIV12mg- RIV10cm ²	0.017	0.111	0.912	(0.00,0.32)	0.000

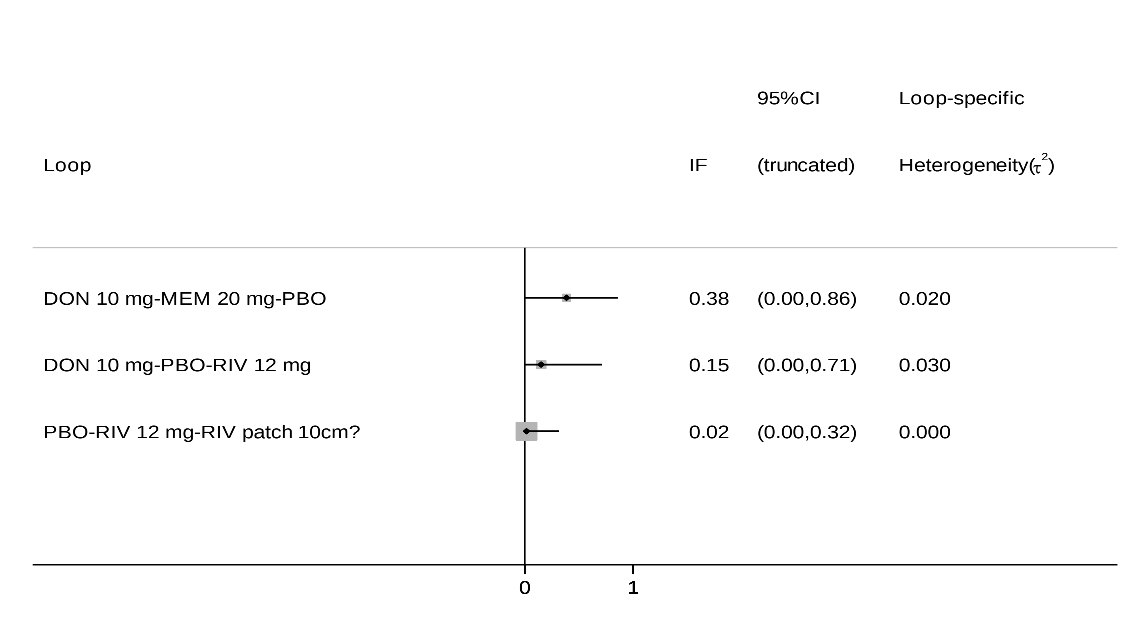

Efficacy on global changes

Loop	IF	Z_value	P_value	95% CI	τ^2
DON 10 mg-DON 5 mg-PBO	0.557	1.766	0.077	(0.00,1.18)	0.045
PBO-RIV 10cm ^{2}RIV 12 mg	0.419	1.656	0.098	(0.00,0.91)	0.000
PBO-RIV 10cm ^{2}RIV 5cm ^{2}	0.289	0.796	0.426	(0.00,1.00)	0.000
GAL 24 mg-GAL 32 mg-PBO	0.017	0.038	0.970	(0.00,0.91)	0.088

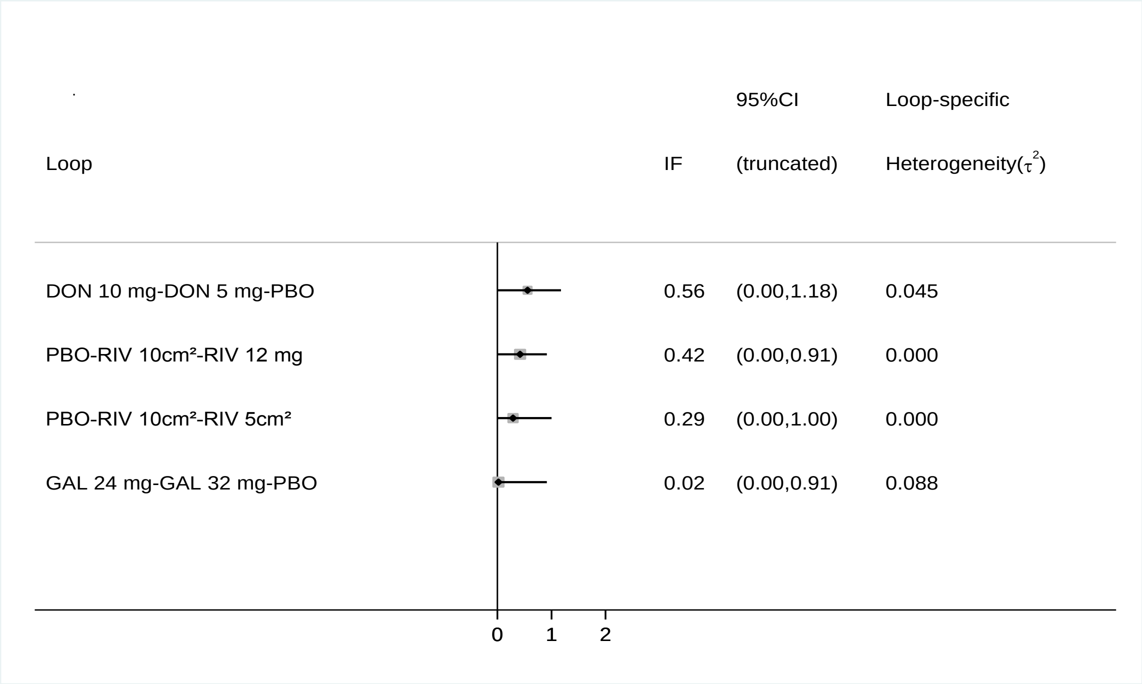

Evaluation the inconsistency by node-splitting model

Efficacy on cognition for mild to moderate AD

Comparisons	Direct		Indirect		Differences			τ^2
	SMD	SE	SMD	SE	SMD	SE	P-value	
PBO vs DON 5 mg	-0.371	0.054	0.308	0.195	-0.679	0.203	0.001	5.00e-09
PBO vs DON 10 mg	-0.417	0.057	-0.322	0.158	-0.095	0.167	0.571	0.053
PBO vs GAL 24 mg	…	…	…	…	…	…	…	…
PBO vs GAL 32 mg	-0.551	0.079	-0.350	0.185	0.201	0.202	0.318	0.052
PBO vs RIV 12 mg	-0.296	0.062	-0.271	0.153	-0.025	0.165	0.879	0.060
PBO vs RIV 10 cm^2	-0.242	0.074	-0.302	0.131	0.060	0.150	0.689	0.058
PBO vs RIV 5 cm^2	-0.159	0.106	-0.217	0.246	0.058	0.399	0.882	0.132
PBO vs MEM 20 mg	-0.188	0.077	-0.470	0.168	0.282	0.185	0.127	0.035
DON 5 mg vs DON 10 mg	-0.168	0.059	0.360	0.145	-0.528	0.157	0.001	1.98e-09
DON 10 mg vs MEM 20 mg	-0.036	0.159	0.246	0.094	-0.282	0.185	0.127	0.035
GAL24 mg vs GAL 32 mg	0.014	0.078	-0.187	0.186	0.201	0.202	0.318	0.052
RIV 12 mg vs RIV 10 cm^2	0.016	0.076	0.090	0.127	-0.075	0.148	0.614	0.057
RIV 10cm^2vs RIV 5 cm^2	0.080	0.105	0..138	0.247	-0.058	0.268	0.828	0.060
RIV 10cm^2vs RIV 15 cm^2	-0.098	0.099	0.512	73.781	-0.610	73.781	0.993	0.049

Efficacy on cognition for moderate to severe AD

Comparisons	Direct		Indirect		Differences			τ^2
	SMD	SE	SMD	SE	SMD	SE	P-value	
PBO vs DON 10 mg	0.548	0.093	0.223	0.346	0.325	0.357	0.363	0.164
PBO vs MEM 20 mg	0.327	0.129	0.402	0.458	-0.075	0.475	0.874	0.179
PBO vs MEM 20 mg +DON 10 mg	0.666	0.260	0.804	0.208	-0.137	0.335	0.681	0.178
DON 10mg vs DON 23 mg	0.064	0.132	-1.074	44.686	1.138	44.686	0.980	0.166
DON 10mg vs MEM 20 mg	-0.235	0.265	-0.180	0.172	-0.055	0.316	0.862	0.179
DON 10mg vs MEM 20 mg +DON 10 mg	0.230	0.162	0.155	0.445	0.07	0.475	0.874	0.178
MEM 20 mg vs MEM 20 mg +DON 10 mg	0.259	0.253	0.584	0.252	-0.325	0.357	0.363	0.164

All-cause adverse events for mild to moderate AD

Comparisons	Direct		Indirect		Differences			τ^2
	Log OR	SE	Log OR	SE	Log OR	SE	P-value	
PBO vs DON 5 mg	-2.185	0.447	1.498	1.472	-3.684	1.538	0.017	0.650
PBO vs DON 10 mg	-2.262	0.446	-1.922	1.252	-0.340	1.3326	0.797	0.789
PBO vs GAL 24 mg	…	…	…	…	…	…	…	…
PBO vs GAL 32 mg	-3.312	0.664	-5.839	1.508	2.526	1.646	0.125	0.713
PBO vs RIV 12 mg	-2.044	0.581	-1.383	1.331	-0.661	1.452	0.649	0.795
PBO vs RIV 10cm^2	-1.384	0.662	-2.179	1.190	0.794	1.362	0.560	0.788

PBO vs RIV 5 cm ^{2}	-0.800	0.905	-2.065	2.179	1.265	2.361	0.592	0.793
PBO vs MEM 20 mg	-1.336	0.749	-2.566	1.340	1.230	1.535	0.423	0.765
DON 5 mg vs DON 10 mg	-0.893	0.519	1.704	1.048	-2.597	1.169	0.026	0.666
DON 10 mg vs MEM 20 mg	-0.240	1.268	0.990	0.865	-1.230	1.535	0.423	0.765
GAL24 mg vs GAL 32 mg	-1.812	0.646	0.715	1.531	-2.526	1.646	0.125	0.713
RIV 12 mg vs RIV 10 cm ^{2}	0.099	0.681	1.110	1.137	1.011	1.325	0.445	0.781
RIV 10 cm ^{2}vs RIV 5 cm ^{2}	0.4	0.903	1.665	2.181	-1.265	2.361	0.592	0.793
RIV 10 cm ^{2}vs RIV 15 cm ^{2}	-0.800	1.041	3.135	515.744	-3.935	515.745	0.994	0.764

All-cause adverse events for moderate to severe AD

Comparisons	Direct		Indirect		Differences			τ^2
	Log OR	SE	Log OR	SE	Log OR	SE	P-value	
PBO vs DON 10 mg	0.424	0.149	-0.248	40.213	0.672	40.213	0.987	1.48e-10
PBO vs MEM 20 mg	…	…	…	…	…	…	…	…
DON 10 mg vs MEM 20 mg	0.498	0.107	-0.924	98.448	1.422	98.449	0.988	1.65e-06
DON 10 mg vs MEM 20 mg +DON 10 mg	0.327	0.232	-0.834	146.045	1.161	146.045	0.994	6.26e-09

Efficacy on function

Comparisons	Direct		Indirect		Differences			τ^2
	SMD	SE	SMD	SE	SMD	SE	P-value	
PBO vs DON 10 mg	0.209	0.069	0.055	0.081	0.154	0.107	0.149	2.16e-09
PBO vs GAL 24 mg	…	…	…	…	…	…	…	…
PBO vs RIV 12 mg	0.162	0.084	0.275	0.084	-0.114	0.119	0.339	1.19e-09
PBO vs RIV 10cm ^{2}	0.234	0.087	0.261	0.126	-0.026	0.153	0.864	4.35e-08
PBO vs MEM 20 mg	0.101	0.052	0.157	0.145	-0.055	0.154	0.721	1.44e-10
PBO vs MEM 20 mg +DON 10 mg	0.501	0.190	0.260	0.104	0.242	0.217	0.265	4.15e-10
DON 10mg vs RIV 12 mg	0.107	0.066	-0.002	0.100	0.109	0.119	0.360	3.49e-08
DON 10mg vs MEM 20 mg	-0.048	0.122	-0.031	0.078	-0.016	0.144	0.911	1.03e-08
DON 10 mg vs MEM 20 mg +DON 10 mg	0.170	0.089	0.186	0.224	-0.016	0.241	0.946	1.52e-09
RIV 12 mg vs RIV 10 cm ^{2}	0.310	0.064	-0.053	0.228	0.084	0.237	0.723	4.28e-10
RIV 10 cm ^{2}vs RIV 15cm ^{2}	0.183	0.087	-0.237	28.224	0.420	28.224	0.988	7.35e-08
RIV 15 cm ^{2}vs RIV 5cm ^{2}	-0.137	0.078	-0.855	63.146	0.717	63.146	0.991	8.81e-10
MEM 20 mg vs MEM 20 mg +DON 10 mg	0.121	0.162	0.257	0.120	-0.136	0.203	0.503	3.38e-07

Efficacy on behavior

Comparisons	Direct		Indirect		Differences			τ^2
	SMD	SE	SMD	SE	SMD	SE	P-value	
PBO vs DON 10 mg	-0.157	0.075	0.062	0.127	-0.219	0.148	0.138	0.108
PBO vs GAL 24 mg	…	…	…	…	…	…	…	…
PBO vs RIV 12 mg	-0.040	0.154	-0.003	0.414	-0.037	0.442	0.934	0.127

PBO vs RIV 10cm ^{2}	-2.25e-08	0.154	-0.037	0.413	0.037	0.442	0.934	0.127
PBO vs MEM 20 mg	-0.041	0.070	-0.260	0.131	0.219	0.148	0.138	0.108
DON 10 mg vs MEM 20 mg	-0.103	0.107	0.116	0.103	-0.219	0.148	0.138	0.108
RIV 12 mg vs RIV 10 cm ^{2}	…	…	…	…	…	…	…	…

Efficacy on global changes

Comparisons	Direct		Indirect		Differences			τ^2
	Log OR	SE	Log OR	SE	Log OR	SE	P-value	
PBO vs DON 5 mg	0.751	0.152	-0.239	0.495	0.990	0.523	0.058	0.193
PBO vs DON 10 mg	0.688	0.127	2.336	0.722	-1.649	0.736	0.025	0.179
PBO vs GAL 24 mg	…	…	…	…	…	…	…	…
PBO vs GAL 32 mg	0.353	0.253	0.397	0.579	-0.045	0.635	0.944	0.232
PBO vs RIV 12 mg	0.614	0.179	0.309	0.436	0.305	0.471	0.518	0.228
PBO vs RIV 10cm ^{2}	0.276	0.205	0.856	0.345	-0.579	0.401	0.149	0.202
PBO vs RIV 5 cm ^{2}	0.393	0.326	0.379	0.724	0.014	0.802	0.986	0.235
PBO vs MEM 20 mg	…	…	…	…	…	…	…	…
DON 5mg vs DON 10 mg	0.245	0.147	-0.801	0.348	1.047	0.378	0.006	0.157
DON 10mg vs DON 23 mg	-0.102	0.216	-1.489	113.547	1.387	113.547	0.990	0.220
GAL 24 mg vs GAL 32 mg	0.089	0.248	0.044	0.585	0.045	0.635	0.944	0.232
RIV 12 mg vs RIV 10 cm ^{2}	-0.066	0.208	-0.398	0.381	0.332	0.434	0.444	0.225
RIV 10cm ^{2}vs RIV 5cm ^{2}	-0.039	0.315	-0.025	0.739	-0.014	0.802	0.986	0.235
RIV 5cm ^{2}vs RIV 15cm ^{2}	0.524	0.298	-0.820	157.021	1.344	157.021	0.993	0.220

Supplementary 10:
Comparison-adjusted funnel plot for each outcome from the network meta-analysis

Comparison-adjusted funnel plot of efficacy on cognition for mild to moderate AD

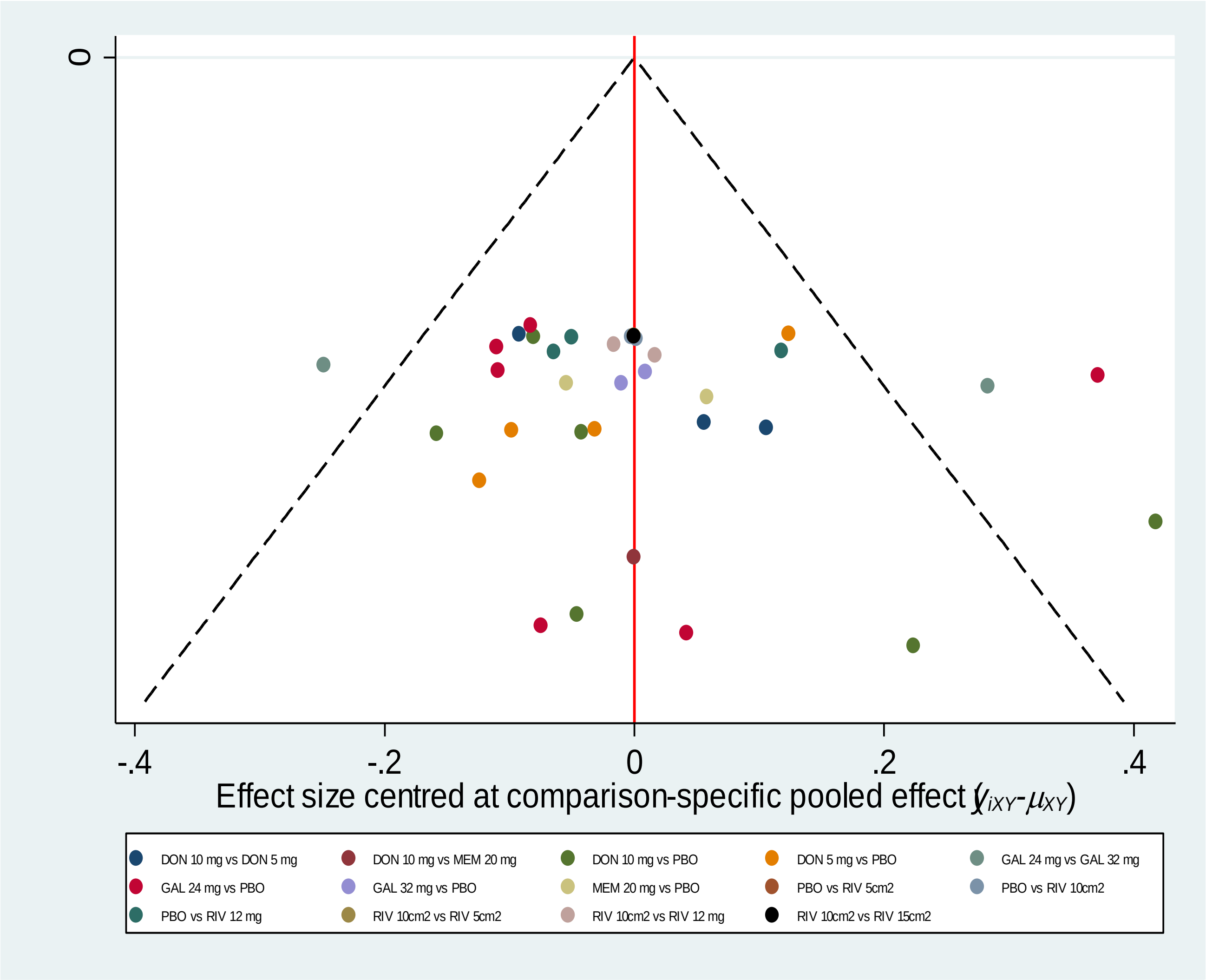

Comparison-adjusted funnel plot of efficacy on cognition for moderate to severe AD

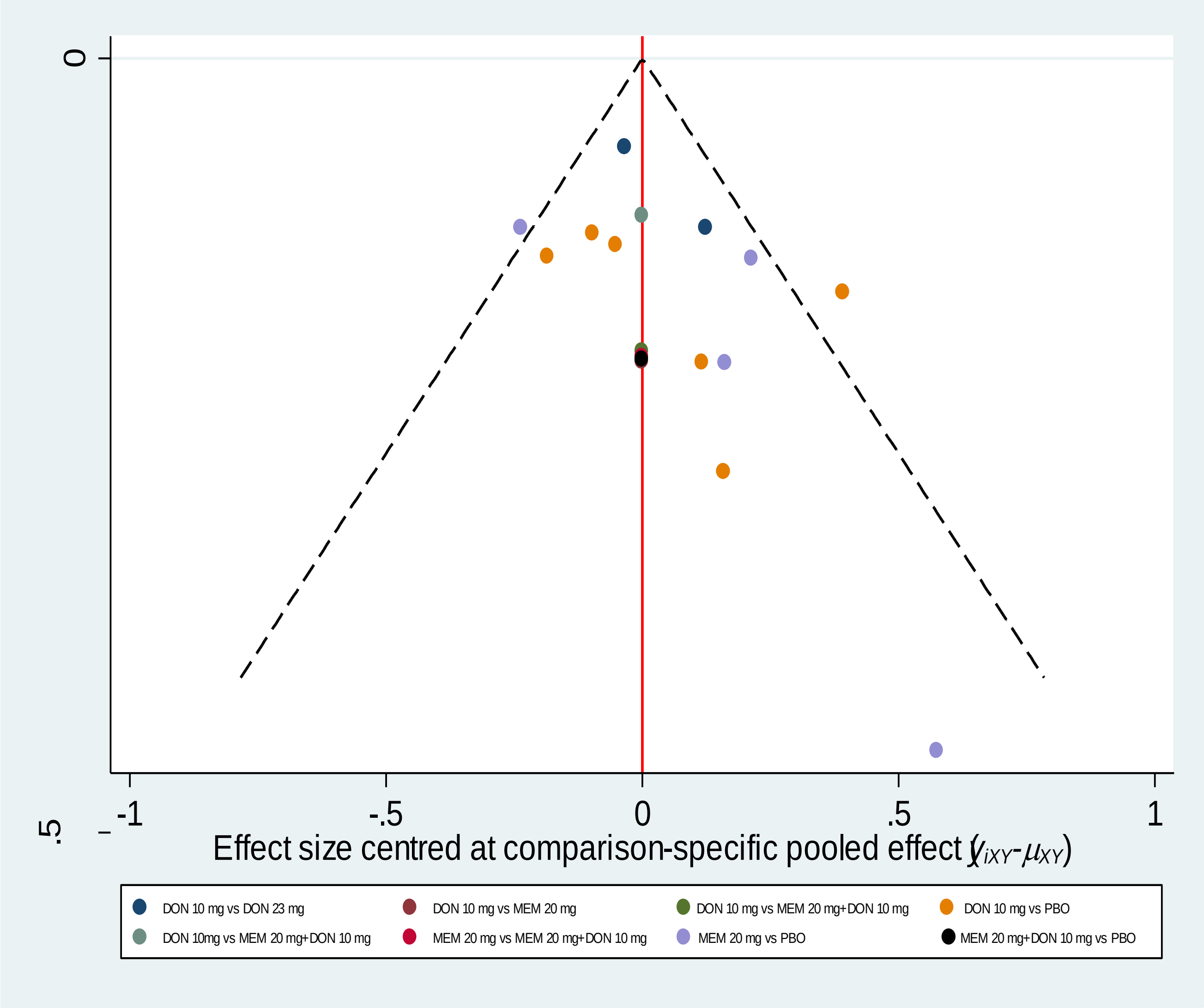

Comparison-adjusted funnel plot of tolerability for mild to moderate AD

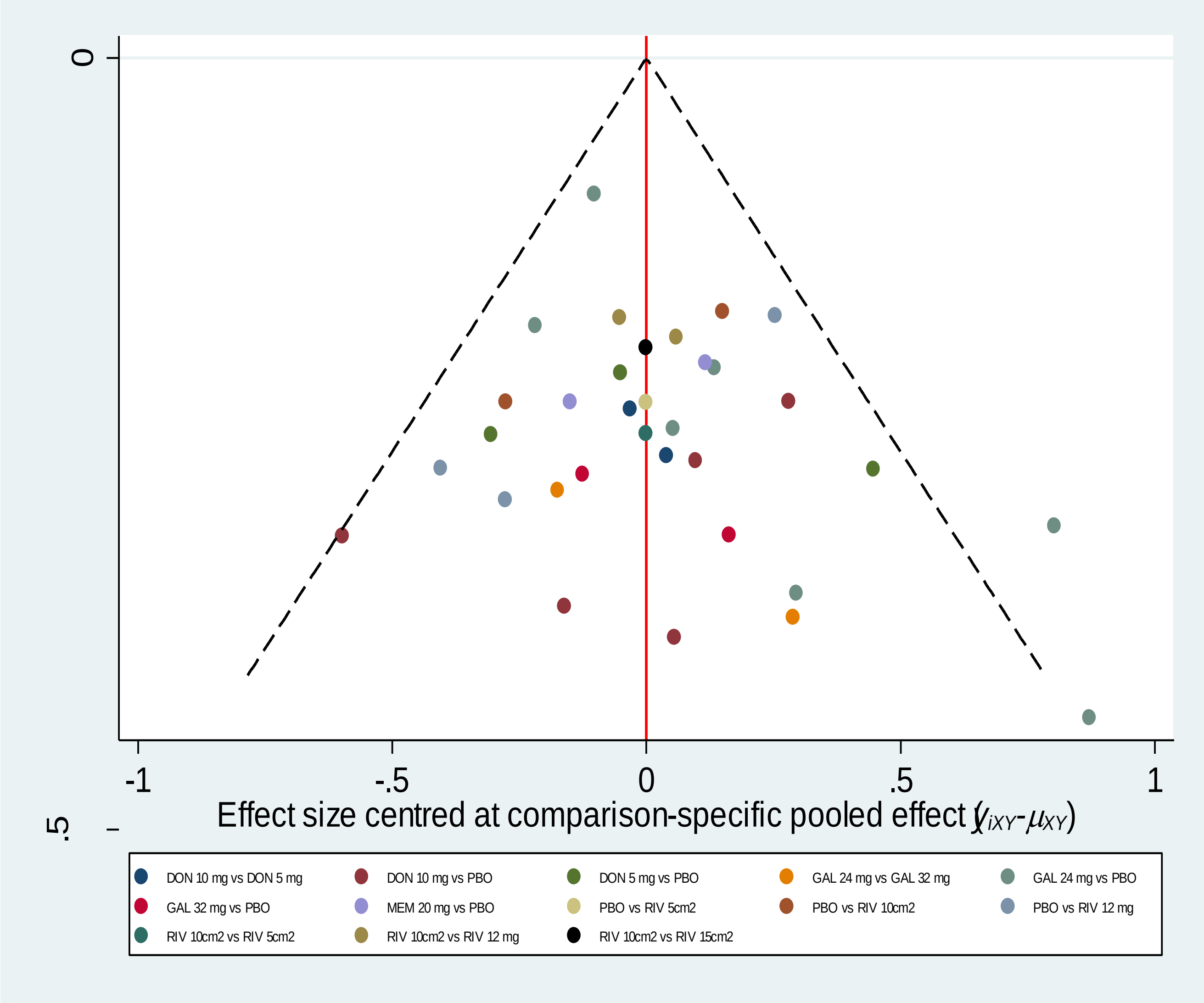

Comparison-adjusted funnel plot of tolerability for moderate to severe AD

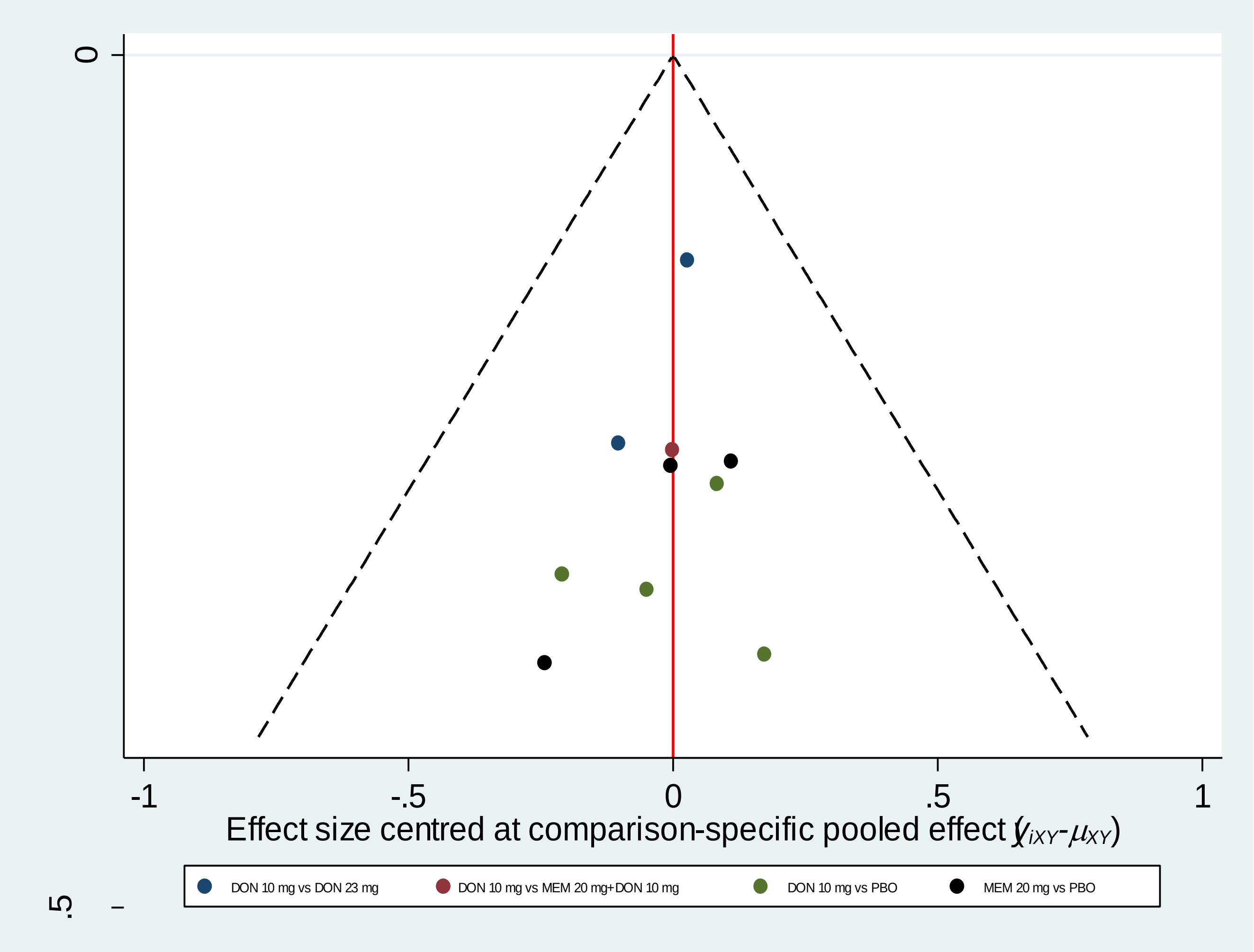

Comparison-adjusted funnel plot of efficacy on function

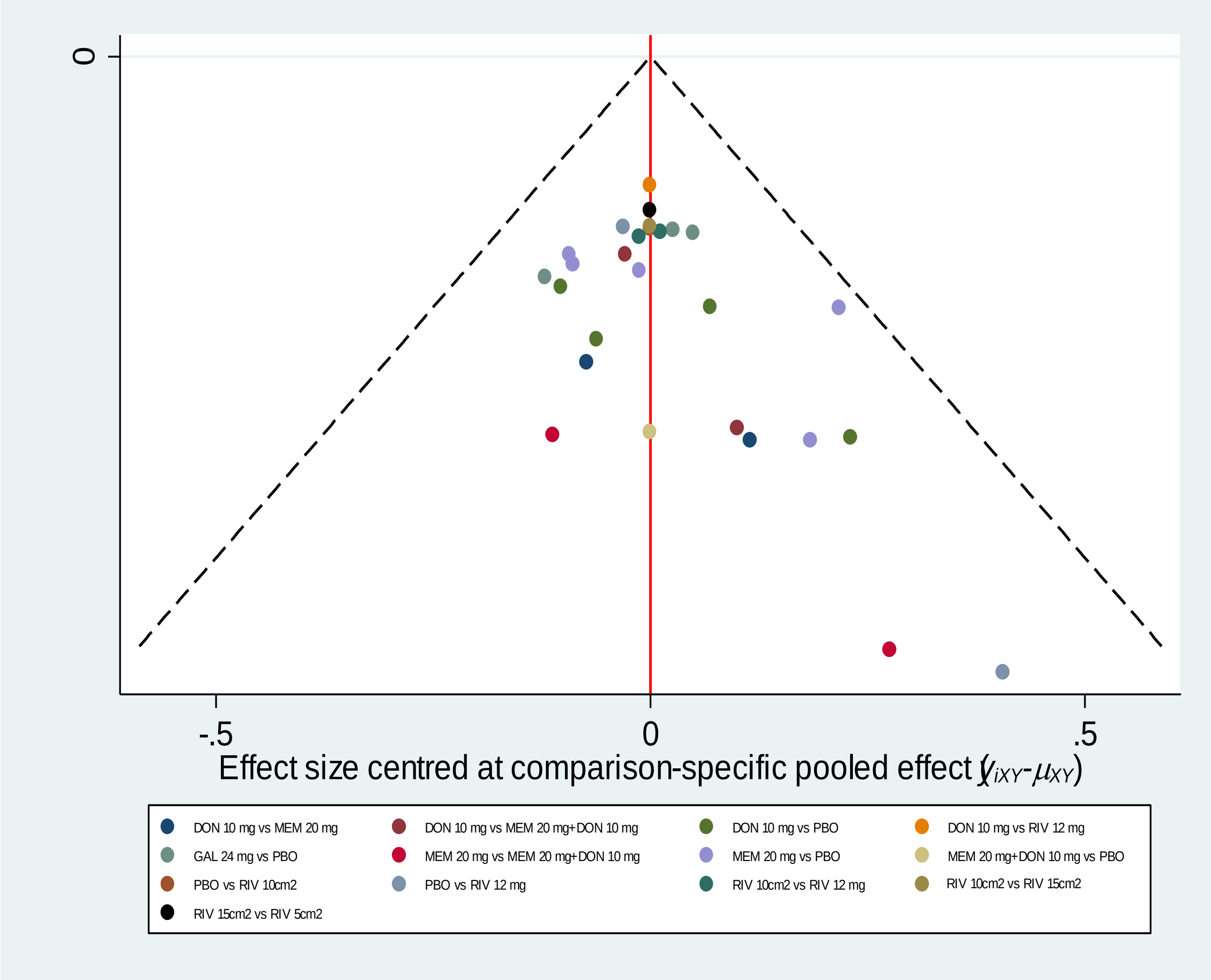

Comparison-adjusted funnel plot of efficacy on behavior

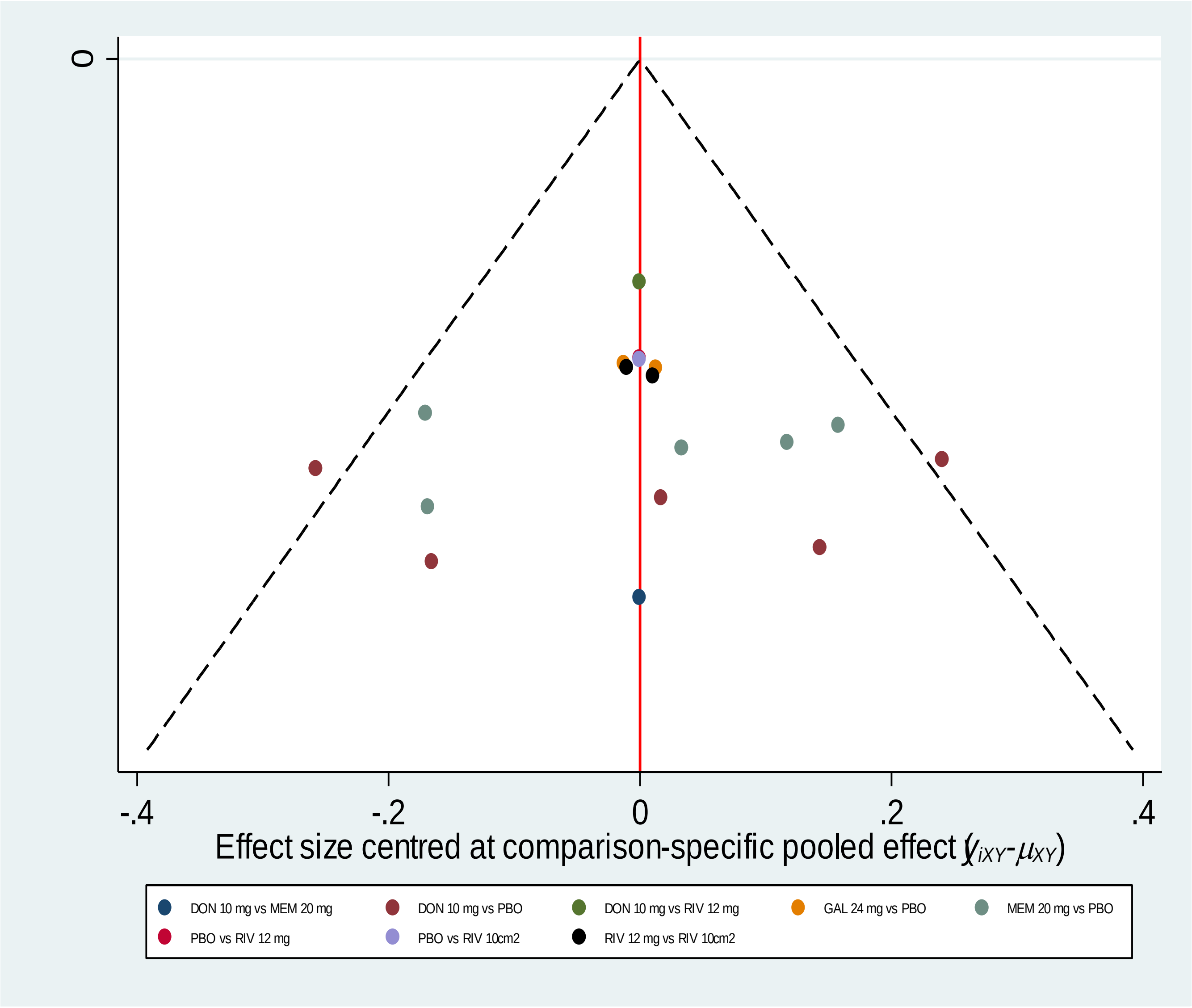

Comparison-adjusted funnel plot of efficacy on global changes

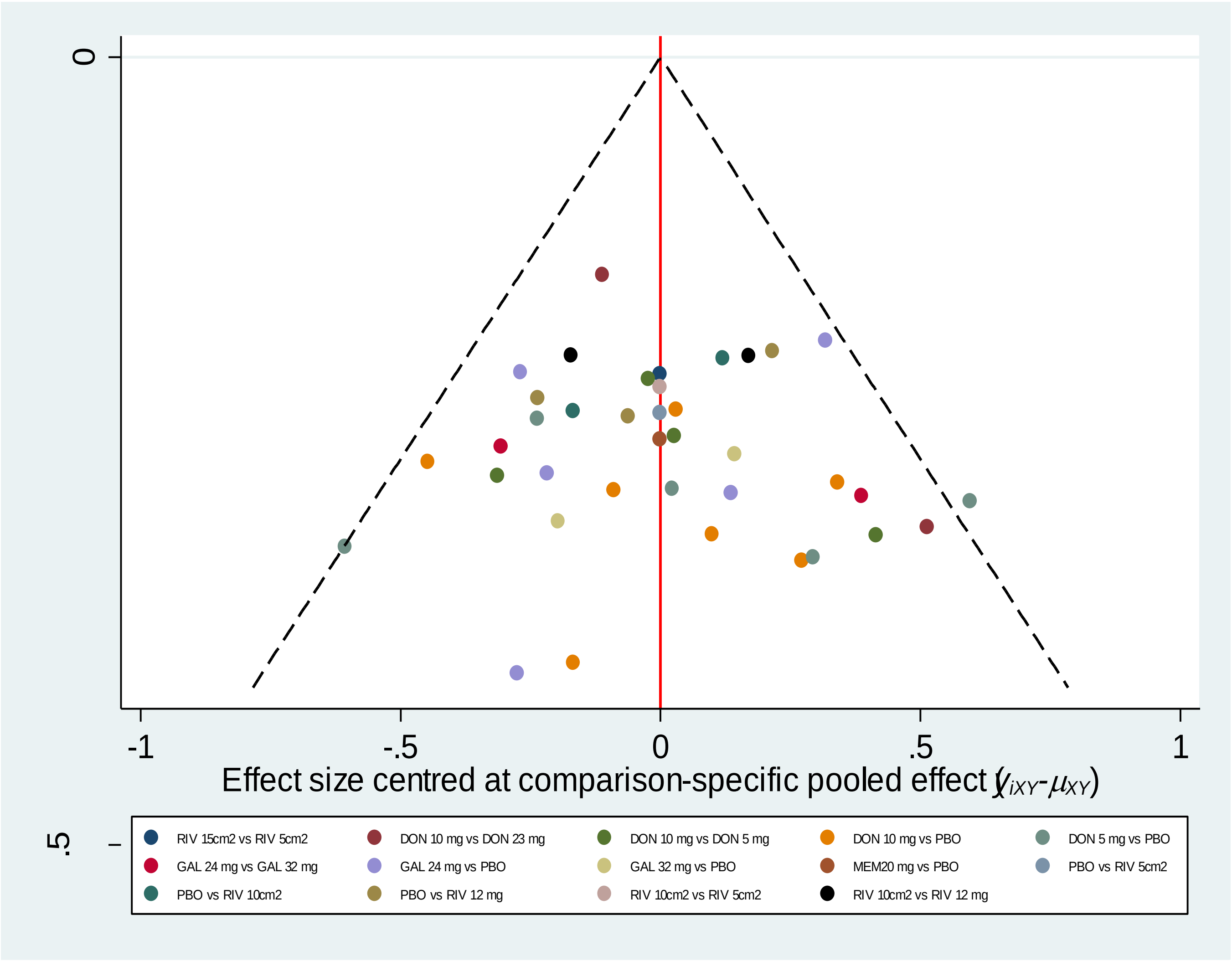

Supplementary 11:
Subgroup network meta-analysis for secondary outcomes

Efficacy on function for mild to moderate AD

NO.	Interventions
1	Placebo
2	Galantamine 24mg
3	Rivastigmine 12mg
4	Rivastigmine 10cm ²
5	Rivastigmine 15cm ²
6	Memantine 20mg

node	mean	sd	MC error	2.50%	median	97.50%	start	sample
SMD[1,2]	0.2507	0.1107	5.15E-04	0.04471	0.2507	0.4552	50001	300000
SMD[1,3]	0.1979	0.145	8.83E-04	-0.06983	0.1975	0.4678	50001	300000
SMD[1,4]	0.2286	0.1456	0.001003	-0.03855	0.228	0.5016	50001	300000
SMD[1,5]	0.4113	0.2129	0.001371	0.02037	0.4106	0.8064	50001	300000
SMD[1,6]	0.01232	0.1166	6.36E-04	-0.2041	0.01221	0.2276	50001	300000
SMD[2,3]	-0.05283	0.1829	0.001059	-0.389	-0.05267	0.2863	50001	300000
SMD[2,4]	-0.02214	0.1834	0.001153	-0.3604	-0.0221	0.3193	50001	300000
SMD[2,5]	0.1605	0.2405	0.001479	-0.2817	0.1611	0.6069	50001	300000
SMD[2,6]	-0.2384	0.161	7.97E-04	-0.5386	-0.2393	0.05716	50001	300000
SMD[3,4]	0.03069	0.1121	5.61E-04	-0.1754	0.03017	0.2379	50001	300000
SMD[3,5]	0.2134	0.1916	0.001001	-0.1415	0.2138	0.5663	50001	300000
SMD[3,6]	-0.1856	0.1859	0.001114	-0.5307	-0.1859	0.1572	50001	300000
SMD[4,5]	0.1827	0.1551	7.44E-04	-0.1041	0.1827	0.4676	50001	300000
SMD[4,6]	-0.2163	0.1862	0.001202	-0.5631	-0.215	0.1288	50001	300000
SMD[5,6]	-0.3989	0.2426	0.001528	-0.8503	-0.3976	0.04619	50001	300000

Efficacy on function for moderate to severe AD

NO.	Interventions
1	Placebo
2	Donepezil 10 mg
3	Galantamine 24 mg
4	Rivastigmine 12 mg
5	Memantine 20 mg
6	Memantine 20 mg+Donepezil 10 mg

node	mean	sd	MC error	2.50%	median	97.50%	start	sample
SMD[1,2]	0.1854	0.088	6.42E-04	0.01482	0.1846	0.361	50001	300000
SMD[1,3]	0.09063	0.1714	9.19E-04	-0.2487	0.09055	0.4304	50001	300000
SMD[1,4]	0.2225	0.1531	0.001088	-0.1237	0.2339	0.492	50001	300000
SMD[1,5]	0.1834	0.1048	6.48E-04	-0.02293	0.1828	0.3918	50001	300000
SMD[1,6]	0.3881	0.1383	0.001063	0.1163	0.3868	0.6642	50001	300000
SMD[2,3]	-0.09482	0.1923	0.001136	-0.4776	-0.09493	0.2842	50001	300000
SMD[2,4]	0.03707	0.1369	8.19E-04	-0.287	0.05128	0.2655	50001	300000
SMD[2,5]	-0.00203	0.1266	8.65E-04	-0.2525	-0.001441	0.2456	50001	300000
SMD[2,6]	0.2027	0.1248	8.84E-04	-0.04406	0.2028	0.4506	50001	300000
SMD[3,4]	0.1319	0.2298	0.001458	-0.3659	0.1433	0.5517	50001	300000
SMD[3,5]	0.09279	0.2004	0.001128	-0.3018	0.0929	0.4896	50001	300000
SMD[3,6]	0.2975	0.2201	0.001459	-0.1365	0.296	0.7349	50001	300000
SMD[4,5]	-0.03909	0.1801	0.001241	-0.3612	-0.05062	0.3604	50001	300000
SMD[4,6]	0.1656	0.1831	0.001264	-0.1568	0.1534	0.5732	50001	300000
SMD[5,6]	0.2047	0.1585	0.001219	-0.1045	0.2035	0.5199	50001	300000

Efficacy on behavior for mild to moderate AD

NO.	Interventions
1	Placebo
2	Donepezil 10 mg
3	Galantamine 24 mg
4	Rivastigmine 12 mg
5	Rivastigmine patch 10cm ²
6	Memantine 20 mg

node	mean	sd	MC error	2.50%	median	97.50%	start	sample
SMD[1,2]	-0.1	0.2294	7.95E-04	-0.5641	-0.0995	0.3639	50001	300000
SMD[1,3]	-0.1528	0.1941	4.97E-04	-0.5527	-0.1527	0.2471	50001	300000
SMD[1,4]	-0.03442	0.2576	9.57E-04	-0.5639	-0.034	0.495	50001	300000
SMD[1,5]	-0.00378	0.2572	9.98E-04	-0.5312	-0.00353	0.5241	50001	300000
SMD[1,6]	-0.1318	0.1796	5.62E-04	-0.5109	-0.1265	0.2222	50001	300000
SMD[2,3]	-0.05279	0.3	9.36E-04	-0.6619	-0.05261	0.5569	50001	300000
SMD[2,4]	0.06562	0.3452	0.001231	-0.638	0.06579	0.7721	50001	300000
SMD[2,5]	0.09625	0.3449	0.00127	-0.6048	0.09545	0.8005	50001	300000
SMD[2,6]	-0.03178	0.2312	7.26E-04	-0.5072	-0.02779	0.4217	50001	300000
SMD[3,4]	0.1184	0.3224	0.001067	-0.5469	0.1194	0.781	50001	300000
SMD[3,5]	0.149	0.3226	0.001105	-0.5146	0.1487	0.8108	50001	300000
SMD[3,6]	0.02101	0.264	7.60E-04	-0.5317	0.02689	0.5486	50001	300000
SMD[4,5]	0.03064	0.1939	3.98E-04	-0.3684	0.03048	0.4281	50001	300000
SMD[4,6]	-0.0974	0.3144	0.001112	-0.755	-0.09138	0.5336	50001	300000
SMD[5,6]	-0.128	0.3139	0.001161	-0.7823	-0.1213	0.5028	50001	300000

Efficacy on behavior for moderate to severe AD

NO.	Interventions
1	Placebo
2	Donepezil 10 mg
3	Memantine 20 mg

node	mean	sd	MC error	2.50%	median	97.50%	start	sample
SMD[1,2]	-0.123	0.1348	3.87E-04	-0.3919	-0.1239	0.1503	50001	300000
SMD[1,3]	-0.03901	0.1533	4.19E-04	-0.351	-0.03787	0.2683	50001	300000
SMD[2,3]	0.08395	0.2039	5.68E-04	-0.332	0.08609	0.4921	50001	300000

Efficacy on global changes for mild to moderate AD

NO.	Interventions
1	Placebo
2	Donepezil 5 mg
3	Donepezil 10 mg
4	Galantamine 24 mg
5	Galantamine 32 mg
6	Rivastigmine 12 mg
7	Rivastigmine patch 10cm ^{2}
8	Rivastigmine patch 5cm ^{2}

node	mean	sd	MC error	2.50%	median	97.50%	start	sample
or[1,2]	2.377	0.4047	0.002701	1.688	2.339	3.276	50001	300000
or[1,3]	2.52	0.4416	0.002795	1.755	2.485	3.492	50001	300000
or[1,4]	1.345	0.194	0.001268	0.9931	1.333	1.758	50001	300000
or[1,5]	1.479	0.3293	0.002106	0.9273	1.445	2.216	50001	300000
or[1,6]	1.79	0.2882	0.001509	1.295	1.765	2.431	50001	300000
or[1,7]	1.561	0.283	0.001554	1.082	1.535	2.193	50001	300000
or[1,8]	1.537	0.4422	0.002839	0.8556	1.477	2.564	50001	300000
or[2,3]	1.075	0.1858	0.001228	0.7469	1.061	1.477	50001	300000
or[2,4]	0.5818	0.1298	8.57E-04	0.3624	0.5702	0.8688	50001	300000
or[2,5]	0.6398	0.1798	0.001179	0.3531	0.618	1.054	50001	300000
or[2,6]	0.7745	0.1831	0.001135	0.4769	0.7545	1.193	50001	300000
or[2,7]	0.6756	0.1695	0.001045	0.4026	0.6561	1.063	50001	300000
or[2,8]	0.6651	0.2249	0.001429	0.3306	0.6314	1.192	50001	300000
or[3,4]	0.55	0.1263	8.28E-04	0.3425	0.536	0.8338	50001	300000
or[3,5]	0.605	0.1746	0.001145	0.3328	0.5813	1.011	50001	300000
or[3,6]	0.7323	0.1781	0.001065	0.4483	0.7114	1.142	50001	300000
or[3,7]	0.6387	0.1641	9.40E-04	0.3787	0.6187	1.017	50001	300000
or[3,8]	0.6291	0.2177	0.001384	0.3123	0.5954	1.146	50001	300000
or[4,5]	1.111	0.2448	0.001476	0.7047	1.086	1.663	50001	300000

or[4,6]	1.359	0.3013	0.00179	0.8781	1.324	2.052	50001	300000
or[4,7]	1.186	0.281	0.001675	0.7379	1.153	1.83	50001	300000
or[4,8]	1.167	0.3853	0.002321	0.6061	1.108	2.076	50001	300000
or[5,6]	1.271	0.359	0.002141	0.7223	1.221	2.116	50001	300000
or[5,7]	1.108	0.3272	0.001898	0.6125	1.064	1.877	50001	300000
or[5,8]	1.091	0.4104	0.002496	0.5135	1.022	2.075	50001	300000
or[6,7]	0.8827	0.1558	8.34E-04	0.6136	0.8701	1.226	50001	300000
or[6,8]	0.8744	0.2697	0.001645	0.4642	0.8365	1.502	50001	300000
or[7,8]	0.9991	0.2815	0.00158	0.5605	0.9635	1.649	50001	300000

Efficacy on global changes for moderate to severe AD

NO.	Interventions
1	Placebo
2	Donepezil 10mg
3	Donepezil 23mg

node	mean	sd	MC error	2.50%	median	97.50%	start	sample
or[1,2]	2.175	24.41	0.04983	0.6974	1.712	4.334	50001	300000
or[1,3]	42.9	18500	33.83	0.3418	1.531	5.941	50001	300000
or[2,3]	1.873	144.1	0.2663	0.2721	0.8985	2.477	50001	300000

Supplementary 12:
Sensitivity network meta-analysis for primary outcomes

Efficacy on cognition for mild to moderate AD

NO.	Interventions
1	Placebo
2	Donepezil 5 mg
3	Donepezil 10 mg
4	Glantamine 24 mg
5	Galantamine 32 mg
6	Rivastigmine 12 mg
7	Rivastigmine 10 cm ²
8	Rivastigmine 5 cm ²
9	Memantine 20 mg

node	mean	sd	MC error	2.50%	median	97.50%	start	sample
SMD[1,2]	-0.3192	0.06619	6.70E-04	-0.4517	-0.3192	-0.1901	50001	300000
SMD[1,3]	-0.4341	0.06251	6.68E-04	-0.5541	-0.4355	-0.3092	50001	300000
SMD[1,4]	-0.5208	0.05332	5.30E-04	-0.6258	-0.5209	-0.4174	50001	300000
SMD[1,5]	-0.523	0.07649	7.54E-04	-0.6747	-0.5226	-0.371	50001	300000
SMD[1,6]	-0.2927	0.05672	4.91E-04	-0.4044	-0.2929	-0.1807	50001	300000
SMD[1,7]	-0.2574	0.0637	5.85E-04	-0.3843	-0.2575	-0.1309	50001	300000
SMD[1,8]	-0.1689	0.09414	9.14E-04	-0.3553	-0.1693	0.01613	50001	300000
SMD[1,9]	-0.2412	0.07476	7.68E-04	-0.3895	-0.2402	-0.09634	50001	300000
SMD[2,3]	-0.115	0.07286	7.89E-04	-0.2541	-0.1171	0.03341	50001	300000
SMD[2,4]	-0.2016	0.08442	8.30E-04	-0.3677	-0.2021	-0.03541	50001	300000
SMD[2,5]	-0.2039	0.1006	9.93E-04	-0.4012	-0.2038	-0.005846	50001	300000
SMD[2,6]	0.02642	0.08739	8.39E-04	-0.1454	0.02628	0.1999	50001	300000
SMD[2,7]	0.06177	0.09113	8.37E-04	-0.118	0.06168	0.2434	50001	300000
SMD[2,8]	0.1502	0.1148	0.001135	-0.076	0.1506	0.3778	50001	300000
SMD[2,9]	0.078	0.09702	0.001006	-0.1119	0.07797	0.2686	50001	300000
SMD[3,4]	-0.08665	0.08174	8.56E-04	-0.2516	-0.08533	0.06984	50001	300000
SMD[3,5]	-0.08888	0.09886	0.00101	-0.2882	-0.08645	0.09974	50001	300000
SMD[3,6]	0.1414	0.08431	8.43E-04	-0.0269	0.1422	0.3037	50001	300000
SMD[3,7]	0.1768	0.0889	8.91E-04	-0.0015	0.1778	0.3477	50001	300000
SMD[3,8]	0.2652	0.1136	0.001161	0.03766	0.2671	0.4845	50001	300000
SMD[3,9]	0.193	0.08924	9.60E-04	0.01494	0.1948	0.366	50001	300000
SMD[4,5]	-0.002237	0.07458	7.49E-04	-0.1504	-0.002323	0.1446	50001	300000
SMD[4,6]	0.2281	0.07782	7.28E-04	0.07633	0.2279	0.3818	50001	300000
SMD[4,7]	0.2634	0.08281	7.63E-04	0.1023	0.2625	0.4274	50001	300000
SMD[4,8]	0.3519	0.1081	0.001051	0.1394	0.3516	0.5648	50001	300000
SMD[4,9]	0.2796	0.09144	9.03E-04	0.09971	0.2801	0.4606	50001	300000

SMD[5,6]	0.2303	0.09528	9.22E-04	0.04444	0.2304	0.4196	50001	300000
SMD[5,7]	0.2656	0.09951	9.48E-04	0.0696	0.2653	0.4628	50001	300000
SMD[5,8]	0.3541	0.1214	0.00118	0.1149	0.354	0.5942	50001	300000
SMD[5,9]	0.2819	0.1058	0.001006	0.07248	0.2817	0.4898	50001	300000
SMD[6,7]	0.03535	0.06423	5.88E-04	-0.0918	0.03585	0.1611	50001	300000
SMD[6,8]	0.1238	0.1026	9.63E-04	-0.0802	0.1234	0.3256	50001	300000
SMD[6,9]	0.05158	0.09414	9.43E-04	-0.135	0.05209	0.2338	50001	300000
SMD[7,8]	0.08847	0.09402	8.94E-04	-0.0987	0.08841	0.2731	50001	300000
SMD[7,9]	0.01623	0.09858	9.79E-04	-0.1795	0.01677	0.2083	50001	300000
SMD[8,9]	-0.07224	0.1205	0.001216	-0.3123	-0.07158	0.1645	50001	300000

Efficacy on cognition for moderate to severe AD

NO.	Interventions
1	Placebo
2	Donepezil 10 mg
3	Donepezil 23 mg
4	Memantine 20 mg
5	Memantine 20 mg + Donepezil 10 mg

node	mean	sd	MC error	2.50%	median	97.50%	start	sample
SMD[1,2]	0.5261	0.1654	5.73E-04	0.195	0.5246	0.8655	50001	300000
SMD[1,3]	0.5883	0.2785	8.68E-04	0.01865	0.5889	1.155	50001	300000
SMD[1,4]	0.3369	0.2113	6.59E-04	-0.05751	0.3254	0.7945	50001	300000
SMD[1,5]	0.8819	0.3608	0.001009	0.1509	0.8803	1.619	50001	300000
SMD[2,3]	0.06219	0.2244	4.71E-04	-0.4034	0.06528	0.5144	50001	300000
SMD[2,4]	-0.1891	0.2672	8.39E-04	-0.7004	-0.1987	0.378	50001	300000
SMD[2,5]	0.3558	0.3209	6.90E-04	-0.2986	0.3565	1.008	50001	300000
SMD[3,4]	-0.2513	0.35	0.001065	-0.9257	-0.264	0.4903	50001	300000
SMD[3,5]	0.2936	0.3921	8.23E-04	-0.5032	0.2913	1.098	50001	300000
SMD[4,5]	0.5449	0.4171	0.001163	-0.3301	0.5561	1.362	50001	300000

All-cause adverse events for mild to moderate AD

NO.	Interventions
1	Placebo
2	Donepezil 5 mg
3	Donepezil 10 mg
4	Glantamine 24 mg
5	Galantamine 32 mg
6	Rivastigmine 12 mg
7	Rivastigmine 10 cm ²
8	Rivastigmine 5 cm ²
9	Memantine 20 mg

node	mean	sd	MC error	2.50%	median	97.50%	start	sample
or[1,2]	1.428	0.3518	0.001627	0.8675	1.385	2.237	50001	300000
or[1,3]	1.787	0.409	0.002073	1.106	1.747	2.708	50001	300000
or[1,4]	1.587	0.2832	0.001546	1.126	1.554	2.242	50001	300000
or[1,5]	2.621	0.7083	0.003568	1.52	2.526	4.271	50001	300000
or[1,6]	2.847	0.5546	0.002814	1.971	2.778	4.127	50001	300000
or[1,7]	1.667	0.3489	0.001579	1.11	1.625	2.479	50001	300000
or[1,8]	1.799	0.6115	0.003219	0.9144	1.707	3.247	50001	300000
or[1,9]	1.037	0.2522	9.67E-04	0.6273	1.009	1.617	50001	300000
or[2,3]	1.305	0.3768	0.001872	0.7101	1.26	2.169	50001	300000
or[2,4]	1.177	0.3693	0.001654	0.6361	1.12	2.048	50001	300000
or[2,5]	1.943	0.7307	0.003346	0.9082	1.825	3.684	50001	300000
or[2,6]	2.111	0.6866	0.003108	1.118	2.005	3.729	50001	300000
or[2,7]	1.236	0.4123	0.001772	0.6348	1.173	2.208	50001	300000
or[2,8]	1.334	0.5821	0.002735	0.5592	1.232	2.722	50001	300000
or[2,9]	0.7692	0.2734	0.001153	0.3694	0.7291	1.41	50001	300000
or[3,4]	0.9355	0.2856	0.001428	0.5214	0.8898	1.618	50001	300000
or[3,5]	1.544	0.5689	0.002749	0.7434	1.446	2.912	50001	300000
or[3,6]	1.679	0.5335	0.002678	0.92	1.592	2.951	50001	300000
or[3,7]	0.9833	0.3226	0.001523	0.5255	0.9306	1.753	50001	300000
or[3,8]	1.061	0.4572	0.002374	0.4569	0.9774	2.167	50001	300000
or[3,9]	0.6112	0.213	9.57E-04	0.3046	0.5771	1.115	50001	300000
or[4,5]	1.679	0.4576	0.002404	0.9516	1.625	2.73	50001	300000
or[4,6]	1.847	0.4825	0.002086	1.089	1.787	2.963	50001	300000
or[4,7]	1.082	0.2944	0.001281	0.6181	1.046	1.763	50001	300000
or[4,8]	1.168	0.4486	0.002327	0.5289	1.098	2.224	50001	300000
or[4,9]	0.6734	0.2017	8.82E-04	0.3536	0.6499	1.135	50001	300000
or[5,6]	1.163	0.3984	0.001858	0.5915	1.101	2.105	50001	300000
or[5,7]	0.6814	0.2401	0.001102	0.3381	0.6428	1.248	50001	300000
or[5,8]	0.7354	0.3324	0.001667	0.2995	0.676	1.534	50001	300000
or[5,9]	0.4239	0.1581	7.09E-04	0.1962	0.3993	0.7957	50001	300000
or[6,7]	0.5954	0.1183	4.54E-04	0.3912	0.5856	0.8596	50001	300000
or[6,8]	0.6486	0.2354	0.001179	0.305	0.6141	1.199	50001	300000
or[6,9]	0.3773	0.1165	4.88E-04	0.1934	0.3637	0.6438	50001	300000
or[7,8]	1.102	0.3733	0.001883	0.5476	1.05	1.978	50001	300000
or[7,9]	0.6478	0.2085	8.66E-04	0.3274	0.6209	1.13	50001	300000
or[8,9]	0.6387	0.2743	0.001287	0.2656	0.5927	1.288	50001	300000

Supplementary 13:
Assessment of heterogeneity results for each outcome

Efficacy on cognition for mild to moderate AD

Comparisons	I ²pair	I ²cons	incons.P
Placebo vs Donepezil 5 mg	0%	0%	NA
Placebo vs Donepezil 10 mg	50.79%	42.86%	0.55
Placebo vs Galantamine 24 mg	0%	0%	NA
Placebo vs Galantamine 32 mg	0%	0%	NA
Placebo vs Rivastigmine 12 mg	34.59	26.66%	0.32
Placebo vs Rivastigmine 10 cm ²	0%	0%	0.49
Placebo vs Rivastigmine 5 cm ²	NA	NA	NA
Placebo vs Memantine 20 mg	0.96%	48.15%	0.10
Donepezil 5 mg vs Donepezil 10 mg	0%	39.48%	0.08
Donepezil 10 mg vs Memantine 20 mg	NA	25.63%	0.26
Galantamine 24 mg vs Galantamine 32 mg	0%	0%	NA
Rivastigmine 12 mg vs Rivastigmine 10 cm ²	0%	0%	0.34
Rivastigmine 10cm ²vs Rivastigmine 5cm ²	NA	NA	NA
Rivastigmine 10cm ²vs Rivastigmine 15cm ²	NA	NA	NA
Global I-squared: I ²pair: 0.06%; I ²cons: 0%			

All-cause adverse events for mild to moderate AD

Comparisons	I ²pair	I ²cons	incons.P
Placebo vs Donepezil 5 mg	49.15%	55.75%	NA
Placebo vs Donepezil 10 mg	19.72%	24.53%	NA
Placebo vs Galantamine 24 mg	63.20%	67.01%	NA
Placebo vs Galantamine 32 mg	0	0	NA
Placebo vs Rivastigmine 12 mg	58.30%	40.83%	0.56
Placebo vs Rivastigmine 10cm ²	40.99%	51.71%	0.17
Placebo vs Rivastigmine 5cm ²	NA	NA	NA
Placebo vs Memantine 20 mg	0	0	NA
Donepezil 5 mg vs Donepezil 10 mg	0	41.35%	0.09
Galantamine 24 mg vs Galantamine 32 mg	0	0	NA
Rivastigmine 12 mg vs Rivastigmine 10cm ²	0	0	0.27
Rivastigmine 10cm ²vs Rivastigmine 5cm ²	NA	NA	NA
Rivastigmine 10cm ²vs Rivastigmine 15cm ²	NA	NA	NA
Global I-squared: I ²pair: 40.42 %; I ²cons: 39.15%			

Efficacy on cognition for moderate to severe AD

Comparisons	I^2pair	I^2cons	incons.P
Placebo vs Donepezil 10 mg	73.50%	72.32%	NA
Placebo vs Memantine 20 mg	72.95%	79.58%	NA
Placebo vs Memantine 20 mg+Donepezil 10 mg	NA	95.09%	0.07%
Donepezil 10 mg vs Donepezil 23 mg	72.56%	72.56%	NA
Donepezil 10 mg vs Memantine 20 mg	NA	0	0.77%
Donepezil 10 mg vs Memantine 20 mg+Donepezil 10 mg	81.99%	88.48%	NA
Memantine 20 mg vs Memantine 20 mg+Donepezil 10 mg	NA	45.65%	0.48%
Global I-squared: I^2pair: 74.46 %; I^2cons: 80.12%			

All-cause adverse events for moderate to severe AD

Comparisons	I^2pair	I^2cons	incons.P
Placebo vs Donepezil 10 mg	0	0	NA
Placebo vs Memantine 20 mg	0	0	NA
Donepezil 10 mg vs Donepezil 23 mg	0	0	NA
Donepezil 10 mg vs Memantine 20 mg+Donepezil 10 mg	NA	NA	NA
Global I-squared: I^2pair: 0 %; I^2cons: 0%			

Efficacy on function

Comparisons	I^2pair	I^2cons	incons.P
Placebo vs Donepezil 10 mg	0%	17.03%	0.11
Placebo vs Galantamine 24 mg	61.55%	62.31%	NA
Placebo vs Rivastigmine 12 mg	79.94%	95.42%	NA
Placebo vs Rivastigmine patch $10cm^2$	NA	11.65%	0.33
Placebo vs Memantine 20 mg	0%	2.10%	0.28
Placebo vs Memantine 20 mg+Donepezil 10 mg	NA	44.93%	0.2
Donepezil 10 mg vs Rivastigmine 12 mg	NA	70.62%	0.1
Donepezil 10 mg vs Memantine 20 mg	0%	0%	0.69
Donepezil 10 mg vs Memantine 20 mg+Donepezil 10 mg	0%	0%	0.45
Rivastigmine 12 mg vs Rivastigmine patch $10cm^2$	0%	0%	NA
Rivastigmine $10cm^2$vs Rivastigmine $15cm^2$	NA	NA	NA
Rivastigmine $15cm^2$vs Rivastigmine $5cm^2$	NA	NA	NA
Memantine 20 mg vs Memantine 20 mg+Donepezil 10 mg	0%	61.92%	0.08
Global I-squared: I^2pair: 20.16 %; I^2cons: 55.35%			

Efficacy on behavior

Comparisons	I^2pair	I^2cons	incons.P
Placebo vs Donepezil 10 mg	56.30%	68.47%	0.02
Placebo vs Galantamine 24 mg	0%	0%	NA
Placebo vs Rivastigmine 12 mg	NA	NA	NA
Placebo vs Rivastigmine patch 10cm ^{2}	NA	NA	NA
Placebo vs Memantine 20 mg	53.75%	69.79%	0.01
Donepezil 10 mg vs Memantine 20 mg	0%	59.64%	0.11
Rivastigmine 12 mg vs Rivastigmine 10cm ^{2}	0%	0%	NA
Global I-squared: I^2pair: 41.40%; I^2cons: 48.64%			

Efficacy on global changes

Comparisons	I^2pair	I^2cons	incons.P
Placebo vs Donepezil 5 mg	50.24%	55.18%	NA
Placebo vs Donepezil 10 mg	0%	0	NA
Placebo vs Galantamine 24 mg	33.93%	34.54%	NA
Placebo vs Galantamine 32 mg	0%	0	NA
Placebo vs Rivastigmine 12 mg	6.81%	16.29%	NA
Placebo vs Rivastigmine 10cm ^{2}	0%	29.68%	0.17
Placebo vs Rivastigmine 5cm ^{2}	NA	NA	NA
Placebo vs Memantine 20 mg	NA	NA	NA
Donepezil 5 mg vs Donepezil 10 mg	0%	52.26%	0.02
Donepezil 10 mg vs Donepezil 23 mg	74.55%	76.06%	NA
Galantamine 24 mg vs Galantamine 32 mg	59.43%	59.66%	NA
Rivastigmine 12 mg vs Rivastigmine 10cm ^{2}	23.83%	57.65%	0.08
Rivastigmine 10cm ^{2}vs Rivastigmine 5cm ^{2}	NA	NA	NA
Rivastigmine 5cm ^{2}vs Rivastigmine 15cm ^{2}	NA	NA	NA
Global I-squared: I^2pair: 25.48%; I^2cons: 23.88%			
